# Nonlinear adaptive pose motion control of a servicer spacecraft in approximation with an accelerated tumbling target

**DOI:** 10.1038/s41598-024-65807-6

**Published:** 2024-06-28

**Authors:** Ali Kasiri, Farhad Fani Saberi

**Affiliations:** 1 Department of Aerospace Engineering, AMIRKABIR University of Technology, Tehran, Iran; 2 Aerospace Sciences and Technology Institute, AMIRKABIR University of Technology, Tehran, Iran

**Keywords:** Agile attitude maneuver, Large-angle attitude maneuver, Accelerated pose motion tracking, Adaptive control, Space rendezvous, Aerospace engineering, Mechanical engineering

## Abstract

Removing a limited number of large debris can significantly reduce space debris risks. These bodies are generally exposed to extreme environmental disturbance torques or consecutive accidents due to their large wet area, which causes them to experience accelerated high-rate tumbling motion. The existing literature has adequately explored the approximation operations with non-cooperative targets exhibiting 3-axis tumbling motion. However, the research gap lies in the lack of attention given to addressing this approximation for targets undergoing accelerated motion. Agile, accurate, and large-angle maneuvers are three common necessities for safely capturing such targets. Changes in the moment of inertia brought on by fuel slushing cannot be disregarded during such a maneuver. To deal with nonlinearities, adverse coupling effects, actuator saturation constraints, time-varying moment of inertia, and external disturbances that worsen during accelerated agile large-angle maneuvers, a novel adaptive control approach is developed in this paper. The controller's main advantage is its adjustable desired acceleration, which maintains its performance even when dealing with accelerated motion. The control law is directly synthesized from the nonlinear relative equations of motion, without any linearization or simplification of the system dynamics, making it robust to a variety of orbital elements and target behaviors. Adaptation laws are extracted from the Lyapunov stability theorem in a way that guarantees asymptotic stability. Moreover, control actuator roles (delay, saturation, and allocation) are accounted for in modeling and simulation. Finally, a comprehensive numerical simulation based on three different realistic and strict scenarios is carried out to demonstrate the effectiveness and performance of the proposed control approach. The controller's robustness against time-varying dynamic parameters (sharp and sudden change, smooth and slow change, and periodic change) is extensively demonstrated through simulation.

## Introduction

### Research problem

**Figure 1:**
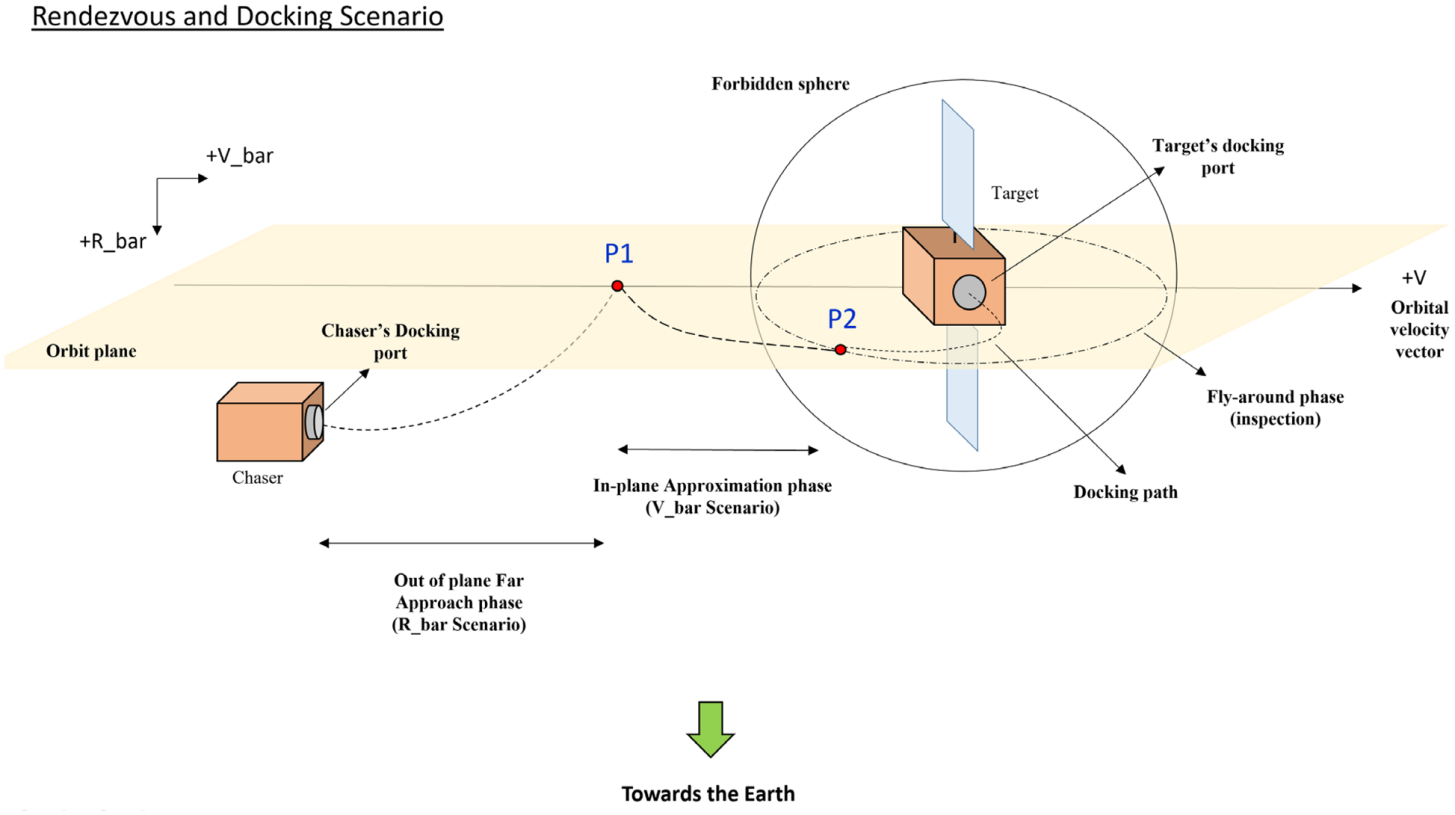
Schematic of rendezvous and docking sequence.

On-Orbit-Servicing (OOS) missions such as in-orbit refueling, in-orbit maintenance^1^, active debris removal^2,3^, and on-orbit assembly will occupy a unique position in the future of the space economy. This fact provided sufficient motivation to pass the academic research phase and enter the implementation stage. MEV (by Northrop Grumman), e.Deorbit (by ESA), O.CUBED (by AIRBUS), SSL (by MAXAR Technologies), and Phoenix (by DARPA) are just a few examples of OOS projects that are followed by great space companies or agencies all around the world^4,5^. Rendezvous and docking are the two common phases of the OOS missions. The rendezvous itself can be divided into (i) far-range (from the initial condition to P1 point) and (ii) close-range (from P1 to P2) approach sub-phases (see Fig. 1). It is quite understandable that the risk of collision increases as the distance between vehicles decreases, and for this reason, accuracy and robustness are especially important in close-range rendezvous^6^. Accordingly, unlike the long-range approach, in which only the translational distance reduction is a matter of concern, attitude synchronization is also necessary in addition to reducing the relative distance in the close-range approach^7^.

The behavior and specifications of the target have a significant impact on the challenges of the close-range approach phase. The target spacecraft can be categorized from the structure, cooperation, and stability viewpoints as follows:

#### Structure point of view

- A small rigid satellite (which doesn’t have any long, large, or flexible components)
- A large flexible satellite (which is equipped with extendable solar panels, antennas, large robotic manipulators, or other appendages)

#### Cooperation point of view

- Cooperative (which can transmit information/data about its attitude, velocity, and position)
- Non-cooperative (which doesn’t communicate, or transmit data. It may not be a live satellite)

#### Stability point of view

- Stable (which can keep its attitude in a stable manner or even perform a requested/desired maneuver)
- Unstable/Tumbling (which is dead, or alive but uncontrollable due to a severe failure of its Attitude Control System (ACS))

Without losing generality, capturing a large non-cooperative tumbling target has become a hot topic that has attracted huge attention in the last decade^8^. Because, the efficacy of mitigating the risk of space debris can be significantly enhanced by selectively removing a limited number of big debris objects (like launcher fairings, dead heavy satellites, orbital transfer modules, and so on). The point is that due to their large area, these debris are exposed to both (i) large disturbance torques (atmospheric drag and solar radiation pressure) and (ii) frequent accidents, and therefore experience time varying accelerated motion. The final close approach with such a target is the most challenging part of the mission, which deserves more investigation^9^. Getting close to a large tumbling debris without enough accuracy and agility runs the chaser spacecraft directly contrary to the purpose of its mission. For a real example, when a servicer or delivery spacecraft is performing a docking or berthing mission in the proximity of the dead *Envisat*^10^, which has huge extendable solar panels, antennas, and many other external appendages, even a small control error or delay may cause a disastrous accident and produce countless amounts of debris. Consequently, in contrast to traditional space missions (remote sensing or communication), where preserving the attitude (usually in nadir condition) with high accuracy was the only crucial matter, the ability to make agile and large-angle attitude maneuvers are two additional requirements for chaser spacecraft that play an important role in OOS mission reliability/safety^11^. However, the impact of fuel slushing resulting from such maneuvers, which alter the chaser's moment of inertia, must not be disregarded. On the other hand, both the hardware (actuators) and software (control algorithm) parts of the ACS contribute to satisfying the mission requirements (accuracy, agility, and robustness).

##### Hardware part considerations

Reaction Wheels (RW) are reliable, accurate, and cost-effective actuators that produce smooth and continuous torque in a wide range (regardless of the orbital elements)^12^. As a result, RWs are the most suitable attitude actuators for the final close-range approach (also called “approximation”) phase of the OOS missions, whose accuracy and agility are two essential requirements^13^. At least 3 orthogonal reaction wheels are needed for full 3-axis attitude control^14^. However, many spacecraft benefit from 4 RWs to achieve more reliability and extra maneuverability. Although 4 reaction wheels can be arranged in different configurations known as pyramidal, skew, and tetrahedral^15,16^, the pyramidal configuration has attracted more attention and been investigated comprehensively. In this regard, the tilt-angle of RWs in pyramidal configuration has been optimized to achieve minimum power consumption^17^, highest pointing accuracy^18^, highest momentum management performance^19^, and the widest angular-momentum envelope coverage ^20^. Zhang et at.^21^ compensated for the RWs misalignment (installation deviations) effects to perform high attitude precession. Reference^22^ proposed an optimal use of 4 RWs in the pyramid configuration to improve the agility performance of a rigid spacecraft using a novel minimum infinity-norm scheme. Hablani^23^ optimized the tilt-angle of the 4 RWs in pyramidal configuration to perform a specific sun-tracking mission with minimum power consumption and control effort.

After reading the research mentioned above, it seems that 4 reaction wheels arranged in a pyramidal configuration (shown in Fig. 5) might be the best choice for OOS missions as a tradeoff between mass considerations, accuracy, reliability, minimum power consumption, maximum torque envelope, and momentum management performance level.

Reaction thrusters are widely used for orbit transfer, station keeping, inclination correction, orbit raising, maintenance, and so on. Different types of thrusters (electrical, chemical, ion, cold gas, etc.)^24^, and modulators (PWM, PWPF, etc.)^25^ are available, which each of them is suitable for a specific mission. PWPF hydrazine-based thrusters are an appropriate option for the propulsion system of chaser/servicer spacecraft, due to the high operating frequency, precise and stable output thrust, high specific impulse, and wide range force they can produce^26^. We assumed that the chaser’s propulsion system is equipped with a hydrazine-based thruster array. In summary, the combination of 4 RWs and 16 thrusters forms a reasonable and reliable attitude and position control system for OOS missions.

**Figure 2:**
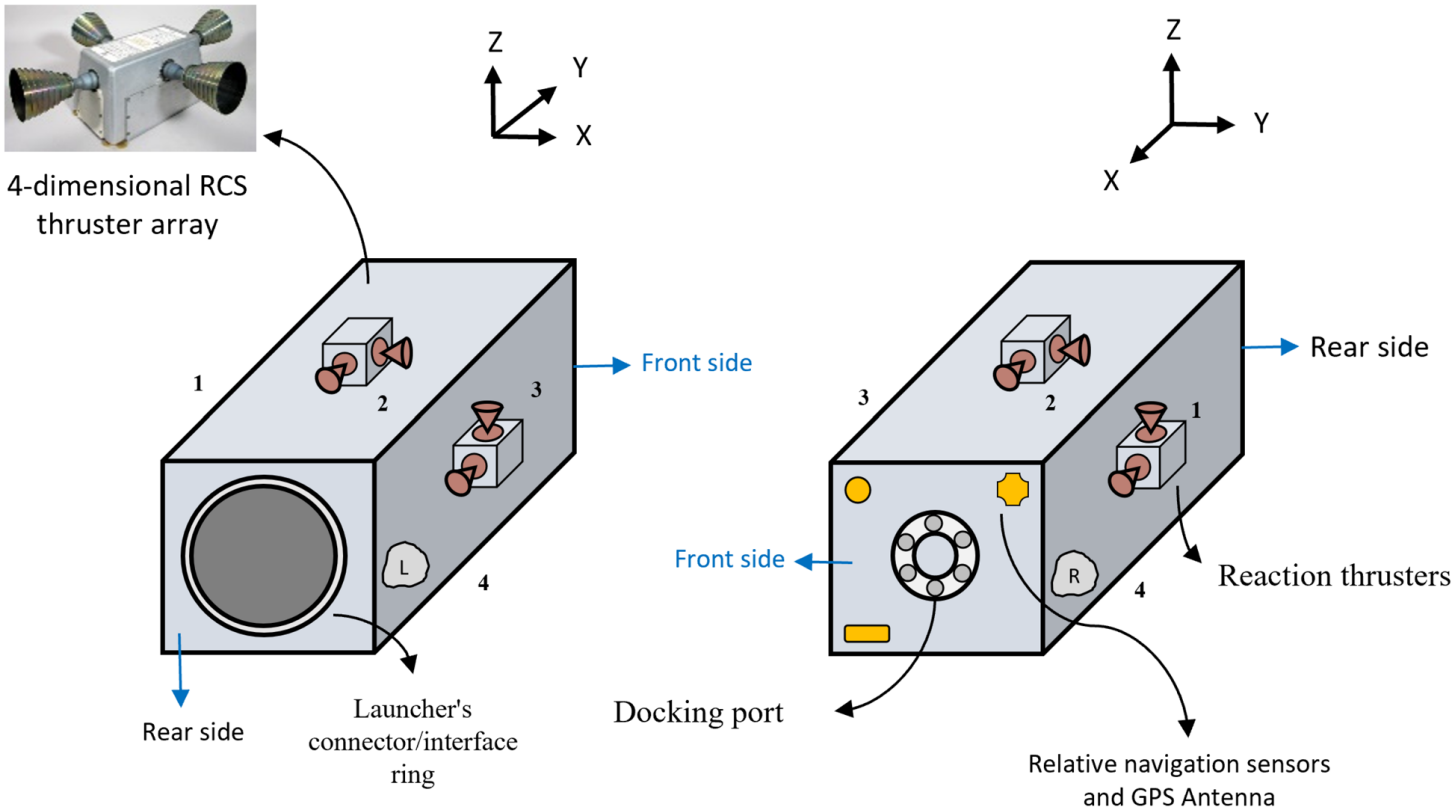
Schematic of chaser spacecraft.

Figure 2 shows the schematic of the chaser spacecraft from the front and aft views. As is clear, the chaser uses a set of 4-dimensional reaction thrusters in the side faces (right, left, up, and down). These thrusters can be used both for attitude control (for reaction wheel dumping/desaturation) and position control (producing pure force without torque). Different relative navigation sensors and antennas can be implemented on the front side.

One of the factors that can significantly affect the controller’s performance and also the system’s output is the actuator's model (delay, saturation level, etc.), although, despite its significance, this topic has not been covered in the majority of literature.

Note that the attitude determination subsystem is not restrictive for achieving high pointing accuracy because the combination of the star sensor and three-axis fiber optic gyroscope can provide an accuracy of about 3 to 30 arcseconds with stability of order 0.001 degrees per second.

##### Software part considerations

Relative equations of motion are highly nonlinear, and due to parametric uncertainties along with unknown external disturbances, classical controllers are not applicable anymore to meet the OOS mission requirements. Even all dynamic parameters might be known at the Beginning-of-Life (BoL), but uncertainty will arise gradually. Changes in mass, the moment of inertia, and the location of the CoM (caused by fuel consumption, internal mechanism activation, load and shape adjustments, and accidents) are particularly important here^27^. This issue may result in accuracy degradation or even instability, which is not permitted in OOS missions. We are interested in covering these issues without redesigning the controller’s structure. Given this fact, many researchers turned to adaptive control methods to deal with the problem.

### Literature review

A direct model reference adaptive control approach is used by^8^ to control the linearized orientational and translational motion of two Nano-satellites in proximity missions. But the linearized model (like CW or HCW model) has strong weaknesses/shortcomings when used for the close-range rendezvous phase (specifically in the face of an uncooperative tumbling target). Based on the nonlinear equations of motion expressed in spherical coordinates, Yoon et al.^28^ proposed an adaptive controller to cope with thrust misalignments and the chaser’s mass uncertainties. For spacecraft rendezvous and docking problems subject to parametric uncertainties, a novel switching controller is developed by combining a direct adaptive control approach, neural networks, and a backstepping technique^29^. However, forming a neural network with a large active region entails a complex structure and high calculation cost. In order to reduce the number of fuzzy rules and computational burden, an adaptive fuzzy controller is constructed^27^, so that proximity maneuvers are achieved in the presence of unknown model uncertainties and complex kinematic couplings. Despite the helpful results obtained in this work, actuator saturation was ignored, while the simulation results show high control effort. An anti-saturation sliding mode surface based on a modified auxiliary system is proposed in^30^ to guarantee the exponential convergence of tracking errors in the presence of inertia uncertainty and compensate for the actuator saturation-related constraint. In this work, the chaser’s unknown parameters were estimated based on a novel and ingenious adaptation law, and its main drawback was the chattering phenomenon. Sun et al.^31^ proposed an adaptive backstepping controller considering input saturation and full-state constraints. Both element-wise and norm-wise adaptive estimation techniques are used in this paper for handling parametric uncertainties, kinematic couplings, and matched and mismatched disturbances. An adaptive nonlinear control approach is developed by^32^ for synthesizing the relative pose controller of autonomous space proximity missions under actuator saturation, actuator faults, relative state constraints, dynamic couplings, parametric uncertainties, and unknown disturbances. Nevertheless, this strategy is rather challenging to implement due to the large number of controller parameters that should be tuned. The formulation of adaptive control laws typically necessitates extensive feedback, even full-state feedback, which demands the employment of diverse measurement sensors. Conversely, every measurement tool introduces errors, noise, miscalibration, bias, and the like, necessitating the utilization of intricate filters. To address this problem, an adaptive output feedback control (based on an indirect adaptive control strategy) is proposed by^33^ that minimizes the need for measurement sensors (beyond the relative navigation sensors such as rate gyro). But an adaptation law is extracted based on exact knowledge of the dynamic model of the system and is really sensitive to model uncertainty. Another bold flaw of this paper is the high-frequency fluctuation in outputs and control signals, which is not ideal for close-range approximation. To deal with the non-constant parameter uncertainty problem, Xia et al.^34^ developed a novel adaptive fault-tolerant control strategy for spacecraft rendezvous maneuvers in the presence of unknown time-varying inertia parameters.

Apart from the controller capabilities, a safe approaching trajectory is an essential need for the close-range rendezvous to prevent hard collisions with the target. On the other hand, radar and lidar-based navigation may not be suitable for very close distances. In this regard, motivated by the idea of an image-based path planning method proposed in^35^, reference^36^ presented an Image-based Field of View (IFoV) constraint to ensure the visibility of the target and introduce image features in the controller design. In this paper, a potential field is developed using the pixel coordinates of the image features to interpret the IFOV constraint. By integrating a novel Artificial Potential Function (APF) into the sliding mode technique, an adaptive control strategy is introduced by Ref.^36^ to realize the arrival of the chaser at the docking port without any risk of collision or loss of target features. Considering the docking port's Line of Sight (LoS), an Immersion and Invariance (I&I) adaptive pose control scheme based on artificial potential functions (APFs) is proposed by Ref.^37^ for constrained spacecraft proximity operations with a freely tumbling target, under mass and inertia uncertainties. A very high control effort, which leads to unreasonable fuel consumption, is the main drawback of this method that makes it unfair for OOS missions. Considering the spatial motion constraints and docking port’s LoS, Shao et al.^38^ presented a novel adaptive pose tracking control scheme for spacecraft proximity operations with a freely tumbling target.

In the field of capturing a non-cooperative target, without the need for real-time orbit determination or communication between two spacecraft, the autonomous rendezvous with a target orbiting in an elliptical orbit is addressed in Ref.^39^. The main weakness is that only relative translational motion equations have been considered in this work. However, attitude synchronization is of the utmost importance in close-range rendezvous that cannot be ignored at all. By proposing a globally defined relative pose motion model in the presence of couplings between translational and rotational dynamics, parametric uncertainties, and bounded external disturbances, a robust adaptive control approach is developed by Ref.^40^ for rendezvous with a non-cooperative tumbling target. Considering the gravity of two spacecraft, complete relative position dynamics developed in Ref.^41^. Then, a robust adaptive controller is designed to control the relative nonlinear pose motion of a chaser spacecraft with a tumbling non-cooperative target.

### Research gap

The desired angular or translational acceleration of the chaser’s maneuver could not be set/adjusted as an input in any of the earlier control approaches.

Beyond that, all the above-mentioned papers proposed a solution to a distinct problem. With respect to all this research, all of them have drawbacks in at least one of the following aspects:

1. The RW’s role/term has been neglected in the relative attitude equations of motion, while its gyroscopic torque is not negligible in fast and large-angle attitude maneuvers.
2. Most of the previous research has considered the simple rest-to-rest maneuver problem, which is far from OOS nature most of the time. Moreover, a rest-to-rest maneuver is not a good choice for challenging the controller's capabilities.
3. Only a few researches (e.g. Ref.^34^) have investigated the effects of time-varying parameters on controller performance during the docking phase.
4. A considerable body of literature has been devoted to the "Clohessy-Wiltshire" equations, which are valid exclusively in the context of far approach in a circular orbit.
5. Except for a few reviewed studies (such as Refs.^36,41^), approximation with a non-cooperative 3-axis tumbling target has not been investigated.

#### Contributions

This paper addresses the problem of pose motion control for a chaser spacecraft in a close-range approach with a non-cooperative tumbling target orbiting in an elliptic orbit. The chaser is assumed to be over-actuated equipped with 4 reaction wheels and 16 reaction thrusters as the rotational and translational motion actuators, respectively. The main contributions of this paper are as follows:

- To deal with the time-varying dynamic parameters (sharp and sudden change, smooth and slow change, and periodic change), actuator saturation constraint, and bounded external disturbances acting on chaser spacecraft during large-angle agile maneuvers, a novel adaptive control approach is developed.
- Unlike all controllers reviewed in the literature review, the controller introduced in this paper allows for the input of desired angular (${\dot{\omega }}_{d}$) and translational accelerations (${\dot{V}}_{d}$). This feature turning it into a particular case for tracking an accelerated desired motion. This term also gives more flexibility to the controller designer to adjust the gains of the control law, which may lead to faster time responses of the chaser and a lower steady state error. In fact, the controller is also capable of tracking the accelerated motion of the target, accurately. This is a major benefit for safely docking with a wide-body non-cooperative tumbling target that is under high perturbations.
- Model accuracy has a significant impact on model-based control algorithms, especially in the case of large-angle agile maneuvers where nonlinearity and coupling effects are very strong. Thus, reaction wheels’ terms along with their gyroscopic torque are taken into account in driving the relative attitude motion. Considering uncertainties and disturbances in the controller design procedure makes it robust at a variety of orbital altitudes and conditions. The designed controller exhibits two key advantages: it is free from chattering and doesn't need a large number of control parameters to tune.
- In this paper, the motion of the target satellite in an elliptical orbit, a more generic scenario, has been taken into consideration.
- The control law is directly synthesized from the nonlinear equations of motion, without any linearization or simplification of the system dynamics. The importance of this issue stems from the fact that the performance of the designed controller is not dependent on orbital elements or orbit shape.
- The controller uses a simple parameter adaptation mechanism instead of complex and computationally expensive estimation, system identification, prediction, or compensator algorithms. This makes the controller more lightweight (which is practically essential in the approximation phase) and efficient, while still maintaining its effectiveness. Also, the adaptation law/mechanism is not sensitive to the initial condition.
- We have also rewritten the equations of motion based only on the relative navigation parameters (relative distance, relative velocity and attitude), instead of absolute navigation parameters.

This work is organized as follows: Section "Mathematical modeling" presents the problem formulation within the relative pose equations of motion. The controller laws are developed in Section "Simulation". Section "Conclusion" presents the study case scenario considering a non-cooperative tumbling target. The performance evaluation of the controllers is also presented in this Section. Finally, the conclusion is outlined in Sect. 5.

### Problem statement

Due to the large distance in the far-range approach, there is no risk of a harsh collision between the chaser and the target spacecraft. The chaser's only task in this phase is to reduce the relative distance ($P1$ in Fig. 1). However, the problem is more delicate in the close-range approach, particularly when dealing with a tumbling wide-body target that is equipped with large solar panels and antennas (such as geostationary telecommunication satellites). In the case of a cooperative target, the chaser is aware of the location (latitude, longitude, and altitude), velocity (both translational and angular), and attitude of the target at every step-time, which makes the close-range approach navigation easier and more accurate (more information about navigation and guidance methods in space rendezvous missions can be found in Refs.^9,42,43^). In the case of a non-cooperative target, only relative navigation sensors (RADAR^44^, LIDAR^45^, or vision-based^46^) can be relied on, while each of them has its own limits, constraints, noise, and errors. The fly-around and inspection are the necessary steps to define the docking port/interface or proper capturing structure (usually a solar panel strut or York)^47^. The chaser may have to fly around the target several times while looking for a suitable docking port/interface. As part of this procedure, the chaser will need to do an accurate large-angle maneuver. Eventually, the two most important characteristics for chaser spacecraft to face a wide body non-cooperative tumbling target are compact size and agility. In real-world conditions, there are many extra challenges, such as fuel slushing, delay, noise, hardware operating frequency, actuator saturation, and so on. To wrap it up, all these issues create the need for a controller that is robust to uncertainties, time-varying parameters, and disturbances. The use of 16 high-frequency hydrazine-based thrusters gives the chaser the ability to perform integrated pose (i.e., concurrent position attitude) maneuvers^48^. 4 reaction wheels provide smooth and accurate attitude synchronization (pointing accuracy of about $0.1 [\text{deg}]$ and precision of about $0.001 [\text{deg}/\text{s}]$) in the last docking phase. Thrusters are also used for RWs momentum dumping (desaturation). The target spacecraft is subject to external disturbance torques and forces which cause its 6 DoF accelerated motion. One important characteristic of the controller that can boost the performance of relative motion synchronization is adjustable acceleration. This is the main gap/deficiency of the previous research. Also, model accuracy has a significant contribution to model-based controller performance level. Thus, we tried to cover the actuator's role in the equations of motion.

After finding the docking port/interface or other proper capturing structure, the chaser moves toward it and captures the target. It should be noted that the capturing and post-capture phases have distinct sub-phases that can be followed in related research^49–52^.

### Essential coordinate systems

There are 3 essential frames that should be defined for attitude motion.

1. *Earth Centered Inertia frame (ECI)*: is a Cartesian right-handed system with the origin at the center of the Earth. It doesn’t orient with the Earth and its longitudinal axis ${X}_{I}$ is toward the vernal equinox. ${Z}_{I}$ is toward the north pole, and ${Y}_{I}$ completes the system.
2. *Orbit Reference frame (R)*: its origin is fixed on the mass center of the spacecraft (no matter the chaser or target). Its longitudinal axis ${X}_{R}$ is toward the orbital velocity vector. ${Z}_{R}$ points toward the earth’s center and ${Y}_{R}$ completes the right-handed system.
3. *Body Fixed frame (B)*: its origin is fixed on the spacecraft’s Center of Mass (CoM). We assumed that the body frame matches/coincides with the body principal axes.

There is 1 more essential frame that should be defined for translational motion.

(4) *Local-Vertical Local-Horizontal frame (LVLH)*: its center is fixed to the target’s center of mass. ${X}_{L}$ is along the radius vector from Earth’s CoM to the target space. ${Y}_{L}$ corresponds to the velocity vector. ${Z}_{L}$ is perpendicular to the orbital plane in a way that completes the right-handed system.

**Figure 3:**
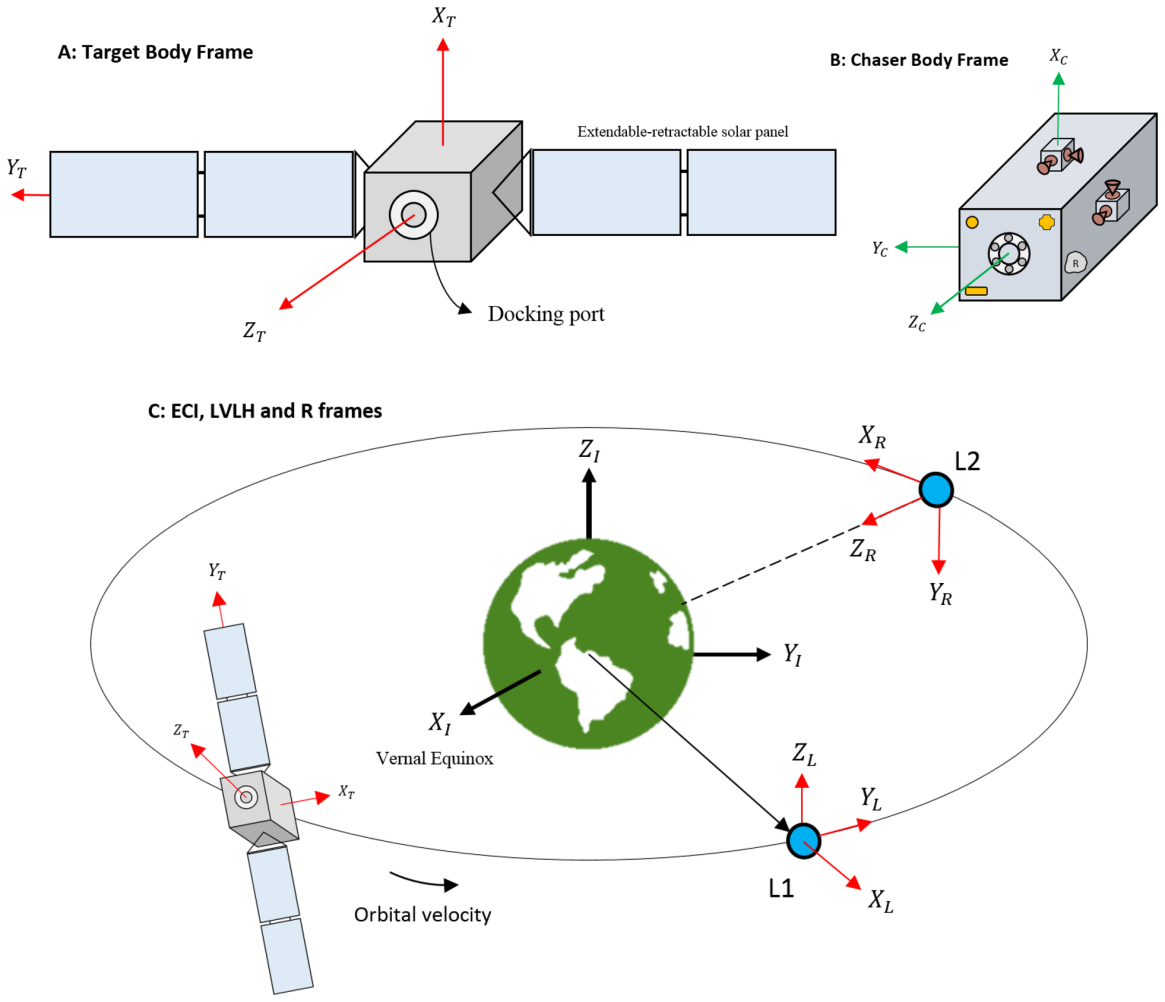
Schematic of different frames ((**A**) shows the target body frame, (**B**) shows the chaser body frame. ECI, LVLH and orbit reference frames are shown in (**C**)).

Introduced frames are shown in Fig. 3, schematically.

### Assumptions

**Figure 4:**
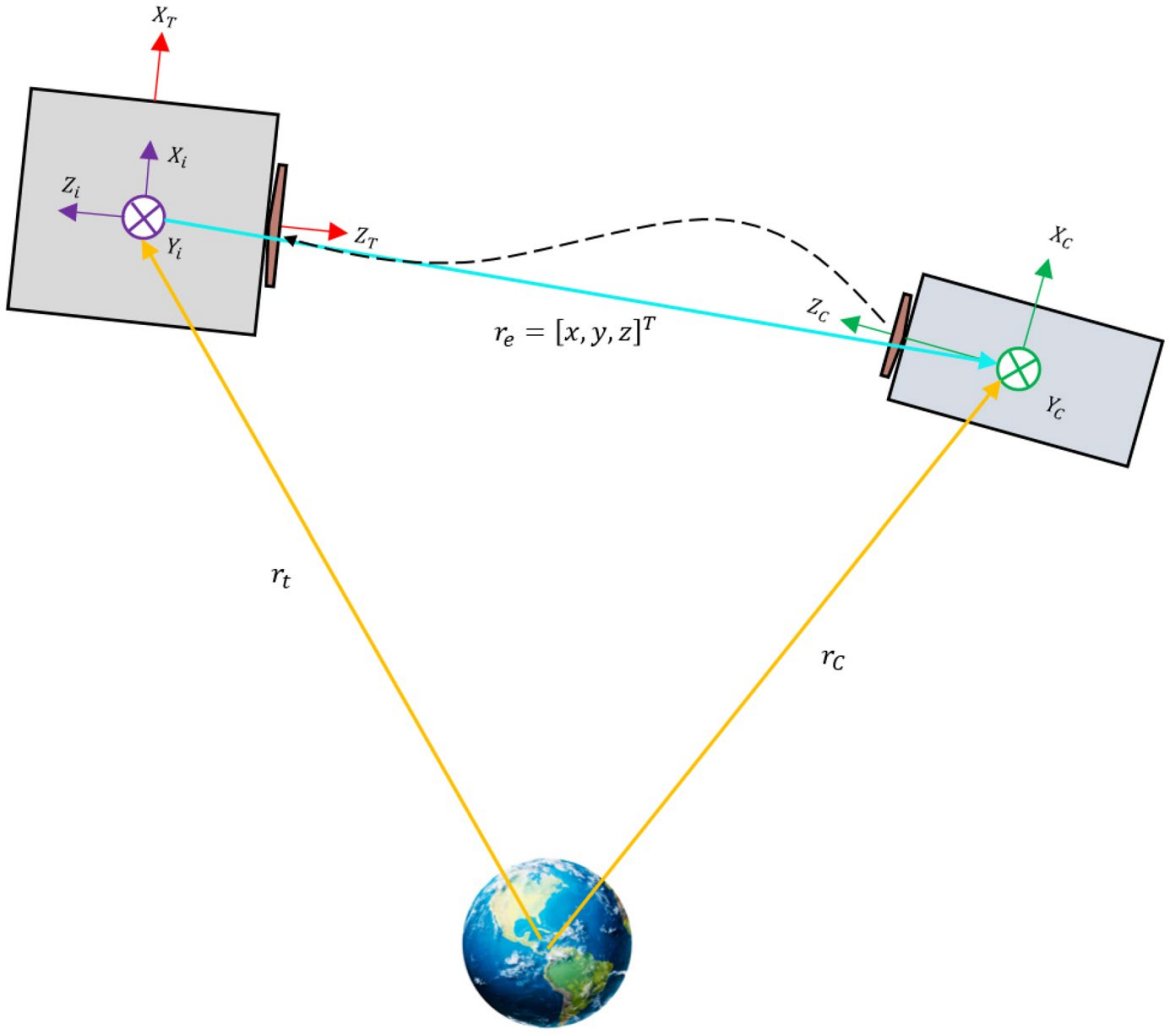
Chaser and target position vector.

- As shown in Fig. 4, the distance between the chaser and the target (${r}_{e}$) is very small compared to the target (${r}_{t}$) and the chaser (${r}_{C}$) spacecraft position vector $\left({r}_{e}\ll {r}_{t} \& {r}_{e}\ll {r}_{C}\right)$.
- The target orbit is elliptic.
- External disturbance forces and torques are unknown but bounded.
- The relative distance in the approximation phase is less than 100 m, and the relative navigation tools are capable of producing data with good accuracy.
- The chaser is equipped with all essential sensors and actuators: star trackers, fiber optic gyroscopes, GPS, reaction wheels, and thrusters. Thus, the absolute attitude, angular velocity, and position of the chaser are always available, and its pose motion is controllable independently and accurately.

## Mathematical modeling

In this section, the equations of rotational and translational motion will be derived.

### Attitude motion

The chaser can be modeled as a normal/nominal (healthy and actuated) spacecraft. The rotational dynamics of a spacecraft about its center of mass are simply given (expressed in the body frame) as follows:

1 $$\sum {\overrightarrow{M}}_{tot}={\dot{\overrightarrow{h}}}_{tot}+{\overrightarrow{\omega }}_{c}\times {\overrightarrow{h}}_{tot}$$

where ${\overrightarrow{M}}_{tot}={\left[{M}_{{tot}_{x}}, {M}_{{tot}_{y}}, {M}_{{tot}_{z}}\right]}^{T}\epsilon {\mathfrak{R}}^{3\times 1}$ is the total external (for example disturbance) and internal (for example thrusters) torques acting on the chaser spacecraft body. ${\overrightarrow{\omega }}_{c}={\left[{\omega }_{{c}_{x}}, {\omega }_{{c}_{y}}, {\omega }_{{c}_{z}}\right]}^{T}\epsilon {\mathfrak{R}}^{3\times 1}$ denotes the angular velocity vector of the chaser body with respect to the ECI frame, which is measured by Fiber Optic Gyroscopes (FOG) in real-world conditions. ${\overrightarrow{h}}_{tot}={\left[{h}_{{tot}_{x}}, {h}_{{tot}_{y}}, {h}_{{tot}_{z}}\right]}^{T}\epsilon {\mathfrak{R}}^{3\times 1}$ is the total angular momentum vector of the satellite about its CoM that is defined as follows:

2 $${\overrightarrow{h}}_{tot}={\overrightarrow{h}}_{c}+{\overrightarrow{h}}_{rw}$$

where ${\overrightarrow{h}}_{c}={\left[{h}_{{c}_{x}}, {h}_{{c}_{y}}, {h}_{{c}_{z}}\right]}^{T}\epsilon {\mathfrak{R}}^{3\times 1}$ and ${\overrightarrow{h}}_{rw}={\left[{h}_{{rw}_{x}}, {h}_{{rw}_{y}}, {h}_{{rw}_{z}}\right]}^{T}\epsilon {\mathfrak{R}}^{3\times 1}$ are the angular momentum vector of the chaser spacecraft’s body and RW’s, respectively.

${\overrightarrow{M}}_{tot}$ can be calculated as follows:

3 $${\overrightarrow{M}}_{tot}={\overrightarrow{M}}_{{GG}_{c}}+{\overrightarrow{M}}_{{r}_{c}}$$

where ${\overrightarrow{M}}_{GG}={\left[{M}_{{GG}_{x}}, {M}_{{GG}_{y}}, {M}_{{GG}_{z}}\right]}^{T}\epsilon {\mathfrak{R}}^{3\times 1}$ is the Gravity Gradient (GG) torque. Both chaser and target spacecraft have non-uniform/non-homogeneous mass distribution, this is the main cause of GG disturbance torque. The relation of GG disturbance torque for the target and the chaser is quite similar. Two symbols "${\overrightarrow{M}}_{{GG}_{c}}$" and "${\overrightarrow{M}}_{{GG}_{t}}$" will be shown so that they can be distinguished. Obviously, "${\overrightarrow{M}}_{{GG}_{c}}$" represents the torque applied to the chaser and "${\overrightarrow{M}}_{{GG}_{t}}$" represents the torque applied to the target.

The mathematical model of GG torque is given as^53^:

4 $$\left\{\begin{array}{c}{M}_{{GG}_{x}}=\frac{3\mu }{2{r}_{0}^{3}}\left({I}_{z}-{I}_{y}\right)\text{sin}\left(2\phi \right){\text{cos}}^{2}\left(\theta \right)\\ {M}_{{GG}_{y}}=\frac{3\mu }{2{r}_{0}^{3}}\left({I}_{z}-{I}_{x}\right)\text{sin}\left(2\theta \right)\text{cos}\left(\phi \right)\\ {M}_{{GG}_{z}}=\frac{3\mu }{2{r}_{0}^{3}}\left({I}_{x}-{I}_{y}\right)\text{sin}\left(2\theta \right){\text{cos}}^{2}\left(\phi \right)\end{array}\right.$$

where $\mu =\text{398600,435507 }{km}^{3}{s}^{-2}$ is the geocentric gravitational constant, $\theta$ and $\phi$ are the pitch and roll angles of the chaser/target spacecraft. ${I}_{x}$, ${I}_{y}$ and ${I}_{z}$ are the moment of inertia elements of the chaser/target spacecraft. ${r}_{0}$ denotes the distance between the center of the earth and the chaser/target spacecraft.

${\overrightarrow{M}}_{{r}_{c}}={\left[{M}_{{r}_{x}}, {M}_{{r}_{y}}, {M}_{{r}_{z}}\right]}^{T}\epsilon {\mathfrak{R}}^{3\times 1}$ denotes the random white noise signal with zero means (that acting on both spacecraft).

5 $${\overrightarrow{M}}_{{r}_{c}}=0.3randn(\text{3,1})$$

Finally, Eq. (1) can be rewritten as follows:

6 $${I}_{c}{\dot{\overrightarrow{\omega }}}_{c}=-S\left({\omega }_{c}\right){I}_{c}{\overrightarrow{\omega }}_{c}-S\left({\omega }_{c}\right){\overrightarrow{h}}_{rw}+{\overrightarrow{T}}_{c}+{\overrightarrow{M}}_{GG}+{\overrightarrow{M}}_{r}$$

where ${\overrightarrow{T}}_{c}=-{\dot{\overrightarrow{h}}}_{rw}={\left[{T}_{{c}_{x}}, {T}_{{c}_{y}}, {T}_{{c}_{z}}\right]}^{T}\epsilon {\mathfrak{R}}^{3\times 1}$ is the reaction wheels' control torque (will be calculated in section "Simulation"). ${I}_{c}=diag\left(\left[{I}_{{c}_{x}}, {I}_{{c}_{y}}, {I}_{{c}_{z}}\right]\right)$ denotes the chaser’s moment of inertia tensor. $S\left(\Xi \right)$ denotes the skew-symmetric matrix of the $\overrightarrow{\Xi }={\left[{\Xi }_{1}, {\Xi }_{2}, {\Xi }_{3}\right]}^{T}$ vector which is defined as follows:

7 $$S\left(\Xi \right)=\left[\begin{array}{ccc}0& {-\Xi }_{3}& {\Xi }_{2}\\ {\Xi }_{3}& 0& {-\Xi }_{1}\\ -{\Xi }_{2}& {\Xi }_{1}& 0\end{array}\right]$$

Quaternion representation is free from singularity and required low computational cost. Thus, it is the most useful method for kinematics modeling. $${\overrightarrow{q}}_{c}={\left[\begin{array}{cc}{q}_{{c}_{v}}^{T}& {q}_{{c}_{4}}\end{array}\right]}^{T} \epsilon {\mathfrak{R}}^{4\times 1}$$ is the chaser’s attitude quaternion. ${q}_{{c}_{4}}$ and ${\overrightarrow{q}}_{{c}_{v}}={\left[{q}_{{c}_{1}}, {q}_{{c}_{2}}, {q}_{{c}_{3}}\right]}^{T} \epsilon {\mathfrak{R}}^{3}\times \mathfrak{R}$ are the scaler part and vector component of the ${\overrightarrow{q}}_{c}$, respectively. In real-world conditions, quaternion parameters can be measured by star trackers directly (with an accuracy of 3 to 30 arc seconds).

Spacecraft kinematic equation based on quaternion parameters can be presented as follows:

8 $$\left[\begin{array}{c}{\dot{q}}_{{c}_{1}}\\ {\dot{q}}_{{c}_{2}}\\ {\dot{q}}_{{c}_{3}}\\ {\dot{q}}_{{c}_{4}}\end{array}\right]=\frac{1}{2}\left[\begin{array}{c}0\\ -{\omega }_{{c}_{z}}\\ {\omega }_{{c}_{y}}-{n}_{c}\\ -{\omega }_{{c}_{x}}\end{array} \begin{array}{c}{\omega }_{{c}_{z}}\\ 0\\ -{\omega }_{{c}_{x}}\\ -{\omega }_{{c}_{y}}-{n}_{c}\end{array} \begin{array}{c}-{\omega }_{{c}_{y}}+{n}_{c}\\ {\omega }_{{c}_{x}}\\ 0\\ -{\omega }_{{c}_{z}}\end{array} \begin{array}{c}{\omega }_{{c}_{x}}\\ {\omega }_{{c}_{y}}+{n}_{c}\\ {\omega }_{{c}_{z}}\\ 0\end{array}\right]\left[\begin{array}{c}{q}_{{c}_{1}}\\ {q}_{{c}_{2}}\\ {q}_{{c}_{3}}\\ {q}_{{c}_{4}}\end{array}\right]$$

where ${n}_{c}$ denotes the angular velocity of the chaser’s orbit (also called mean motion), which can be roughly calculated as follows:

9 $${n}_{c}=\sqrt{\mu /{r}_{c}^{3}}$$

where ${r}_{c}$ denotes the distance from the chaser’s CoM to the Earth’s CoM, respectively. Clearly, ${n}_{c}$ is a time-dependent variable for an elliptical orbit^54^ (because ${r}_{c}$ is not constant in this case).

Note: due to the close distance between the chaser and target spacecraft in the close-range approach phase we can assume that ${n}_{c}={n}_{t}$. The process of calculating ${n}_{t}$ will be discussed in section "Relative Translational Motion" (Eqs. (31) to (35)).

Without the loss of generality, the target’s attitude motion can be modeled in a similar way. The only difference is that there is no control torque for the target spacecraft.

The dynamics and kinematics of the target spacecraft are given as follows:

10 $${I}_{t}{\dot{\overrightarrow{\omega }}}_{t}=-S\left({\omega }_{t}\right){I}_{t}{\overrightarrow{\omega }}_{t}+{\overrightarrow{M}}_{{tot}_{t}}$$

11 $$\left[\begin{array}{c}{\dot{q}}_{{t}_{1}}\\ {\dot{q}}_{{t}_{2}}\\ {\dot{q}}_{{t}_{3}}\\ {\dot{q}}_{{t}_{4}}\end{array}\right]=\frac{1}{2}\left[\begin{array}{c}0\\ -{\omega }_{{t}_{z}}\\ {\omega }_{{t}_{y}}-{n}_{t}\\ -{\omega }_{{t}_{x}}\end{array} \begin{array}{c}{\omega }_{{t}_{z}}\\ 0\\ -{\omega }_{{t}_{x}}\\ -{\omega }_{{t}_{y}}-{n}_{t}\end{array} \begin{array}{c}-{\omega }_{{t}_{y}}+{n}_{t}\\ {\omega }_{{t}_{x}}\\ 0\\ -{\omega }_{{t}_{z}}\end{array} \begin{array}{c}{\omega }_{{t}_{x}}\\ {\omega }_{{t}_{y}}+{n}_{t}\\ {\omega }_{{t}_{z}}\\ 0\end{array}\right]\left[\begin{array}{c}{q}_{{t}_{1}}\\ {q}_{{t}_{2}}\\ {q}_{{t}_{3}}\\ {q}_{{t}_{4}}\end{array}\right]$$

where ${\overrightarrow{\omega }}_{t}={\left[{\omega }_{{t}_{x}}, {\omega }_{{t}_{y}}, {\omega }_{{t}_{z}}\right]}^{T}\epsilon {\mathfrak{R}}^{3\times 1}$ and ${I}_{t}=diag\left(\left[{I}_{{t}_{x}}, {I}_{{t}_{y}}, {I}_{{t}_{z}}\right]\right) \epsilon {\mathfrak{R}}^{3\times 3}$ are the angular velocity vector and moment of inertia tensor of the target, respectively. $${\overrightarrow{q}}_{t}={\left[\begin{array}{cc}{q}_{{t}_{v}}^{T}& {q}_{{t}_{4}}\end{array}\right]}^{T} \epsilon {\mathfrak{R}}^{3}\times \mathfrak{R}$$ denotes the quaternion parameters of the target spacecraft. ${\overrightarrow{M}}_{{tot}_{t}}={\left[{M}_{{tot}_{{t}_{x}}}, {M}_{{tot}_{{t}_{y}}}, {M}_{{tot}_{{t}_{z}}}\right]}^{T}\epsilon {\mathfrak{R}}^{3\times 1}$ is the external disturbance torque (gravity gradient (as mentioned in Eq. (4)), magnetic and random (as mentioned in Eq. (5))) applied on the target spacecraft.

Magnetic disturbance torque arises due to the interaction between the accumulated/residual magnetic field in the target (because of magnetorquers, batteries, electronic boards, and so on) and the magnetic field of the earth. The magnetic disturbance torque expressed in the target body frame can be estimated as^55^:

12 $${\overrightarrow{M}}_{{MM}_{t}}={\overrightarrow{M}}_{res}\times \overrightarrow{B}$$

where ${\overrightarrow{M}}_{res}={\left[\text{0.3,0.3,0.3}\right]}^{T} \epsilon {\mathfrak{R}}^{3\times 1} [{\text{Am}}^{2}]$ is assumed as the sum of the individual magnetic moments caused by permanent and induced magnetism and the spacecraft-generated loops. This parameter is obtained experimentally and is very dependent on the elements installed in the satellite. $\overrightarrow{B}$ denotes the magnetic field of the earth that can be estimated as follows^55^:

13 $$B=\frac{{\mu }_{\otimes }}{{r}_{0}^{3}}{\left(1+3{sin}^{2}\mathsf{\Theta }\right) \, }^\frac{1}{2}$$

where $\mathsf{\Theta }$ is the magnetic latitude measured from the magnetic equator, which can be considered as $90 [\text{deg}]$ in the worst case (at the magnetic pole). ${\mu }_{\otimes }=8.1\times {10}^{15} [{\text{Tm}}^{3}]$ represents the magnitude of the earth’s magnetic moment vector along the magnet axial direction^56^.

Note that the target is assumed to be a wide-body spacecraft operating in LEO, thus it is equipped with powerful batteries and magnetorquers. Therefore, its residual magnetic moment will be significant.

The total disturbance torque acting on the target is given as:

14 $${\overrightarrow{M}}_{{tot}_{t}}={\overrightarrow{M}}_{{GG}_{t}}+{\overrightarrow{M}}_{{r}_{t}}+{\overrightarrow{M}}_{{MM}_{t}}$$

Note that the GG and random disturbance (Eq. (4) and (5)) are common disturbances acting on both spacecraft. Although the relation of these disturbances is the same, however, the domain/intensity of them are different for the chaser and target. Thus, ${\overrightarrow{M}}_{{GG}_{c}}, {\overrightarrow{M}}_{{r}_{c}}$ denote disturbances acting on the chaser and ${\overrightarrow{M}}_{{GG}_{t}}, {\overrightarrow{M}}_{{r}_{t}}$ are disturbances acting on the target.

### Relative attitude motion

The relative navigation systems are installed on the chaser spacecraft. Thus, we are interested in developing the relative attitude motion in the chaser spacecraft body frame. As clear from Fig. 4, the docking port of the chaser spacecraft should point toward the docking port of the target. In other words, ${Z}_{C}$ must be aligned and in the same direction with $-{Z}_{T}$. To achieve this goal, we need to define a new frame called “interface frame”, which is obtained by rotating the target body frame by $+180 [\text{deg}]$ around ${X}_{T}$.

The rotation matrix from the target body frame to the interface frame can be represented by the following direct cosine matrix:

15 $${C}_{t}^{i}=\left[\begin{array}{ccc}1& 0& 0\\ 0& \text{cos}\left(\pi \right)& \text{sin}\left(\pi \right)\\ 0& -\text{sin}\left(\pi \right)& \text{cos}\left(\pi \right)\end{array}\right]=\left[\begin{array}{ccc}1& 0& 0\\ 0& -1& 0\\ 0& 0& -1\end{array}\right]$$

The quaternion parameters corresponding to this rotation can be written as follows:

16 $${\overrightarrow{q}}_{t\to i}={\left[{q}_{{t\to i}_{1}}, {q}_{{t\to i}_{2}}, {q}_{{t\to i}_{3}}, {q}_{{t\to i}_{4}}\right]}^{T}={\left[1, 0, 0, 0\right]}^{T}$$

The relative angular velocity (between chaser and interface frame) is given as:

17 $${\overrightarrow{\omega }}_{e}={\overrightarrow{\omega }}_{c}-{C}_{i}^{c}{\overrightarrow{\omega }}_{i}$$

where ${\overrightarrow{\omega }}_{e}={\left[{\omega }_{{e}_{x}}, {\omega }_{{e}_{y}}, {\omega }_{{e}_{z}}\right]}^{T}\epsilon {\mathfrak{R}}^{3\times 1}$ is the error angular velocity that should converge to zero to achieve the goal of attitude synchronization. ${\overrightarrow{\omega }}_{i}={\left[{\omega }_{{i}_{x}}, {\omega }_{{i}_{y}}, {\omega }_{{i}_{z}}\right]}^{T}\epsilon {\mathfrak{R}}^{3\times 1}$ is the interface frame angular velocity with respect to the ECI frame given as:

18 $${\overrightarrow{\omega }}_{i}={C}_{t}^{i}{\overrightarrow{\omega }}_{t}=\left[\begin{array}{ccc}1& 0& 0\\ 0& -1& 0\\ 0& 0& -1\end{array}\right]\left[\begin{array}{c}{\omega }_{{t}_{x}}\\ {\omega }_{{t}_{y}}\\ {\omega }_{{t}_{z}}\end{array}\right]={\left[{\omega }_{{t}_{x}}, -{\omega }_{{t}_{y}}, -{\omega }_{{t}_{z}}\right]}^{T}$$

${C}_{i}^{c}\epsilon {\mathfrak{R}}^{3\times 3}$ is the rotation matrix from the interface frame to the chaser body frame that can be calculated as follows:

19 $${C}_{i}^{c}={C}_{t}^{c}{C}_{i}^{t}$$

where ${C}_{i}^{t}=transpose({C}_{t}^{i})$ is a constant matrix, and ${C}_{t}^{c}$ is a time-varying matrix that denotes the rotation matrix from the target body to the chaser body frame given as follows^42^:

20 $${C}_{t}^{c}=\left[\begin{array}{ccc}{q}_{{r}_{1}}^{2}-{q}_{{r}_{2}}^{2}-{q}_{{r}_{3}}^{2}+{q}_{{r}_{4}}^{2}& 2\left({q}_{{r}_{1}}{q}_{{r}_{2}}+{q}_{{r}_{3}}{q}_{{r}_{4}}\right)& 2\left({q}_{{r}_{1}}{q}_{{r}_{3}}-{q}_{{r}_{2}}{q}_{{r}_{4}}\right)\\ 2\left({q}_{{r}_{1}}{q}_{{r}_{2}}-{q}_{{r}_{3}}{q}_{{r}_{4}}\right)& -{q}_{{r}_{1}}^{2}+{q}_{{r}_{2}}^{2}-{q}_{{r}_{3}}^{2}+{q}_{{r}_{4}}^{2}& 2\left({q}_{{r}_{2}}{q}_{{r}_{3}}+{q}_{{r}_{1}}{q}_{{r}_{4}}\right)\\ 2\left({q}_{{r}_{1}}{q}_{{r}_{3}}+{q}_{{r}_{2}}{q}_{{r}_{4}}\right)& 2\left({q}_{{r}_{2}}{q}_{{r}_{3}}-{q}_{{r}_{1}}{q}_{{r}_{4}}\right)& -{q}_{{r}_{1}}^{2}-{q}_{{r}_{2}}^{2}+{q}_{{r}_{3}}^{2}+{q}_{{r}_{4}}^{2}\end{array}\right]$$

where $${\overrightarrow{q}}_{r}={\left[\begin{array}{cc}{q}_{{r}_{v}}^{T}& {q}_{{r}_{4}}\end{array}\right]}^{T} \epsilon {\mathfrak{R}}^{3}\times \mathfrak{R}$$ represents the relative attitude quaternion between the target body and chaser body frame, which can be calculated as follows:

21 $${\overrightarrow{q}}_{r}={\overrightarrow{q}}_{t}^{-1}\otimes {\overrightarrow{q}}_{c}$$

The symbol $\otimes$ denotes quaternion multiplication.

Similarly, the error quaternion between the interface and chaser body frame can be calculated as:

22 $${\overrightarrow{q}}_{e}={\overrightarrow{q}}_{i}^{-1}\otimes {\overrightarrow{q}}_{c}$$

where ${\overrightarrow{q}}_{i}$ can be calculated from ${\overrightarrow{q}}_{t\to i}$ as follows:

23 $${\overrightarrow{q}}_{i}={\overrightarrow{q}}_{t}^{-1}\otimes {\overrightarrow{q}}_{t\to i}\Rightarrow {\overrightarrow{q}}_{i}=\left[\begin{array}{c}{q}_{{t}_{4}}\\ {q}_{{t}_{1}}\\ {q}_{{t}_{2}}\\ {q}_{{t}_{3}}\end{array} \begin{array}{c}-{q}_{{t}_{1}}\\ {q}_{{t}_{4}}\\ {q}_{{t}_{3}}\\ -{q}_{{t}_{2}}\end{array} \begin{array}{c}-{q}_{{t}_{2}}\\ -{q}_{{t}_{3}}\\ {q}_{{t}_{4}}\\ {q}_{{t}_{1}}\end{array} \begin{array}{c}-{q}_{{t}_{3}}\\ {q}_{{t}_{2}}\\ -{q}_{{t}_{1}}\\ {q}_{{t}_{4}}\end{array}\right]\left[\begin{array}{c}1\\ 0\\ 0\\ 0\end{array}\right]=\left[\begin{array}{c}{q}_{{t}_{4}}\\ {q}_{{t}_{1}}\\ {q}_{{t}_{2}}\\ {q}_{{t}_{3}}\end{array}\right]$$

Now, the attitude synchronization problem achieves if the attitude quaternion error (${\overrightarrow{q}}_{e}$) and angular velocity error (${\overrightarrow{\omega }}_{e}$) become zero together. Because the chaser should be synch with the interface frame not the target body frame.

Similar to Eq. (11), the relative attitude kinematics is a function of error angular velocity (${\overrightarrow{\omega }}_{e}$) and error quaternion parameters (${\overrightarrow{q}}_{e}$) as follows:

24 $$\left[\begin{array}{c}{\dot{q}}_{{e}_{1}}\\ {\dot{q}}_{{e}_{2}}\\ {\dot{q}}_{{e}_{3}}\\ {\dot{q}}_{{e}_{4}}\end{array}\right]=\frac{1}{2}\left[\begin{array}{c}0\\ -{\omega }_{{e}_{z}}\\ {\omega }_{{e}_{y}}\\ -{\omega }_{{e}_{x}}\end{array} \begin{array}{c}{\omega }_{{e}_{z}}\\ 0\\ -{\omega }_{{e}_{x}}\\ -{\omega }_{{e}_{y}}\end{array} \begin{array}{c}-{\omega }_{{e}_{y}}\\ {\omega }_{{e}_{x}}\\ 0\\ -{\omega }_{{e}_{z}}\end{array} \begin{array}{c}{\omega }_{{e}_{x}}\\ {\omega }_{{e}_{y}}\\ {\omega }_{{e}_{z}}\\ 0\end{array}\right]\left[\begin{array}{c}{q}_{{e}_{1}}\\ {q}_{{e}_{2}}\\ {q}_{{e}_{3}}\\ {q}_{{e}_{4}}\end{array}\right]$$

To extract the relative attitude dynamics, we need to calculate the time derivative of ${\overrightarrow{\omega }}_{e}$ from Eq. (17).

25 $${\dot{\overrightarrow{\omega }}}_{e}={\dot{\overrightarrow{\omega }}}_{c}+S\left({\omega }_{e}\right){C}_{i}^{c}{\overrightarrow{\omega }}_{i}-{C}_{i}^{c}{\dot{\overrightarrow{\omega }}}_{i}$$

Note that ${C}_{t}^{i}$ is a constant matrix $\left(\frac{d}{dt}\left({C}_{t}^{i}\right)=0\right)$, thus from Eq. (18) we have ${\dot{\overrightarrow{\omega }}}_{i}=\frac{d}{dt}\left({C}_{t}^{i}{\overrightarrow{\omega }}_{t}\right)={C}_{t}^{i}{\dot{\overrightarrow{\omega }}}_{t}$

Therefore, the Eq. (25) can be rewritten as follows:

26 $${\dot{\overrightarrow{\omega }}}_{e}={\dot{\overrightarrow{\omega }}}_{c}+S\left({\omega }_{e}\right){C}_{i}^{c}{\overrightarrow{\omega }}_{i}-{C}_{i}^{c}\left({C}_{t}^{i}{\dot{\overrightarrow{\omega }}}_{t}\right)$$

Substituting ${\dot{\overrightarrow{\omega }}}_{c}$ and ${\dot{\overrightarrow{\omega }}}_{t}$ from Eqs. (6) and (10) into Eq. (26), also replacing ${C}_{i}^{c}{\overrightarrow{\omega }}_{i}$ with ${\overrightarrow{\omega }}_{c}-{\overrightarrow{\omega }}_{e}$ (according to Eq. (17)) gives:

27 $${\dot{\overrightarrow{\omega }}}_{e}=f\left({\omega }_{rel}\right)+{I}_{c}^{-1}\left({\overrightarrow{T}}_{c}+{\overrightarrow{M}}_{tot}\right)-{I}_{t}^{-1}{C}_{t}^{c}{\overrightarrow{M}}_{{tot}_{t}}$$

where $f\left({\omega }_{rel}\right)$ is defined as follows:

28 $$f\left({\omega }_{rel}\right)=-{I}_{c}^{-1}\left[S\left({\omega }_{c}\right){I}_{c}{\omega }_{c}+S\left({\omega }_{c}\right){h}_{rw}\right]-S\left({\omega }_{c}\right){\omega }_{e}+{C}_{t}^{c}\left({I}_{t}^{-1}S\left({{C}_{t}^{c}}^{-1}\left({\omega }_{c}-{\omega }_{e}\right)\right)\right){I}_{t}\left({{C}_{t}^{c}}^{-1}\left({\omega }_{c}-{\omega }_{e}\right)\right)$$

As a summary, the relative nonlinear attitude motion is given as:

29 $${\left[\begin{array}{c}{\dot{\overrightarrow{\omega }}}_{e}\\ {\dot{\overrightarrow{q}}}_{{e}_{v}}\end{array}\right]}_{6\times 1}={\left[\begin{array}{c}f\left({\omega }_{rel}\right)\\ \frac{1}{2}\left({q}_{{e}_{4}}{\overrightarrow{\omega }}_{e}-S\left({\omega }_{e}\right){q}_{{e}_{v}}\right)\end{array}\right]}_{6\times 1}+{\left[\begin{array}{c}{I}_{c}^{-1}\\ 0\cdot {I}_{3}\end{array}\right]}_{6\times 3}{\left[{\overrightarrow{T}}_{c}+{\overrightarrow{M}}_{tot}\right]}_{3\times 1}-{\left[\begin{array}{c}{I}_{t}^{-1}{C}_{t}^{c}\\ 0\cdot {I}_{3}\end{array}\right]}_{6\times 3}{\left[{\overrightarrow{M}}_{{tot}_{t}}\right]}_{3\times 1}$$

### Relative translational motion

The relative translational equations of motion of the chaser with respect to the target spacecraft in LVLH frame are given as follows^57,58^:

30 $$\left\{\begin{array}{c}\ddot{x}=2{n}_{t}\dot{y}+{n}_{t}^{2}x+{\dot{n}}_{t}y+\frac{\mu }{{r}_{t}^{2}}-\frac{\mu \left({r}_{t}+x\right)}{{r}_{c}^{3}}+{C}_{c}^{LVLH}\frac{1}{{m}_{c}}{F}_{{c}_{x}}+{a}_{{d}_{x}}\\ \ddot{y}=-2{n}_{t}\dot{x}+{n}_{t}^{2}y-{\dot{n}}_{t}x-\frac{\mu }{{r}_{c}^{3}}y+{C}_{c}^{LVLH}\frac{1}{{m}_{c}}{F}_{{c}_{y}}+{a}_{{d}_{y}}\\ \ddot{z}=-\frac{\mu }{{r}_{c}^{3}}z+{C}_{c}^{LVLH}\frac{1}{{m}_{c}}{F}_{{c}_{z}}+{a}_{{d}_{z}}\end{array}\right.$$

where $x, y,$ and $z$ are the relative distance between the chaser and target spacecraft (${r}_{e} \epsilon {\mathfrak{R}}^{3\times 1}$ in Fig. 4) in 3 dimension. ${r}_{t} \epsilon {\mathfrak{R}}^{3\times 1}$ and ${r}_{c} \epsilon {\mathfrak{R}}^{3\times 1}$ are the position vector of the target’s and chaser’s CoM from the center of Earth, respectively. ${m}_{c}$ is the chaser's mass, and ${F}_{c}={\left[{F}_{{c}_{x}}, {F}_{{c}_{y}}, {F}_{{c}_{z}}\right]}^{T}\epsilon {\mathfrak{R}}^{3\times 1}$ denotes the control force produced by the chaser’s reaction thrusters. ${a}_{d}={\left[{a}_{{d}_{x}}, {a}_{{d}_{y}}, {a}_{{d}_{z}}\right]}^{T}\epsilon {\mathfrak{R}}^{3\times 1}$ denotes the relative disturbance acceleration. ${r}_{c}$ is available and measurable using GPS or ground station based navigation. ${r}_{t}$ is not available directly but is calculable as: ${r}_{t}={r}_{c}-{r}_{e}$ and ${r}_{e}$ is measurable using relative navigation systems^59^. ${C}_{c}^{LVLH}$ is the rotation matrix from the chaser body to the LVLH frame. ${n}_{t}$ is the target orbital angular velocity.

According to Eq. (30), the translational motion dynamic is coupled in the orbital plane (${X}_{L}$-${Y}_{L}$ plane) and is independent in out of plane (${Z}_{L}$).

Note: the following sequence can be followed for generating ${r}_{t}$ in simulation^56^.

Using a given time to fly from perigee, the mean anomaly (${M}_{e}$) can be calculated as follows:

31 $${M}_{e}=2\pi \frac{t}{T}$$

where $T$ is the orbital period.

Eccentric anomaly $\left(E\right)$ can be found by solving Kepler’s equation (based on Newton’s iterative method)

32 $$E-e\text{sin}\left(E\right)={M}_{e}$$

True anomaly $\left({\psi }_{true}\right)$ can be calculated as follows:

33 $$\text{tan}\left(\frac{{\psi }_{true}}{2}\right)=\sqrt{\frac{1+e}{1-e}}\text{tan}\left(\frac{E}{2}\right)$$

Finally, the target radius vector norm is:

34 $${r}_{t}=\frac{a\left(1-{e}^{2}\right)}{1+e\text{cos}\left({\psi }_{true}\right)}$$

Angular acceleration of an elliptical orbit can be calculated as:

35 $${\dot{n}}_{t}=-\frac{3}{2{r}^{5/2}}\sqrt{\mu }\frac{dr}{d{\psi }_{true}}\frac{d{\psi }_{true}}{dt}$$

36 $$\frac{dr}{d{\psi }_{true}}=\frac{ae\left(1-{e}^{2}\right)\text{sin}\left({\psi }_{true}\right)}{{\left(1+e\text{cos}\left({\psi }_{true}\right)\right)}^{2}}$$

### Disturbance force modeling

The most important disturbance forces acting on spacecraft in LEO are gravitational acceleration and atmospheric drag.

Earth is an oblate spheroid, which has a non-uniform/non-homogeneous mass distribution. For this reason, the satellite feels different gravity in different orbital positions (latitude). The perturbing gravitational acceleration vector $\left({\overrightarrow{a}}_{gravity}\right)$ acting on a spacecraft (expressed in the body frame) given as^56^:

37 $${\overrightarrow{a}}_{gravity}=\left(\frac{3}{2}\frac{\mu {J}_{2}{R}_{E}^{2}}{{\left|\overrightarrow{r}\right|}^{5}}{\left[{r}_{x}\left(5\frac{{r}_{x}^{2}}{{\left|\overrightarrow{r}\right|}^{2}}-1\right),{r}_{y}\left(5\frac{{r}_{y}^{2}}{{\left|\overrightarrow{r}\right|}^{2}}-1\right),{r}_{z}\left(5\frac{{r}_{z}^{2}}{{\left|\overrightarrow{r}\right|}^{2}}-3\right)\right]}^{T}\right)$$

where ${J}_{2}=0.00108262668$ is a constant known as the second zonal harmonic of the earth, and ${R}_{E}=6378 \left[\text{km}\right]$ is considered as the radius of the earth. $\overrightarrow{r}={\left[{r}_{x}, {r}_{y}, {r}_{z}\right]}^{T}\epsilon {\mathfrak{R}}^{3\times 1}$ is the position vector of spacecraft (chaser or target) from Earth’s CoM. In the end, ${\overrightarrow{a}}_{gravity}$ should transfer to the LVLH frame.

The atmospheric drag depends on the spacecraft's size, shape, and orbit altitude. Drag acceleration (${\overrightarrow{a}}_{drag}$) can be modeled as follows:

38 $${\overrightarrow{a}}_{drag}=\frac{1}{2}\frac{\rho {\left|{\overrightarrow{\text{v}}}_{D}\right|}^{2}S{C}_{D}}{m}\frac{{\overrightarrow{\text{v}}}_{D}}{\left|{\overrightarrow{\text{v}}}_{D}\right|}$$

where $\rho$ is the atmospheric density. ${C}_{D}$ indicates the drag coefficient, $S$ represents the area (cross-section) that is normal to the airflow, $m$ is the spacecraft (chaser or target) mass, and ${\overrightarrow{\text{v}}}_{D}$ is the spacecraft velocity vector with respect to the rotating atmosphere.

In LEO (from 0 to 1000 km), the atmosphere density $\left(\rho \right)$ can be calculated as^56^:

39 $$\rho ={\rho }_{0}EXP\left(-\frac{{h}_{ellp}-{h}_{0}}{H}\right)$$

where ${\rho }_{0}$ and ${h}_{0}$ are reference density and reference altitude, respectively. ${h}_{ellp}$ denotes the actual altitude of the orbit and $H$ is the scale height. These parameters can be found in reference^56^. The relative velocity vector can be calculated as follows:

40 $${\overrightarrow{\text{v}}}_{D}={\left.{\overrightarrow{\text{v}}}_{t/C}\right|}^{LVLH}-{\left.{\overrightarrow{\text{v}}}_{atm}\right|}^{LVLH}$$

where ${\left.{\overrightarrow{\text{v}}}_{atm}\right|}^{LVLH}$ is the velocity vector of the atmosphere, and ${\overrightarrow{\text{v}}}_{t/C}$ can be the velocity vector of the chaser or target spacecraft expressed in LVLH frame that is given as:

41 $${\left.{\overrightarrow{\text{v}}}_{t/c}\right|}^{LVLH}={\left[0, {v}_{{t/C}_{y}}, 0\right]}^{T}$$

42 $${v}_{{t/c}_{y}}=\sqrt{2\mu \left(\frac{1}{{r}_{0}}-\frac{1}{2a}\right)}$$

where ${v}_{{t/C}_{y}}$ denotes the target or chaser spacecraft velocity, which is moving in a Keplerian elliptical or circular orbit. ${r}_{0}$ and $a$ are the radius of (chaser or target) spacecraft from Earth center, and (chaser or target) orbit semi major axis, respectively.

The atmosphere is assumed to be fixed to the Earth and rotates with the Earth’s angular velocity. Thus, the velocity vector of the atmosphere at spacecraft altitude can be calculated as:

43 $${\overrightarrow{\text{v}}}_{atm}={\overrightarrow{\omega }}_{\oplus }\times {\overrightarrow{r}}_{t/C}$$

${\overrightarrow{r}}_{t/C}$ denotes the radial vector of the chaser or target spacecraft expressed in the ECI frame. and ${\overrightarrow{\omega }}_{\oplus }={\left[\text{0,0},7.26\times {10}^{-5}\right]}^{T} \epsilon {R}^{3\times 1}$ is the earth’s angular velocity in $\left[\text{rad}/\text{s}\right]$. Finally, using the rotation matrix from ECI to LVLH frame we have:

44 $${\left.{\overrightarrow{\text{v}}}_{atm}\right|}^{LVLH}={C}_{ECI}^{LVLH}{\overrightarrow{\text{v}}}_{atm}$$

The relative disturbance torque actually is the difference in disturbance forces acting on the chaser and target spacecraft (expressed in LVLH frame).

45 $${a}_{d}=\left({\overrightarrow{a}}_{{drag}_{t}}+{\overrightarrow{a}}_{{gravity}_{t}}\right)-\left({\overrightarrow{a}}_{d{rag}_{C}}+{\overrightarrow{a}}_{grav{ity}_{C}}\right)$$

Note: this paper assumes that the center of mass (CG) coincides with the aerodynamic center (ac), and therefore, atmospheric drag force does not generate any torque.

### Actuator modeling

**Figure 5:**
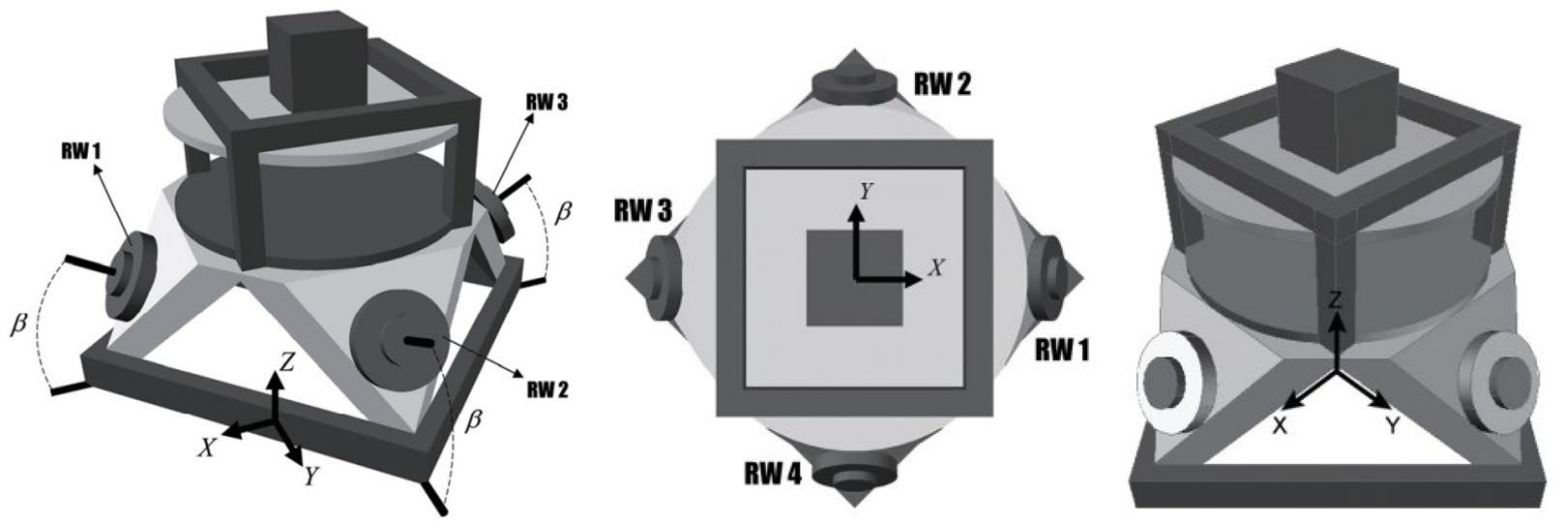
Reaction wheels’ tilt angle in pyramidal configuration.

A significant number of satellites utilize more than three reaction wheels both for extra maneuver capability and redundancy. Choosing four reaction wheels is actually a trade-off between the satellite’s overall reliability and mass considerations. Four reaction wheels can be arranged in different configurations, and this issue has a direct impact on ACS performance (torque envelope, momentum management, pointing accuracy, power consumption, and so on)^16^. Among various arrangements of the four reaction wheels, the pyramidal arrangement has attracted more attention and has been deeply investigated^17–19^. We assumed that the chaser is equipped with four reaction wheels installed in pyramidal configuration as shown in Fig. 5.

In Fig. 5, $\beta$ denotes the tilt-angle of wheels which plays an important role in wheels torque distribution. Regarding Fig. 5 the RWs control torque vector acting on the chaser body frame can be stated as follows.

46 $${\overrightarrow{T}}_{c}=\left[{A}^{*}\right]{\overrightarrow{T}}_{{w}_{i}}$$

47 $$\left[{A}^{*}\right]=\left[\begin{array}{c}\text{cos}\beta \\ 0\\ \text{sin}\beta \end{array} \begin{array}{c}0\\ \text{cos}\beta \\ \text{sin}\beta \end{array} \begin{array}{c}-\text{cos}\beta \\ 0\\ \text{sin}\beta \end{array} \begin{array}{c}0\\ -\text{cos}\beta \\ \text{sin}\beta \end{array}\right]$$

where ${\overrightarrow{T}}_{{w}_{i}}={\left[{T}_{{w}_{1}}, {T}_{{w}_{2}}, {T}_{{w}_{3}}, {T}_{{w}_{4}}\right]}^{T} \epsilon {\mathfrak{R}}^{4\times 1}$ is the reaction wheels torque vector.

Note that the components of RWs’ angular velocity (${\overrightarrow{\omega }}_{{w}_{i}}$) and momentum (${\overrightarrow{h}}_{{w}_{i}}$) along the principal body axes can also be calculated the same as Eq. (46).

**Figure 6:**
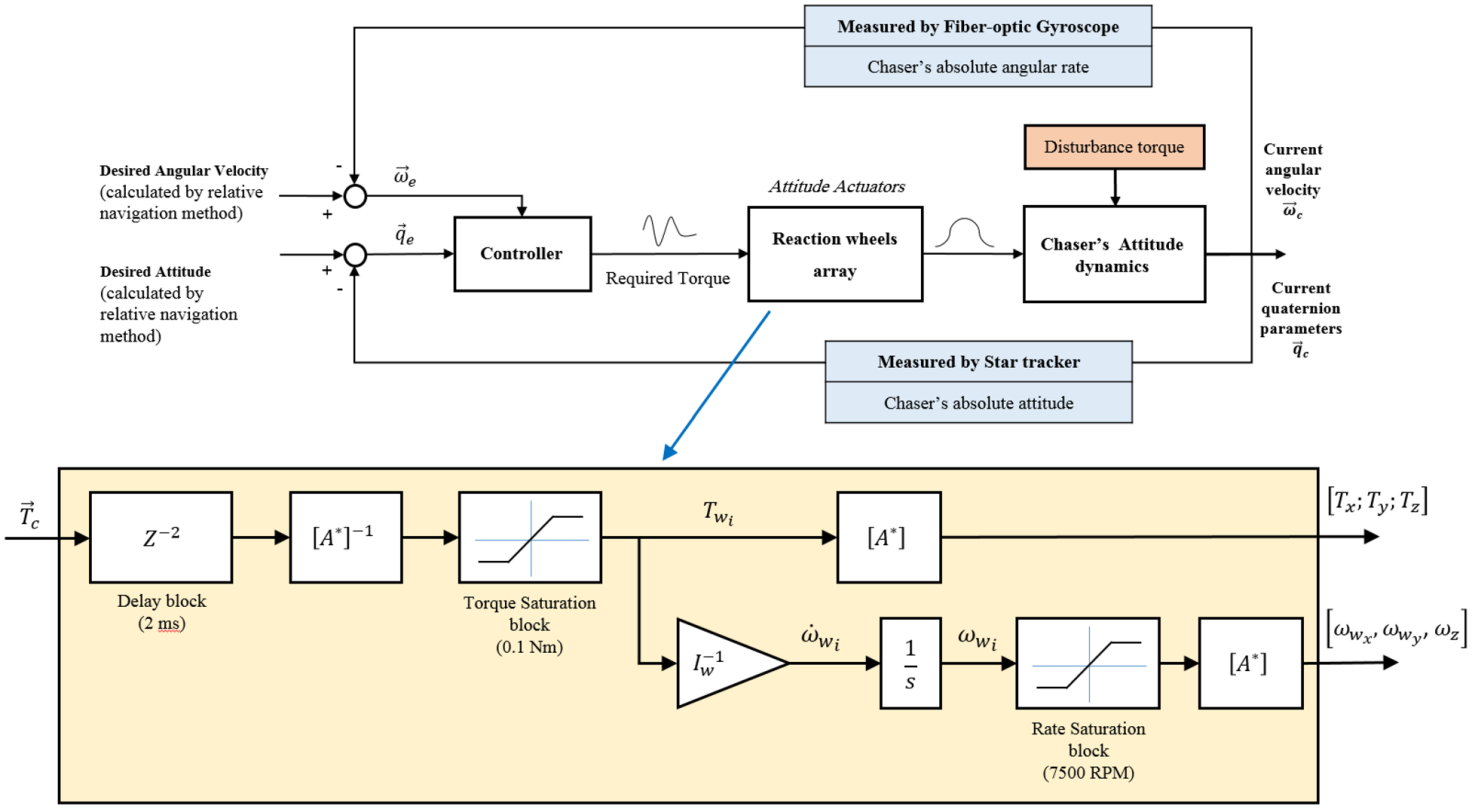
Block diagram of reaction wheel modeling.

In a simple overview, reaction wheels can be modeled by saturation (both for torque and angular momentum) and delay block in MATLAB-Simulink as shown in Fig. 6:

In Fig. 6, ${I}_{w}$, ${\omega }_{{w}_{i}}$ and ${\dot{\omega }}_{{w}_{i}}$ are the moment of inertia, angular velocity, and angular acceleration of the wheels.

**Figure 7:**
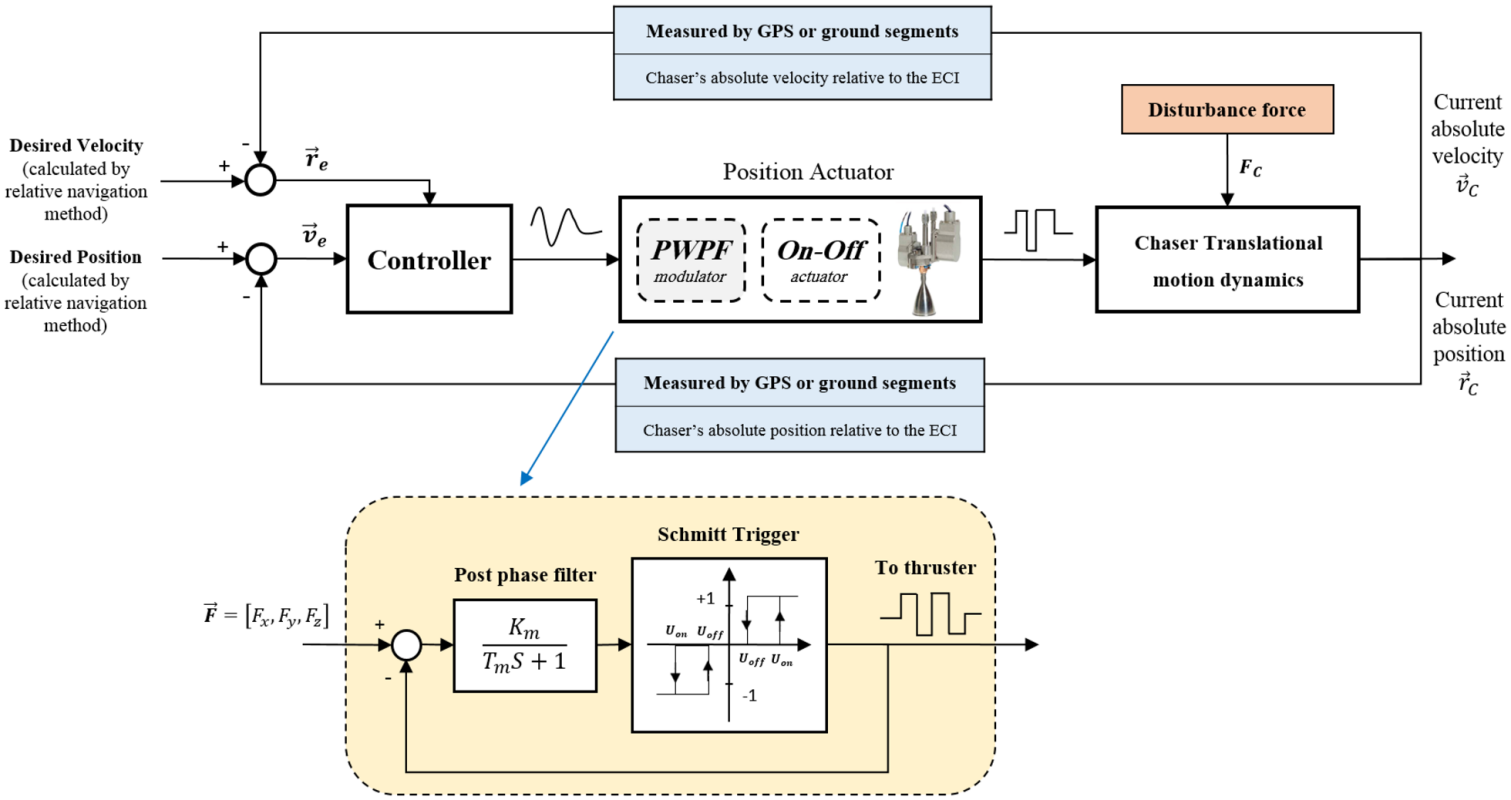
Block diagram of reaction thruster modeling.

In the case of impulse controllers, the relation between the controller's input and output is non-linear. This feature arises due to the On–Off nature of thrusters, too. Such a non-linear relation can be modeled using the pulse modulation method in a quasi-linear way. Among the different types of pulse modulation, the PWPF type is more popular due to its features such as near-linear performance, high accuracy, and the ability to adjust the width and frequency of the pulse. As can be seen in Fig. 7, the PWPF modulator simple model consists of a Schmitt trigger section and a post-phase filter. Considering the trust level to be fixed, this loop has four unknown parameters that must be adjusted to achieve the desired response^48^.

In Fig. 7, ${K}_{m}$ is pre-filter gain and ${T}_{m}$ is the time-constant. Also, ${U}_{on}$ and ${U}_{off}$ are known as the activation and deactivation values of Schmitt trigger, respectively. These parameters are inherent characteristics of the thruster, which are also affected by technical and technological limitations. But in general, these parameters can be optimized in the thruster design stage^48^.

## Controller design

In this section the controller law is designed for both (i) the relative attitude motion and (ii) the relative translational motion, on the basis of Eqs. (29) and (30).

### Relative attitude motion control

Consider the following format for a nonlinear plant:

48 $$\ddot{X}+{a}_{1}{f}_{{x}_{1}}+{a}_{2}{f}_{{x}_{2}}+\dots +{a}_{n}{f}_{{x}_{n}}=bu+d$$

where ${a}_{i}$ are unknown constant or slowly time-varying parameters, ${f}_{{x}_{i}}$ are known nonlinear or linear functions (including states or any measurable variable). $b$ is the control gain and $d$ denotes the external disturbance torque that is unknown but bounded.

From Eq. (28) and (29), ${\dot{\overrightarrow{\omega }}}_{e}$ can be expanded as follows:

49-a $$\begin{aligned} & {\dot{\omega }}_{{e}_{y}}+{I}_{{c}_{y}}^{-1}\left({I}_{{c}_{x}}-{I}_{{c}_{z}}\right){\omega }_{{c}_{x}}{\omega }_{{c}_{z}}+{I}_{{c}_{y}}^{-1}\left({h}_{{rw}_{x}}{\omega }_{{c}_{z}}-{h}_{{rw}_{z}}{\omega }_{{c}_{x}}\right)+\left({\omega }_{{c}_{z}}{\omega }_{{e}_{x}}-{\omega }_{{c}_{x}}{\omega }_{{e}_{z}}\right)\\ & \quad -\left[{c}_{21}{I}_{{t}_{x}}^{-1}\left({I}_{{t}_{z}}-{I}_{{t}_{y}}\right){\delta }_{2}{\delta }_{3}+{c}_{22}{I}_{{t}_{y}}^{-1}\left({I}_{{t}_{x}}-{I}_{{t}_{z}}\right){\delta }_{1}{\delta }_{3}+{c}_{23}{I}_{{t}_{z}}^{-1}\left({I}_{{t}_{y}}-{I}_{{t}_{x}}\right){\delta }_{1}{\delta }_{2}\right]\\ & \quad ={I}_{{t}_{y}}^{-1}{T}_{{c}_{y}}+{I}_{{c}_{y}}^{-1}{M}_{{tot}_{y}}-\left({c}_{21}{M}_{{tot}_{{t}_{x}}}+{c}_{22}{M}_{{tot}_{{t}_{y}}}+{c}_{23}{M}_{{tot}_{{t}_{z}}}\right){I}_{{t}_{y}}^{-1}\end{aligned}$$

49-b $${\dot{\omega }}_{{e}_{y}}+{I}_{{c}_{y}}^{-1}\left({I}_{{c}_{x}}-{I}_{{c}_{z}}\right){\omega }_{{c}_{x}}{\omega }_{{c}_{z}}+{I}_{{c}_{y}}^{-1}\left({h}_{{rw}_{x}}{\omega }_{{c}_{z}}-{h}_{{rw}_{z}}{\omega }_{{c}_{x}}\right)+\left({\omega }_{{c}_{z}}{\omega }_{{e}_{x}}-{\omega }_{{c}_{x}}{\omega }_{{e}_{z}}\right)-\left[{c}_{21}{I}_{{t}_{x}}^{-1}\left({I}_{{t}_{z}}-{I}_{{t}_{y}}\right){\delta }_{2}{\delta }_{3}+{c}_{22}{I}_{{t}_{y}}^{-1}\left({I}_{{t}_{x}}-{I}_{{t}_{z}}\right){\delta }_{1}{\delta }_{3}+{c}_{23}{I}_{{t}_{z}}^{-1}\left({I}_{{t}_{y}}-{I}_{{t}_{x}}\right){\delta }_{1}{\delta }_{2}\right]={I}_{{t}_{y}}^{-1}{T}_{{c}_{y}}+{I}_{{c}_{y}}^{-1}{M}_{{tot}_{y}}-\left({c}_{21}{M}_{{tot}_{{t}_{x}}}+{c}_{22}{M}_{{tot}_{{t}_{y}}}+{c}_{23}{M}_{{tot}_{{t}_{z}}}\right){I}_{{t}_{y}}^{-1}$$

49-c $$\begin{aligned} & {\dot{\omega }}_{{e}_{z}}+{I}_{{c}_{z}}^{-1}\left({I}_{{c}_{y}}-{I}_{{c}_{x}}\right){\omega }_{{c}_{x}}{\omega }_{{c}_{y}}+{I}_{{c}_{z}}^{-1}\left({h}_{{rw}_{y}}{\omega }_{{c}_{x}}-{h}_{{rw}_{x}}{\omega }_{{c}_{y}}\right)+\left({\omega }_{{c}_{x}}{\omega }_{{e}_{y}}-{\omega }_{{c}_{y}}{\omega }_{{e}_{x}}\right)\\ &\quad-\left[{c}_{31}{I}_{{t}_{x}}^{-1}\left({I}_{{t}_{z}}-{I}_{{t}_{y}}\right){\delta }_{2}{\delta }_{3}+{c}_{32}{I}_{{t}_{y}}^{-1}\left({I}_{{t}_{x}}-{I}_{{t}_{z}}\right){\delta }_{1}{\delta }_{3}+{c}_{33}{I}_{{t}_{z}}^{-1}\left({I}_{{t}_{y}}-{I}_{{t}_{x}}\right){\delta }_{1}{\delta }_{2}\right]\\ &\quad={I}_{{c}_{z}}^{-1}{T}_{{c}_{z}}+{I}_{{c}_{z}}^{-1}{M}_{{tot}_{z}}-\left({c}_{31}{M}_{{tot}_{{t}_{x}}}+{c}_{32}{M}_{{tot}_{{t}_{y}}}+{c}_{33}{M}_{{tot}_{{t}_{z}}}\right){I}_{{t}_{z}}^{-1}\end{aligned}$$

where $\overrightarrow{{\varvec{\updelta}}}={\left[{\delta }_{1},{\delta }_{2},{\delta }_{3}\right]}^{T}\epsilon {\mathfrak{R}}^{3\times 1}$ can be calculated as follows:

50 $$\left\{\begin{array}{c}{\delta }_{1}={ c}_{11}*({\omega }_{{c}_{x}} - {\omega }_{{e}_{x}}) + {c}_{12}*({\omega }_{{c}_{y}} - {\omega }_{{e}_{y}}) + {c}_{13}*({\omega }_{{c}_{z}} - {\omega }_{{e}_{z}})\\ {\delta }_{2}={ c}_{21}*({\omega }_{{c}_{x}} - {\omega }_{{e}_{x}}) + {c}_{22}*({\omega }_{{c}_{y}} - {\omega }_{{e}_{y}}) + {c}_{23}*({\omega }_{{c}_{z}} - {\omega }_{{e}_{z}})\\ {\delta }_{3}={ c}_{31}*({\omega }_{{c}_{x}} - {\omega }_{{e}_{x}}) + {c}_{32}*({\omega }_{{c}_{y}} - {\omega }_{{e}_{y}}) + {c}_{33}*({\omega }_{{c}_{z}} - {\omega }_{{e}_{z}})\end{array}\right.$$

where ${c}_{ij}, i and j=\text{1,2},3$ are the entry of matrix ${C}_{t}^{{c}^{-1}}$.

The relative attitude motion equations (Eqs. (49-a) to (49-c)) can be rewritten in the form of Eq. (48), where ${a}_{n}, b$ and ${f}_{{x}_{n}}$ are defined in Table 1:

**Table 1:** Variable change.

X axis	Y axis	Z axis
${b}_{x}={I}_{{c}_{x}}^{-1}$	${b}_{y}={I}_{{c}_{y}}^{-1}$	${b}_{z}={I}_{{c}_{z}}^{-1}$
$$\left\{\begin{array}{c}{a}_{{x}_{1}}=1\\ {f}_{{x}_{1}}={\omega }_{{c}_{y}}{\omega }_{{e}_{z}}-{\omega }_{{c}_{z}}{\omega }_{{e}_{y}}\end{array}\right.$$	$$\left\{\begin{array}{c}{a}_{{y}_{1}}=1\\ {f}_{{y}_{1}}={\omega }_{{c}_{z}}{\omega }_{{e}_{x}}-{\omega }_{{c}_{x}}{\omega }_{{e}_{z}}\end{array}\right.$$	$$\left\{\begin{array}{c}{a}_{{z}_{1}}=1\\ {f}_{{z}_{1}}={\omega }_{{c}_{x}}{\omega }_{{e}_{y}}-{\omega }_{{c}_{y}}{\omega }_{{e}_{x}}\end{array}\right.$$
$$\left\{\begin{array}{c}{a}_{{x}_{2}}={I}_{{c}_{x}}^{-1}\left({I}_{{c}_{z}}-{I}_{{c}_{y}}\right)\\ {f}_{{x}_{2}}={\omega }_{{c}_{y}}{\omega }_{{c}_{z}}\end{array}\right.$$	$$\left\{\begin{array}{c}{a}_{{y}_{2}}={I}_{{c}_{y}}^{-1}\left({I}_{{c}_{x}}-{I}_{{c}_{z}}\right)\\ {f}_{{y}_{2}}={\omega }_{{c}_{x}}{\omega }_{{c}_{z}}\end{array}\right.$$	$$\left\{\begin{array}{c}{a}_{{z}_{2}}={I}_{{c}_{z}}^{-1}\left({I}_{{c}_{y}}-{I}_{{c}_{x}}\right)\\ {f}_{{z}_{2}}={\omega }_{{c}_{x}}{\omega }_{{c}_{y}}\end{array}\right.$$
$$\left\{\begin{array}{c}{a}_{{x}_{3}}={I}_{{c}_{x}}^{-1}\\ {f}_{{x}_{3}}={h}_{{rw}_{z}}{\omega }_{{c}_{y}}-{h}_{{rw}_{y}}{\omega }_{{c}_{z}}\end{array}\right.$$	$$\left\{\begin{array}{c}{a}_{{y}_{3}}={I}_{{c}_{y}}^{-1}\\ {f}_{{y}_{3}}={h}_{{rw}_{x}}{\omega }_{{c}_{z}}-{h}_{{rw}_{z}}{\omega }_{{c}_{x}}\end{array}\right.$$	$$\left\{\begin{array}{c}{a}_{{z}_{3}}={I}_{{c}_{z}}^{-1}\\ {f}_{{z}_{3}}={h}_{{rw}_{y}}{\omega }_{{c}_{x}}-{h}_{{rw}_{x}}{\omega }_{{c}_{y}}\end{array}\right.$$
$$\left\{\begin{array}{c}{a}_{{x}_{4}}=-{I}_{{t}_{x}}^{-1}\left({I}_{{t}_{z}}-{I}_{{t}_{y}}\right)\\ {f}_{{x}_{4}}={c}_{11}{\delta }_{2}{\delta }_{3}\end{array}\right.$$	$$\left\{\begin{array}{c}{a}_{{y}_{4}}=-{I}_{{t}_{x}}^{-1}\left({I}_{{t}_{z}}-{I}_{{t}_{y}}\right)\\ {f}_{{y}_{4}}={c}_{21}{\delta }_{2}{\delta }_{3}\end{array}\right.$$	$$\left\{\begin{array}{c}{a}_{{z}_{4}}=-{I}_{{t}_{x}}^{-1}\left({I}_{{t}_{z}}-{I}_{{t}_{y}}\right)\\ {f}_{{z}_{4}}={c}_{31}{\delta }_{2}{\delta }_{3}\end{array}\right.$$
$$\left\{\begin{array}{c}{a}_{{x}_{5}}=-{I}_{{t}_{y}}^{-1}\left({I}_{{t}_{x}}-{I}_{{t}_{z}}\right)\\ {f}_{{x}_{5}}={c}_{12}{\delta }_{1}{\delta }_{3}\end{array}\right.$$	$$\left\{\begin{array}{c}{a}_{{y}_{5}}=-{I}_{{t}_{y}}^{-1}\left({I}_{{t}_{x}}-{I}_{{t}_{z}}\right)\\ {f}_{{y}_{5}}={c}_{22}{\delta }_{1}{\delta }_{3}\end{array}\right.$$	$$\left\{\begin{array}{c}{a}_{{z}_{5}}=-{I}_{{t}_{y}}^{-1}\left({I}_{{t}_{x}}-{I}_{{t}_{z}}\right)\\ {f}_{{z}_{5}}={c}_{32}{\delta }_{1}{\delta }_{3}\end{array}\right.$$
$$\left\{\begin{array}{c}{a}_{{x}_{6}}=-{I}_{{t}_{z}}^{-1}\left({I}_{y}-{I}_{{t}_{x}}\right)\\ {f}_{{x}_{6}}={c}_{13}{\delta }_{1}{\delta }_{2}\end{array}\right.$$	$$\left\{\begin{array}{c}{a}_{{y}_{6}}=-{I}_{{t}_{z}}^{-1}\left({I}_{{t}_{y}}-{I}_{{t}_{x}}\right)\\ {f}_{{y}_{6}}={c}_{23}{\delta }_{1}{\delta }_{2}\end{array}\right.$$	$$\left\{\begin{array}{c}{a}_{{z}_{6}}=-{I}_{{t}_{z}}^{-1}\left({I}_{{t}_{y}}-{I}_{{t}_{x}}\right)\\ {f}_{{z}_{6}}={c}_{33}{\delta }_{1}{\delta }_{2}\end{array}\right.$$

In this section, only the controller design process for X-axis will be mentioned, then we will generalize the results for the other two axes.

Note that ${a}_{{x}_{1}}=1$ is known and can be canceled by the feedback linearization method. Thus, we don't have to estimate it.

All the disturbance terms $\left({I}_{{c}_{x}}^{-1}{M}_{{tot}_{x}}-\left({c}_{31}{M}_{{tot}_{{t}_{x}}}+{c}_{32}{M}_{{tot}_{{t}_{y}}}+{c}_{33}{M}_{{tot}_{{t}_{z}}}\right){I}_{{t}_{x}}^{-1}\right)$ are assumed to be bounded and follow the following relationship:

51 $$\left|{I}_{{c}_{x}}^{-1}{M}_{{tot}_{x}}-\left({c}_{31}{M}_{{tot}_{{t}_{x}}}+{c}_{32}{M}_{{tot}_{{t}_{y}}}+{c}_{33}{M}_{{tot}_{{t}_{z}}}\right){I}_{{t}_{x}}^{-1}\right|\le {D}_{x}$$

First of all, the sliding surface is given as:

52 $${S}_{{aug}_{x}}={s}_{x}-{d}_{x}sign({s}_{x})$$

where ${d}_{x}$ is a constant bounded gain that can be considered as the bandwidth or manifold of the discontinuous control part. This term also gives more flexibility to the designer to set the time responses of the satellite and the steady-state error.

$${s}_{x}=\left(\frac{d}{dt}+{\lambda }_{0}\right){e}_{x}$$

53 $${s}_{x}={\dot{e}}_{x}+{\lambda }_{0}{e}_{x}$$

where the position error along x-axis (${e}_{x}$) is defined as:

54 $${e}_{x}={q}_{1}-{q}_{{1}_{d}}$$

The controller signal is:

55 $${u}_{{2}_{x}}=-{\widehat{b}}_{x}\left[{u}_{{1}_{x}}+\left({D}_{x}+{\varepsilon }_{x}\right)sign\left({s}_{x}\right)\right]+{a}_{{x}_{1}}{f}_{{x}_{1}}+\sum_{2}^{6}{\widehat{a}}_{{x}_{i}}{f}_{{x}_{i}}$$

where ${u}_{{1}_{x}}$ is defined as follows:

56 $${u}_{{1}_{x}}=-{\dot{\omega }}_{{e}_{{x}_{d}}}+{\lambda }_{0}{\dot{e}}_{x}$$

${\widehat{a}}_{{x}_{i}}$ and ${\widehat{b}}_{x}$ will be estimated online according to adaptation law/rule yet to be defined. ${\lambda }_{0}$ and ${\varepsilon }_{x}$ are positive constants. ${\dot{\omega }}_{{e}_{{x}_{d}}}$ denotes the desired relative angular acceleration between the chaser and target spacecraft.

To find the adaptation law that guarantees stability simultaneously, we have to propose a Lyapunov candidate as follows:

57 $${V}_{x}=\frac{1}{2}{S}_{{aug}_{x}}^{2}+\frac{1}{2}{b}_{x}\left[\sum_{i=2}^{6}{\left({\widehat{a}}_{{x}_{i}}-\frac{{a}_{{x}_{i}}}{{b}_{x}}\right)}^{2}+{\left({\widehat{b}}_{x}-{b}_{x}^{-1}\right)}^{2}\right]$$

Note that outside of the boundary layer ${\dot{S}}_{{aug}_{x}}$ is equal to ${\dot{s}}_{x}$, while inside the boundary layer ${S}_{{aug}_{x}}=0$ and we have for the time derivative of the Lyapunov function:

58 $${\dot{V}}_{x}={S}_{{aug}_{x}}{\dot{S}}_{{aug}_{x}}+{b}_{x}\left[\sum_{i=2}^{6}{\dot{\widehat{a}}}_{{x}_{i}}\left({\widehat{a}}_{{x}_{i}}-\frac{{a}_{{x}_{i}}}{{b}_{x}}\right)+{\dot{\widehat{b}}}_{x}\left({\widehat{b}}_{x}-{b}_{x}^{-1}\right)\right]$$

59 $${\dot{V}}_{x}={S}_{{aug}_{x}}\left({\ddot{e}}_{x}+{\lambda }_{0}{\dot{e}}_{x}\right)+{b}_{x}\left[\sum_{i=2}^{6}{\dot{\widehat{a}}}_{{x}_{i}}\left({\widehat{a}}_{{x}_{i}}-\frac{{a}_{{x}_{i}}}{{b}_{x}}\right)+{\dot{\widehat{b}}}_{x}\left({\widehat{b}}_{x}-{b}_{x}^{-1}\right)\right]$$

60 $${\dot{V}}_{x}={S}_{{aug}_{x}}{\dot{\omega }}_{{e}_{x}}+{S}_{{aug}_{x}}\left(-{\dot{\omega }}_{{e}_{{x}_{d}}}+{\lambda }_{0}{\dot{e}}_{x}\right)+{b}_{x}\sum_{i=2}^{6}{\dot{\widehat{a}}}_{{x}_{i}}\left({\widehat{a}}_{{x}_{i}}-\frac{{a}_{{x}_{i}}}{{b}_{x}}\right)+{b}_{x}{\dot{\widehat{b}}}_{x}\left({\widehat{b}}_{x}-{b}_{x}^{-1}\right)$$

Substituting ${\dot{\omega }}_{{e}_{x}}$ from Eq. (49-a) into Eq. (60)

61 $$\begin{aligned}{\dot{V}}_{x}&={S}_{{aug}_{x}}\left(-{I}_{{c}_{x}}^{-1}\left({I}_{{c}_{z}}-{I}_{{c}_{y}}\right){\omega }_{{c}_{y}}{\omega }_{{c}_{z}}-{I}_{{c}_{x}}^{-1}\left({h}_{{rw}_{z}}{\omega }_{{c}_{y}}-{h}_{{rw}_{y}}{\omega }_{{c}_{z}}\right)-\left({\omega }_{{c}_{y}}{\omega }_{{e}_{z}}-{\omega }_{{c}_{z}}{\omega }_{{e}_{y}}\right)\right. \\ & \quad \left.+\left[{c}_{11}{I}_{{t}_{x}}^{-1}\left({I}_{{t}_{z}}-{I}_{{t}_{y}}\right){\delta }_{2}{\delta }_{3}+{c}_{12}{I}_{{t}_{y}}^{-1}\left({I}_{{t}_{x}}-{I}_{{t}_{z}}\right){\delta }_{1}{\delta }_{3}+{c}_{13}{I}_{{t}_{z}}^{-1}\left({I}_{{t}_{y}}-{I}_{{t}_{x}}\right){\delta }_{1}{\delta }_{2}\right]\right. \\ & \quad \left.+{I}_{{c}_{x}}^{-1}{\overrightarrow{M}}_{tot}-{I}_{t}^{-1}{C}_{t}^{c}{\overrightarrow{M}}_{{tot}_{t}}+{I}_{{c}_{x}}^{-1}{\overrightarrow{T}}_{c}\right)+{S}_{{aug}_{x}}\left(-{\dot{\omega }}_{{e}_{{x}_{d}}}+{\lambda }_{0}{\dot{e}}_{x}\right)+{b}_{x}\sum_{i=2}^{6}{\dot{\widehat{a}}}_{{x}_{i}}\left({\widehat{a}}_{{x}_{i}}-\frac{{a}_{{x}_{i}}}{{b}_{x}}\right)+{\dot{\widehat{b}}}_{x}\left({b}_{x}{\widehat{b}}_{x}-1\right)\end{aligned}$$

Now substituting ${u}_{{2}_{x}}={\overrightarrow{T}}_{c}$ from Eq. (55) into Eq. (61) gives:

62 $$\begin{aligned}{\dot{V}}_{x}& ={S}_{{aug}_{x}}\left(-{I}_{{c}_{x}}^{-1}\left({I}_{{c}_{z}}-{I}_{{c}_{y}}\right){\omega }_{{c}_{y}}{\omega }_{{c}_{z}}-{I}_{{c}_{x}}^{-1}\left({h}_{{rw}_{z}}{\omega }_{{c}_{y}}-{h}_{{rw}_{y}}{\omega }_{{c}_{z}}\right)-\left({\omega }_{{c}_{y}}{\omega }_{{e}_{z}}-{\omega }_{{c}_{z}}{\omega }_{{e}_{y}}\right)\right. \\ & \quad \left. +\left[{c}_{11}{I}_{{t}_{x}}^{-1}\left({I}_{{t}_{z}}-{I}_{{t}_{y}}\right){\delta }_{2}{\delta }_{3}+{c}_{12}{I}_{{t}_{y}}^{-1}\left({I}_{{t}_{x}}-{I}_{{t}_{z}}\right){\delta }_{1}{\delta }_{3}+{c}_{13}{I}_{{t}_{z}}^{-1}\left({I}_{{t}_{y}}-{I}_{{t}_{x}}\right){\delta }_{1}{\delta }_{2}\right]\right. \\ & \quad \left. +{I}_{{c}_{x}}^{-1}{\overrightarrow{M}}_{tot}-{I}_{t}^{-1}{C}_{t}^{c}{\overrightarrow{M}}_{{tot}_{t}}+{I}_{{c}_{x}}^{-1}\left(-{\widehat{b}}_{x}\left[{u}_{{1}_{x}}+\left({D}_{x}+{\varepsilon }_{x}\right)sign\left({s}_{x}\right)\right]+\sum_{1}^{6}{\widehat{a}}_{{x}_{i}}{f}_{{x}_{i}}\right)\right)\\ & \quad +{S}_{{aug}_{x}}\left(-{\dot{\omega }}_{{e}_{{x}_{d}}}+{\lambda }_{0}{\dot{e}}_{x}\right)+{b}_{x}\sum_{i=2}^{6}{\dot{\widehat{a}}}_{{x}_{i}}\left({\widehat{a}}_{{x}_{i}}-\frac{{a}_{{x}_{i}}}{{b}_{x}}\right)+{\dot{\widehat{b}}}_{x}\left({b}_{x}{\widehat{b}}_{x}-1\right)\end{aligned}$$

For simplicity, using the first column of Table 1, Eq. (62) can be rewritten as follows:

63 $$\begin{aligned}{\dot{V}}_{x} & = {S}_{{aug}_{x}}\left(-\sum_{i=1}^{6}{a}_{{x}_{i}}{f}_{{x}_{i}}+{I}_{{c}_{x}}^{-1}{\overrightarrow{M}}_{tot}-{I}_{t}^{-1}{C}_{t}^{c}{\overrightarrow{M}}_{{tot}_{t}}+{b}_{x}\left(-{\widehat{b}}_{x}\left[{u}_{{1}_{x}}+\left({D}_{x}+{\varepsilon }_{x}\right)sign\left({s}_{x}\right)\right]+\sum_{1}^{6}{\widehat{a}}_{{x}_{i}}{f}_{{x}_{i}}\right)\right)\\ & \quad +{S}_{{aug}_{x}}\left(-{\dot{\omega }}_{{e}_{{x}_{d}}}+{\lambda }_{0}{\dot{e}}_{x}\right)+{b}_{x}\sum_{i=2}^{6}{\dot{\widehat{a}}}_{{x}_{i}}\left({\widehat{a}}_{{x}_{i}}-\frac{{a}_{{x}_{i}}}{{b}_{x}}\right)+{\dot{\widehat{b}}}_{x}\left({b}_{x}{\widehat{b}}_{x}-1\right)\end{aligned}$$

By simplifying and sorting the above equation, we will have:

64 $$\begin{aligned}{\dot{V}}_{x} & = \left({b}_{x}{\widehat{a}}_{{x}_{2}}-{a}_{{x}_{2}}\right)\left({f}_{{x}_{2}}{S}_{{aug}_{x}}+{\dot{\widehat{a}}}_{{x}_{2}}\right)+\left({b}_{x}{\widehat{a}}_{{x}_{3}}-{a}_{{x}_{3}}\right)\left({f}_{{x}_{3}}{S}_{{aug}_{x}}+{\dot{\widehat{a}}}_{{x}_{3}}\right)+\left({b}_{x}{\widehat{a}}_{{x}_{4}}-{a}_{{x}_{4}}\right)\left({f}_{{x}_{4}}{S}_{{aug}_{x}}+{\dot{\widehat{a}}}_{{x}_{4}}\right)\\ &\quad+\left({b}_{x}{\widehat{a}}_{{x}_{5}}-{a}_{{x}_{5}}\right)\left({f}_{{x}_{5}}{S}_{{aug}_{x}}+{\dot{\widehat{a}}}_{{x}_{5}}\right)+\left({b}_{x}{\widehat{a}}_{{x}_{6}}-{a}_{{x}_{6}}\right)\left({f}_{{x}_{6}}{S}_{{aug}_{x}}+{\dot{\widehat{a}}}_{{x}_{6}}\right)+\left(1-{b}_{x}{\widehat{b}}_{x}\right){u}_{{1}_{x}}{S}_{{aug}_{x}}\\ &\quad-{b}_{x}{\widehat{b}}_{x}\left({D}_{x}+{\varepsilon }_{x}\right)sign\left({s}_{x}\right){S}_{{aug}_{x}}+\left({I}_{{c}_{x}}^{-1}{\overrightarrow{M}}_{tot}-{I}_{t}^{-1}{C}_{t}^{c}{\overrightarrow{M}}_{{tot}_{t}}\right){S}_{{aug}_{x}}-{\dot{\widehat{b}}}_{x}\left(1-{b}_{x}{\widehat{b}}_{x}\right)\end{aligned}$$

Equation (65) represents the simplified model of Eq. (64).

65 $$\begin{aligned}{\dot{V}}_{x}& = \sum_{i=2}^{6}\left({b}_{x}{\widehat{a}}_{{x}_{i}}-{a}_{{x}_{i}}\right)\left({f}_{{x}_{i}}{S}_{{aug}_{x}}+{\dot{\widehat{a}}}_{{x}_{i}}\right)+\left(1-{b}_{x}{\widehat{b}}_{x}\right){u}_{{1}_{x}}{S}_{{aug}_{x}}-{b}_{x}{\widehat{b}}_{x}\left({D}_{x}+{\varepsilon }_{x}\right)sign\left({s}_{x}\right){S}_{{aug}_{x}}\\ & \quad +\left({I}_{{c}_{x}}^{-1}{\overrightarrow{M}}_{tot}-{I}_{t}^{-1}{C}_{t}^{C}{\overrightarrow{M}}_{{tot}_{t}}\right){S}_{{aug}_{x}}-{\dot{\widehat{b}}}_{x}\left(1-{b}_{x}{\widehat{b}}_{x}\right)\end{aligned}$$

Selecting the adaptation law as follows can guarantee ${\dot{V}}_{x}\le 0$

66 $${\dot{\widehat{a}}}_{{x}_{i}}=-{f}_{{x}_{\text{i}}}{S}_{{aug}_{x}}, i=\text{2,3},\dots ,6$$

67 $${\dot{\widehat{b}}}_{x}={u}_{{1}_{x}}{S}_{{aug}_{x}}+\left({D}_{x}+{\varepsilon }_{x}\right)sign\left({s}_{x}\right){S}_{{aug}_{x}}$$

Then, substituting ${\dot{\widehat{a}}}_{{x}_{i}}$ and ${\dot{\widehat{b}}}_{x}$ into Eq. (65) leads to:

68 $${\dot{V}}_{x}=-\left({D}_{x}+{\varepsilon }_{x}\right)sign\left({s}_{x}\right){S}_{{aug}_{x}}+\left({I}_{{c}_{x}}^{-1}{\overrightarrow{M}}_{tot}-{I}_{t}^{-1}{C}_{t}^{C}{\overrightarrow{M}}_{{tot}_{t}}\right){S}_{{aug}_{x}}\le -{\varepsilon }_{x}\left|{S}_{{aug}_{x}}\right|$$

The upper bound of ${I}_{{c}_{x}}^{-1}{\overrightarrow{M}}_{tot}-{I}_{t}^{-1}{C}_{t}^{C}{\overrightarrow{M}}_{{tot}_{t}}$ is shown as ${D}^{*}$.

69 $${\dot{V}}_{x}=\left[-\left({D}_{x}+{\varepsilon }_{x}\right)sign\left({s}_{x}\right)+{D}^{*}\right]{S}_{{aug}_{x}}\le -{\varepsilon }_{x}\left|{S}_{{aug}_{x}}\right|$$

Moreover, Eq. (70) is a correct algebraic fact:

70 $$-\left({D}_{x}+{\varepsilon }_{x}\right)\left|{S}_{{aug}_{x}}\right|+{D}^{*}{S}_{{aug}_{x}}\le -\left({D}_{x}+{\varepsilon }_{x}\right)\left|{S}_{{aug}_{x}}\right|+{D}^{*}\left|{S}_{{aug}_{x}}\right|$$

The relation that results from combining Eqs. (69) and (70) is as follows:

71 $$-\left({D}_{x}+{\varepsilon }_{x}\right)\left|{S}_{{aug}_{x}}\right|+{D}^{*}\left|{S}_{{aug}_{x}}\right|\le -{\varepsilon }_{x}\left|{S}_{{aug}_{x}}\right|$$

72 $$-\left({D}_{x}+{\varepsilon }_{x}\right)+{D}^{*}\le -{\varepsilon }_{x}$$

As an extra explanation, inequality of Eq. (70) is definitely correct because as mentioned before in Eq. (51) we have ${D}_{x}\ge {D}^{*}$. In other word, when it proved that $-\left({D}_{x}+{\varepsilon }_{x}\right)\left|{S}_{{aug}_{x}}\right|+{D}^{*}\left|{S}_{{aug}_{x}}\right|$ is less than $-{\varepsilon }_{x}\left|{S}_{{aug}_{x}}\right|$, it can be claimed that $\left[-\left({D}_{x}+{\varepsilon }_{x}\right)sign\left({s}_{x}\right)+{D}^{*}\right]{S}_{{aug}_{x}}$ is definitely less than $-{\varepsilon }_{x}\left|{S}_{{aug}_{x}}\right|$. Thus, Eq. (69) is established.

Thus ${\dot{V}}_{x}\le 0$, it means that the Lyapunov candidate was positive definite and its time-derivative is negative definite.

Without loss of generality and similar to what is said for the X-axis, the control signal for Y and Z axis will be equal to:

73 $${u}_{{2}_{y}}=-{\widehat{b}}_{y}\left[{u}_{{1}_{y}}+\left({D}_{y}+{\varepsilon }_{y}\right)sign\left({s}_{y}\right)\right]+{a}_{{y}_{1}}{f}_{{y}_{1}}+\sum_{2}^{6}{\widehat{a}}_{{y}_{i}}{f}_{{y}_{i}}$$

74 $${u}_{{2}_{z}}=-{\widehat{b}}_{z}\left[{u}_{{1}_{z}}+\left({D}_{z}+{\varepsilon }_{z}\right)sign\left({s}_{z}\right)\right]+{a}_{{z}_{1}}{f}_{{z}_{1}}+\sum_{2}^{6}{\widehat{a}}_{{z}_{i}}{f}_{{z}_{i}}$$

Also, the parameters adaptation laws are:

75 $${\dot{\widehat{a}}}_{{y}_{i}}=-{f}_{{y}_{i}}{S}_{{aug}_{y}}, i=\text{2,3},\dots ,6$$

76 $${\dot{\widehat{b}}}_{y}={u}_{{1}_{y}}{S}_{{aug}_{y}}+\left({D}_{y}+{\varepsilon }_{y}\right)sign\left({s}_{y}\right){S}_{{aug}_{y}}$$

77 $${\dot{\widehat{a}}}_{{z}_{i}}=-{f}_{{z}_{i}}{S}_{{aug}_{z}}, i=\text{2,3},\dots ,6$$

78 $${\dot{\widehat{b}}}_{z}={u}_{{1}_{z}}{S}_{{aug}_{z}}+\left({D}_{z}+{\varepsilon }_{z}\right)sign\left({s}_{z}\right){S}_{{aug}_{z}}$$

And sliding surfaces will be as follows:

79 $${S}_{{aug}_{y}}={\dot{e}}_{y}+{\lambda }_{0}{e}_{y}-{d}_{y}sign({s}_{y})$$

80 $${S}_{{aug}_{z}}={\dot{e}}_{z}+{\lambda }_{0}{e}_{z}-{d}_{z}sign({s}_{z})$$

### Relative translational motion control

As we mentioned previously, the target is assumed to be non-cooperative and its absolute position (${\overrightarrow{r}}_{t}$) or motion $\left({\overrightarrow{v}}_{t}, {\overrightarrow{\omega }}_{t}\right)$ data is not available. Replacing ${\overrightarrow{r}}_{t}$ with ${\overrightarrow{r}}_{c}-{\overrightarrow{r}}_{e}$ in Eq. (30), gives:

81 $$\ddot{x}=2{n}_{t}\dot{y}+{{n}_{t}}^{2}x-\frac{\mu }{{r}_{c}^{3}}x+{\dot{n}}_{t}y-\frac{\mu }{{r}_{c}^{2}}+\mu {f}_{{xx}_{2}}\left(x\right)+\frac{\mu }{{r}_{c}^{3}}{f}_{{xx}_{1}}\left(x\right)+{C}_{c}^{LVLH}\frac{1}{{m}_{C}}{F}_{{c}_{x}}+{a}_{{d}_{x}}$$

where $\frac{\mu }{{r}_{c}^{2}}$ is a time-varying but known parameter in every step time, ${f}_{{xx}_{1}}\left(x\right)$ and ${f}_{{xx}_{2}}\left(x\right)$ are nonlinear terms and defined as follows:

82 $${f}_{{xx}_{1}}\left(x\right)=\left(\sqrt{{x}^{2}+{y}^{2}+{z}^{2}}\right)$$

83 $${f}_{{xx}_{2}}\left(x\right)=\frac{1}{{\left({r}_{c}-\sqrt{{x}^{2}+{y}^{2}+{z}^{2}}\right)}^{2}}$$

For the sake of simplicity, we used ${g}_{x{x}_{1}}=\dot{y}, {g}_{x{x}_{2}}=x, {g}_{x{x}_{3}}=y$ and ${\alpha }_{x{x}_{1}}=2n, {\alpha }_{x{x}_{2}}={n}^{2}-\frac{\mu }{{r}_{C}^{3}}, {\alpha }_{x{x}_{3}}=\dot{n}$ and ${a}_{x{x}_{1}}=\frac{\mu }{{r}_{C}^{3}}$ , ${a}_{x{x}_{2}}=\mu$ transformations.

The sliding surface for X-axis is defined as:

84 $${S}_{{aug}_{xx}}={s}_{xx}-{d}_{xx}sign({s}_{xx})$$

85 $${s}_{xx}={\dot{e}}_{xx}+{\lambda }_{0}{e}_{xx}$$

The control signal (${u}_{{2}_{xx}}$) is given as:

86 $${u}_{{2}_{xx}}=-{\widehat{b}}^{-1}\left[{u}_{{1}_{xx}}+\left({D}_{xx}+{\varepsilon }_{xx}\right)sign\left({s}_{xx}\right)\right]-\sum_{{\alpha }_{{xx}_{i}}=1}^{3}{\widehat{\alpha }}_{{xx}_{i}}{g}_{x{x}_{i}}-\sum_{{a}_{{xx}_{i}}=1}^{2}{\widehat{a}}_{{xx}_{i}}{f}_{x{x}_{i}}\left(x\right)$$

where ${D}_{xx}$ is a positive constant that will be calculated in the next. $b$ and ${u}_{{1}_{xx}}$ are defined as:

87 $$b={C}_{c}^{LVLH}\frac{1}{{m}_{c}}$$

88 $${u}_{{1}_{xx}}={-\ddot{x}}_{d}+{\lambda }_{0}{\dot{e}}_{xx}$$

where ${\ddot{x}}_{d}$ is the desired acceleration along the x-axis.

The Lyapunov candidate function is selected as:

89 $${V}_{xx}=\frac{1}{2}{S}_{{aug}_{xx}}^{2}-\frac{1}{2}b\left[\sum_{i=1}^{3}{\left({\widehat{\alpha }}_{{xx}_{i}}-\frac{{\alpha }_{{xx}_{i}}}{b}\right)}^{2}+\sum_{i=1}^{2}{\left({\widehat{a}}_{{xx}_{i}}-\frac{{a}_{{xx}_{i}}}{b}\right)}^{2}-{\left(\widehat{h}-h\right)}^{2}\right]$$

where $$h=\frac{1}{b}={m}_{c}{C}_{c}^{{LVLH}^{-1}}$$

Time-derivative of ${V}_{xx}$ is given as follows:

90 $${\dot{V}}_{xx}={S}_{{aug}_{xx}}{\dot{S}}_{{aug}_{xx}}-b\left[\sum_{i=1}^{3}{\dot{\widehat{\alpha }}}_{{xx}_{i}}\left({\widehat{\alpha }}_{{xx}_{i}}-\frac{{\alpha }_{{xx}_{i}}}{b}\right)+\sum_{i=1}^{2}{\dot{\widehat{a}}}_{{xx}_{i}}\left({\widehat{a}}_{{xx}_{i}}-\frac{{a}_{{xx}_{i}}}{b}\right)-\dot{\widehat{h}}\left(\widehat{h}-h\right)\right]$$

91 $${\dot{V}}_{xx}={S}_{{aug}_{xx}}\left({\ddot{e}}_{xx}+{\lambda }_{{xx}_{0}}{\dot{e}}_{xx}\right)-b\left[\sum_{i=1}^{3}{\dot{\widehat{\alpha }}}_{{xx}_{i}}\left({\widehat{\alpha }}_{{xx}_{i}}-\frac{{\alpha }_{{xx}_{i}}}{b}\right)+\sum_{i=1}^{2}{\dot{\widehat{a}}}_{{xx}_{i}}\left({\widehat{a}}_{{xx}_{i}}-\frac{{a}_{{xx}_{i}}}{b}\right)-\dot{\widehat{h}}\left(\widehat{h}-h\right)\right]$$

92 $${\dot{V}}_{xx}={S}_{{aug}_{xx}}\left(\ddot{x}-{\ddot{x}}_{d}+{\lambda }_{{xx}_{0}}{\dot{e}}_{xx}\right)-b\left[\sum_{i=1}^{3}{\dot{\widehat{\alpha }}}_{{xx}_{i}}\left({\widehat{\alpha }}_{{xx}_{i}}-\frac{{\alpha }_{{xx}_{i}}}{b}\right)+\sum_{i=1}^{2}{\dot{\widehat{a}}}_{{xx}_{i}}\left({\widehat{a}}_{{xx}_{i}}-\frac{{a}_{{xx}_{i}}}{b}\right)-\dot{\widehat{h}}\left(\widehat{h}-h\right)\right]$$

Substituting Eq. (81) in Eq. (92) gives:

93 $$\begin{aligned}{\dot{V}}_{xx}& ={S}_{{aug}_{xx}}\left(2{n}_{t}\dot{y}+{n}_{t}^{2}x+{\dot{n}}_{t}y-\frac{\mu }{{r}_{c}^{2}}+\mu {f}_{{xx}_{2}}\left(x\right)+\frac{\mu }{{r}_{c}^{3}}{f}_{{xx}_{1}}\left(x\right)-\frac{\mu }{{r}_{c}^{3}}x+{C}_{c}^{LVLH}\frac{1}{{m}_{c}}{F}_{{c}_{x}}+{a}_{{d}_{x}}-{\ddot{x}}_{d}+{\lambda }_{0}{\dot{e}}_{x}\right)\\ & \quad-b\sum_{i=1}^{3}{\dot{\widehat{\alpha }}}_{{xx}_{i}}\left({\widehat{\alpha }}_{{xx}_{i}}-\frac{{\alpha }_{{xx}_{i}}}{b}\right)-b\sum_{i=1}^{2}{\dot{\widehat{a}}}_{{xx}_{i}}\left({\widehat{a}}_{{xx}_{i}}-\frac{{a}_{{xx}_{i}}}{b}\right)+b\dot{\widehat{h}}\left(\widehat{h}-h\right)\end{aligned}$$

Substituting ${F}_{{c}_{x}}={u}_{{2}_{xx}}$ from Eq. (86) in Eq. (93) gives:

94 $$\begin{aligned}{\dot{V}}_{xx}& = {S}_{{aug}_{xx}}\left(2{n}_{t}\dot{y}+{n}_{t}^{2}x+{\dot{n}}_{t}y-\frac{\mu }{{r}_{c}^{2}}+\mu {f}_{{xx}_{2}}\left(x\right)+\frac{\mu }{{r}_{c}^{3}}{f}_{{xx}_{1}}\left(x\right)-\frac{\mu }{{r}_{c}^{3}}x\right. \\ & \quad \left. +b\left(-\widehat{h}\left[{u}_{{1}_{xx}}+\left({D}_{xx}+{\varepsilon }_{xx}\right)sign\left({s}_{xx}\right)\right]-\sum_{i=1}^{3}{\widehat{\alpha }}_{{xx}_{i}}{g}_{x{x}_{i}}-\sum_{i=1}^{2}{\widehat{a}}_{{xx}_{i}}{f}_{x{x}_{i}}\left(x\right)\right)+{a}_{{d}_{x}}-{\ddot{x}}_{d}+{\lambda }_{0}{\dot{e}}_{x}\right)\\ & \quad -b\sum_{i=1}^{3}{\dot{\widehat{\alpha }}}_{{xx}_{i}}\left({\widehat{\alpha }}_{{xx}_{i}}-\frac{{\alpha }_{{xx}_{i}}}{b}\right)-b\sum_{i=1}^{2}{\dot{\widehat{a}}}_{{xx}_{i}}\left({\widehat{a}}_{{xx}_{i}}-\frac{{a}_{{xx}_{i}}}{b}\right)+b\dot{\widehat{h}}\left(\widehat{h}-h\right)\end{aligned}$$

Equation (94) can be rewritten as follows:

95 $$\begin{aligned}{\dot{V}}_{xx} & = \left(2{n}_{t}-b{\widehat{\alpha }}_{{xx}_{1}}\right)\left(\dot{y}{S}_{{aug}_{xx}}+{\dot{\widehat{\alpha }}}_{{xx}_{1}}\right)+\left(\left({n}_{t}^{2}-\frac{\mu }{{r}_{C}^{3}}\right)-b{\widehat{\alpha }}_{{xx}_{2}}\right)\left(x{S}_{{aug}_{xx}}+{\dot{\widehat{\alpha }}}_{{xx}_{2}}\right)+\left({\dot{n}}_{t}-b{\widehat{\alpha }}_{{xx}_{3}}\right)\left(y{S}_{{aug}_{xx}}+{\dot{\widehat{\alpha }}}_{{xx}_{3}}\right)\\ & \quad +\left(\mu -b{\widehat{a}}_{{xx}_{1}}\right)\left({f}_{{xx}_{2}}\left(x\right){S}_{{aug}_{xx}}+{\dot{\widehat{a}}}_{{xx}_{2}}\right)+\left(\frac{\mu }{{r}_{C}^{3}}-b{\widehat{a}}_{{xx}_{2}}\right)\left({f}_{{xx}_{1}}\left(x\right){S}_{{aug}_{xx}}+{\dot{\widehat{a}}}_{{xx}_{1}}\right)-\frac{\mu }{{r}_{c}^{2}}{S}_{{aug}_{xx}}\\ & \quad +{S}_{{aug}_{xx}}\left(-b\widehat{h}\left[{u}_{{1}_{xx}}+\left({D}_{xx}+{\varepsilon }_{xx}\right)sign\left({s}_{xx}\right)\right]+{a}_{{d}_{x}}-{\ddot{x}}_{d}+{\lambda }_{0}{\dot{e}}_{xx}\right)+\dot{\widehat{h}}\left(b\widehat{h}-1\right)\end{aligned}$$

Choosing the following adaptation laws (Eqs. (96) to (101)), will change Eq. (95) to the form of Eq. (102).

96 $${\dot{\widehat{\alpha }}}_{{xx}_{1}}=-\dot{y}{S}_{{aug}_{xx}}$$

97 $${\dot{\widehat{\alpha }}}_{{xx}_{2}}=-x{S}_{{aug}_{xx}}$$

98 $${\dot{\widehat{\alpha }}}_{{xx}_{3}}=-y{S}_{{aug}_{xx}}$$

99 $${\dot{\widehat{a}}}_{{xx}_{1}}=-{f}_{{xx}_{1}}\left(x\right){S}_{{aug}_{xx}}$$

100 $${\dot{\widehat{a}}}_{{xx}_{2}}=-{f}_{{xx}_{2}}\left(x\right){S}_{{aug}_{xx}}$$

101 $$\dot{\widehat{h}}=\left({D}_{xx}+{\varepsilon }_{xx}\right)sign\left({s}_{xx}\right){S}_{{aug}_{xx}}+{u}_{{1}_{xx}}{S}_{{aug}_{xx}}$$

102 $${\dot{V}}_{xx}=-\frac{\mu }{{r}_{C}^{2}}{S}_{{aug}_{xx}}+{a}_{{d}_{x}}{S}_{{aug}_{xx}}-\left({D}_{xx}+{\varepsilon }_{xx}\right)sign\left({s}_{xx}\right){S}_{{aug}_{xx}}$$

We are going to show that ${\dot{V}}_{xx}\le -{\varepsilon }_{xx}\left|{S}_{{aug}_{xx}}\right|$, where ${\varepsilon }_{xx}$ is a positive constant.

thus Eq. (102) can be written as:

103 $$\left|-\frac{\mu }{{r}_{C}^{2}}+{a}_{{d}_{x}}\right|\left|{S}_{{aug}_{xx}}\right|\le -{\varepsilon }_{xx}\left|{S}_{{aug}_{x}}\right|+\left({D}_{xx}+{\varepsilon }_{xx}\right)sign\left({s}_{xx}\right)\left|{S}_{{aug}_{xx}}\right|$$

The asymptotic stability condition is:

104 $$\left|-\frac{\mu }{{r}_{C}^{2}}+{A}_{{d}_{x}}\right|\le {D}_{xx}$$

$\frac{\mu }{{r}_{C}^{2}}$ is a positive calculable parameter and ${A}_{{d}_{x}}$ denotes the upper bound of ${a}_{{d}_{x}}$.

Despite X-axis that ${f}_{{xx}_{1}}\left(x\right)$ and ${f}_{{xx}_{2}}\left(x\right)$ were nonlinear terms, there is no nonlinearity in Y and Z axes.

For Y-axis, by selecting ${\alpha }_{{yy}_{1}}=-2{n}_{t}$, ${\alpha }_{{yy}_{2}}=\left({n}_{t}^{2}-\frac{\mu }{{r}_{C}^{3}}\right)$, and ${\alpha }_{{yy}_{3}}={\dot{n}}_{t}$ we have:

105 $$\ddot{y}={\alpha }_{{yy}_{1}}\dot{x}+{\alpha }_{{yy}_{2}}y-{\alpha }_{{yy}_{3}}x+b{F}_{{c}_{y}}+{a}_{{d}_{y}}$$

Applying the expressed process to Y-axis and choosing the following sliding surface, control signal, and Lyapunov candidate will lead to

106 $${S}_{{aug}_{yy}}={s}_{yy}-{d}_{yy}sign({s}_{yy})$$

107 $${s}_{yy}={\dot{e}}_{yy}+{\lambda }_{0}{e}_{yy}$$

108 $${u}_{{2}_{yy}}=-\widehat{h}\left[{u}_{{1}_{yy}}+\left({D}_{yy}+{\varepsilon }_{yy}\right)sign\left({s}_{yy}\right)\right]-{\widehat{\alpha }}_{{yy}_{1}}\dot{x}-{\widehat{\alpha }}_{{yy}_{2}}y+{\widehat{\alpha }}_{{yy}_{3}}x$$

where $${u}_{{1}_{yy}}={-\ddot{y}}_{d}+{\lambda }_{0}{\dot{e}}_{yy}$$.

109 $${V}_{yy}=\frac{1}{2}{S}_{{aug}_{yy}}^{2}-\frac{1}{2}b{\left({\widehat{\alpha }}_{{yy}_{1}}-\frac{{\alpha }_{{yy}_{1}}}{b}\right)}^{2}-\frac{1}{2}b{\left({\widehat{\alpha }}_{{yy}_{2}}-\frac{{\alpha }_{{yy}_{2}}}{b}\right)}^{2}+\frac{1}{2}b{\left({\widehat{\alpha }}_{{yy}_{3}}-\frac{{\alpha }_{{yy}_{3}}}{b}\right)}^{2}+\frac{1}{2}b{\left(\widehat{h}-h\right)}^{2}$$

where ${\varepsilon }_{yy}$ and ${D}_{yy}$ are positive constants.

The time derivative of ${V}_{yy}$ is:

110 $${\dot{V}}_{yy}={S}_{{aug}_{yy}}{\dot{S}}_{{aug}_{yy}}-b{\dot{\widehat{\alpha }}}_{{yy}_{1}}\left({\widehat{\alpha }}_{{yy}_{1}}-\frac{{\alpha }_{{yy}_{1}}}{b}\right)-b{\dot{\widehat{\alpha }}}_{{yy}_{2}}\left({\widehat{\alpha }}_{{yy}_{2}}-\frac{{\alpha }_{{yy}_{2}}}{b}\right)+b{\dot{\widehat{\alpha }}}_{{yy}_{3}}\left({\widehat{\alpha }}_{{yy}_{3}}-\frac{{\alpha }_{{yy}_{3}}}{b}\right)+\dot{\widehat{h}}\left(b\widehat{h}-1\right)$$

111 $${\dot{V}}_{yy}={S}_{{aug}_{yy}}\left(\ddot{y}-{\ddot{y}}_{d}+{\lambda }_{0}{\dot{e}}_{yy}\right)-b{\dot{\widehat{\alpha }}}_{{yy}_{1}}\left({\widehat{\alpha }}_{{yy}_{1}}-\frac{{\alpha }_{{yy}_{1}}}{b}\right)-b{\dot{\widehat{\alpha }}}_{{yy}_{2}}\left({\widehat{\alpha }}_{{yy}_{2}}-\frac{{\alpha }_{{yy}_{2}}}{b}\right)+b{\dot{\widehat{\alpha }}}_{{yy}_{3}}\left({\widehat{\alpha }}_{{yy}_{3}}-\frac{{\alpha }_{{yy}_{3}}}{b}\right)+\dot{\widehat{h}}\left(b\widehat{h}-1\right)$$

Substituting Eqs. (105) and (108) in Eq. (111) gives:

112 $$\begin{aligned}{\dot{V}}_{yy}& = \left({\alpha }_{{yy}_{1}}-b{\widehat{\alpha }}_{{yy}_{1}}\right)\left(\dot{x}{S}_{{aug}_{yy}}+{\dot{\widehat{\alpha }}}_{{yy}_{1}}\right)+\left({\alpha }_{{yy}_{2}}-b{\widehat{\alpha }}_{{yy}_{2}}\right)\left(y{S}_{{aug}_{yy}}+{\dot{\widehat{\alpha }}}_{{yy}_{2}}\right)+\left(-{\alpha }_{{yy}_{3}}+b{\widehat{\alpha }}_{{yy}_{3}}\right)\left(x{S}_{{aug}_{yy}}+{\dot{\widehat{\alpha }}}_{{yy}_{3}}\right)\\ & \quad +b{S}_{{aug}_{yy}}\left(-\widehat{h}\left[{u}_{{1}_{yy}}+\left({D}_{yy}+{\varepsilon }_{yy}\right)sign\left({s}_{yy}\right)\right]\right)+{a}_{{d}_{y}}-{\ddot{y}}_{d}+{\lambda }_{0}{\dot{e}}_{yy}+\dot{\widehat{h}}\left(b\widehat{h}-1\right)\end{aligned}$$

The adaptation law is chosen as follows:

113 $${\dot{\widehat{\alpha }}}_{{yy}_{1}}=-\dot{x}{S}_{{aug}_{yy}}$$

114 $${\dot{\widehat{\alpha }}}_{{yy}_{2}}=-y{S}_{{aug}_{yy}}$$

115 $${\dot{\widehat{\alpha }}}_{{yy}_{3}}=-x{S}_{{aug}_{yy}}$$

116 $$\dot{\widehat{h}}=\left({D}_{yy}+{\varepsilon }_{yy}\right)sign\left({s}_{yy}\right){S}_{{aug}_{yy}}+{u}_{{1}_{yy}}{S}_{{aug}_{yy}}$$

Substituting adaptation laws in Eq. (112) to prove that ${\dot{V}}_{yy}\le -{\varepsilon }_{yy}\left|{S}_{{aug}_{yy}}\right|$, where ${\varepsilon }_{yy}$ is a positive constant, give:

117 $${\dot{V}}_{yy}=-\left({D}_{yy}+{\varepsilon }_{yy}\right)sign\left({s}_{yy}\right){S}_{{aug}_{yy}}+{S}_{{aug}_{yy}}{a}_{{d}_{y}}\le -{\varepsilon }_{yy}\left|{S}_{{aug}_{yy}}\right|$$

The asymptotic stability condition of the Y-axis is:

118 $${D}_{yy}\ge \left|{A}_{{d}_{y}}\right|$$

where ${A}_{{d}_{y}}$ is the upper bound of ${a}_{{d}_{y}}$.

As same as Y-axis, we can design the controller for Z-axis as follows:

119 $${S}_{{aug}_{zz}}={s}_{zz}-{d}_{zz}sign({s}_{zz})$$

120 $${s}_{zz}={\dot{e}}_{zz}+{\lambda }_{0}{e}_{zz}$$

121 $${u}_{{2}_{zz}}=-\widehat{h}\left[{u}_{{1}_{zz}}+\left({D}_{zz}+{\varepsilon }_{zz}\right)sign\left({s}_{zz}\right)\right]-{\widehat{a}}_{{zz}_{1}}z$$

where ${a}_{{zz}_{1}}=-\frac{\mu }{{r}_{C}^{3}},$ and ${u}_{{1}_{zz}}$ is defined as:

122 $${u}_{{1}_{zz}}={-\ddot{z}}_{d}+{\lambda }_{0}{\dot{e}}_{zz}$$

The Lyapunov candidate and its time-derivative are:

123 $${V}_{zz}=\frac{1}{2}{S}_{{aug}_{yy}}^{2}-\frac{1}{2}b{\left({\widehat{a}}_{{zz}_{1}}-\frac{{a}_{{zz}_{1}}}{b}\right)}^{2}+\frac{1}{2}b{\left(\widehat{h}-h\right)}^{2}$$

124 $${\dot{V}}_{zz}={S}_{{aug}_{yy}}{\dot{S}}_{{aug}_{yy}}-b{\dot{\widehat{a}}}_{{zz}_{1}}\left({\widehat{a}}_{{zz}_{1}}-\frac{{a}_{{zz}_{1}}}{b}\right)+\dot{\widehat{h}}\left(b\widehat{h}-1\right)$$

125 $${\dot{V}}_{zz}={S}_{{aug}_{zz}}\left(\ddot{z}-{\ddot{z}}_{d}+{\lambda }_{0}{\dot{e}}_{zz}\right)-b{\dot{\widehat{a}}}_{{zz}_{1}}\left({\widehat{a}}_{{zz}_{1}}-\frac{{a}_{{zz}_{1}}}{b}\right)+\dot{\widehat{h}}\left(b\widehat{h}-1\right)$$

Substituting $\ddot{z}$ from Eq. (30) and ${F}_{{c}_{z}}={u}_{{2}_{zz}}$ from Eq. (121) in Eq. (125) gives:

126 $${\dot{V}}_{zz}=\left({\alpha }_{{zz}_{1}}-b{\widehat{\alpha }}_{{zz}_{1}}\right)\left(z{S}_{{aug}_{zz}}+{\dot{\widehat{\alpha }}}_{{zz}_{1}}\right)-\widehat{h}{S}_{{aug}_{zz}}b\left({D}_{zz}+{\varepsilon }_{zz}\right)sign\left({s}_{zz}\right)+{S}_{{aug}_{zz}}{a}_{{d}_{z}}+\left(1-\widehat{h}b\right){S}_{{aug}_{zz}}\left({u}_{{1}_{zz}}\right)+\dot{\widehat{h}}\left(b\widehat{h}-1\right)$$

Adaptation law is chosen as:

127 $${\dot{\widehat{\alpha }}}_{{zz}_{1}}=-z{S}_{{aug}_{zz}}$$

128 $$\dot{\widehat{h}}=\left({D}_{zz}+{\varepsilon }_{zz}\right)sign\left({s}_{zz}\right){S}_{{aug}_{zz}}+{S}_{{aug}_{zz}}{u}_{{1}_{zz}}$$

Substituting adaptation laws (Eqs. (127) and (128)) in ${\dot{V}}_{zz}$ gives:

129 $${\dot{V}}_{zz}=-\left({D}_{zz}+{\varepsilon }_{zz}\right)sign\left({s}_{zz}\right){S}_{{aug}_{zz}}+{S}_{{aug}_{zz}}{a}_{{d}_{z}}$$

Selecting ${D}_{zz}\ge {A}_{{d}_{z}}$ guarantee the inequality ${\dot{V}}_{zz}\le -{\varepsilon }_{zz}\left|{S}_{{aug}_{zz}}\right|$, where ${\varepsilon }_{zz}$ is a positive constant and ${A}_{{d}_{z}}=max\left(\left|{a}_{{d}_{z}}\right|\right)$.

**Note:** One of the important specifics of the proposed controller is that both translational (${\ddot{x}}_{d}, {\ddot{y}}_{d}, {\ddot{z}}_{d}$) and orientation $\left({\dot{\omega }}_{{e}_{{x}_{d}}}, {\dot{\omega }}_{{e}_{{y}_{d}}}, {\dot{\omega }}_{{e}_{{z}_{d}}}\right)$ accelerations (for example see Eqs. (56) and (88)) can be set by the user.

## Simulation

In this section, the results of numerical simulations are presented to demonstrate the abilities and performance of the controller. In this regard, three simulation scenarios are considered based on three different OOS missions. In all three scenarios, the mass and moment of inertia tensor are considered as follows:

$${m}_{c}=100 [\text{kg}]\,\,\, {I}_{c}=\left[\begin{array}{ccc}11& -0.09& -0.4\\ -0.09& 14& -0.08\\ -0.4& -0.08& 13\end{array}\right] [\text{kg}.{\text{m}}^{2}]$$

$${m}_{t}=5425 [\text{kg}]\,\,\, {I}_{t}=\left[\begin{array}{ccc}3336& -135& -154\\ -135& 3184& -135\\ -154& -135& 2423\end{array}\right] [\text{kg}.{\text{m}}^{2}]$$

Also, the control parameters are chosen in Table 2:

**Table 2:** Controller parameters.

Attitude motion controller:			
${D}_{x}={D}_{y}={D}_{z}=0.5$	${\varepsilon }_{x}={\varepsilon }_{y}={\varepsilon }_{z}=0.05$	${d}_{x}={d}_{y}={d}_{z}=0.5$	${\lambda }_{0}=3$
Translational motion controller:			
${D}_{xx}={D}_{yy}={D}_{zz}=50$	${\varepsilon }_{xx}={\varepsilon }_{yy}={\varepsilon }_{zz}=0.05$	${d}_{xx}={d}_{yy}={d}_{zz}=2,$	${\lambda }_{0}=0.1$
* The initial amount of all the parameters (for adaptation laws) is assumed to be zero			

The actuator parameters are presented in Table 3:

**Table 3:** Actuator specifications.

Reaction wheels:			
Max Torque (${T}_{{w}_{max}}$): $0.1$ Nm	Max Rate (${\omega }_{{w}_{max}}$): 7000 rpm	Delay: 2 ms	Incidence angle ($\beta$):$30 [\text{deg}]$
Inertia (${I}_{w}$):$0.001364 {\text{kg}\cdot \text{m}}^{2}$	Mass: 0.9 kg		
Reaction Thrusters:			
Max Force: 1 N	${\text{I}}_{\text{sp}}$: 227 s	${\text{U}}_{\text{on}}$: 0.25	${\text{U}}_{\text{off}}$:0.15
${\text{k}}_{\text{m}}$: 10	${\text{T}}_{\text{s}}$: 0.2 s		

The numerical simulations are implemented using MATLAB-Simulink. The differential equations associated with the mathematical models of the pursuer, target, adaptation laws, and controllers are integrated using a fixed time-step Runge–Kutta integration method (100 Hz).

### Scenario I

For the first scenario, we assumed that the chaser spacecraft is approaching a wide-body communication satellite orbiting in Geostationary Orbit (GEO), which is running low on its fuel tank and needs refueling. The target is alive and stable in the nadir-pointing attitude $\left({\overrightarrow{q}}_{t}\left(0\right)={\left[0, 0, 0, 1\right]}^{T}\right)$ and its initial angular velocity is equal to the orbital mean motion (${\overrightarrow{\omega }}_{t}\left(0\right)={\left[0,{n}_{t}, 0\right]}^{T} [\text{deg}/\text{s}]$). The initial attitude and angular velocity of the chaser spacecraft is assumed to be $\left({\overrightarrow{q}}_{C}\left(0\right)={\left[-0.3257, 0.5957, 0. 6722, 0.2954\right]}^{T}\right)$ and ${\overrightarrow{\omega }}_{C}\left(0\right)={\left[3, 1, 5\right]}^{T} [\text{deg}/\text{s}]$, respectively. The relative distance is assumed to be about 80 m ($\left[x, y, z\right]=\left[50, 0, 60\right] [\text{m}]$) and relative velocity is $\left(\left[\dot{x}, \dot{y}, \dot{z}\right]=\left[3, 0, 0\right] [\frac{{\text{m}}}{{\text{sec}}}]\right)$. The control objective is to drive the chaser spacecraft to the distance of $\left[0, 0, 0\right] [\text{m}]$ from the target in the LVLH frame, while the relative attitude is synchronized.

**Figure 8:**
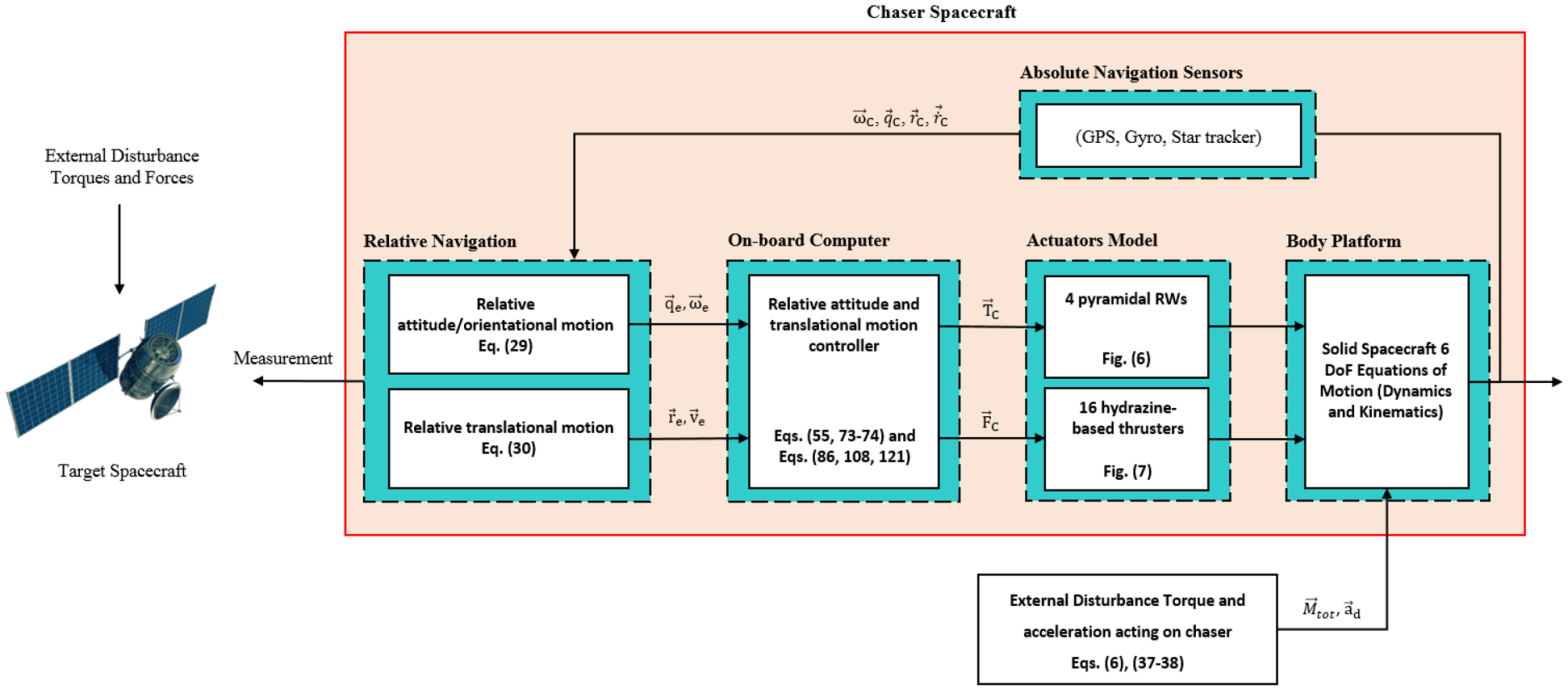
Block diagram of relative motion control.

The block diagram of relative pose motion control is depicted in Fig. 8.

**Figure 9:**
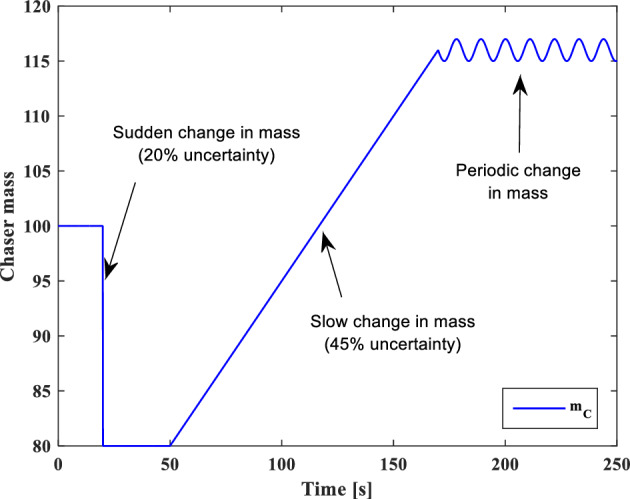
Chaser mass variation profile.

The variations in chaser mass that represent uncertainty are seen in Fig. 9. At the first time, it is assumed that mass decreases by 20% at $t=20\text{s}$ (suddenly), then rises slowly and continuously from 80 $\left[\text{kg}\right]$ at $t=50\text{s}$ to 116 $\left[\text{kg}\right]$ at $t=170\text{s}$ before experiencing periodic sinusoidal changes from 114 to 118 $\left[\text{kg}\right]$.

**Figure 10:**
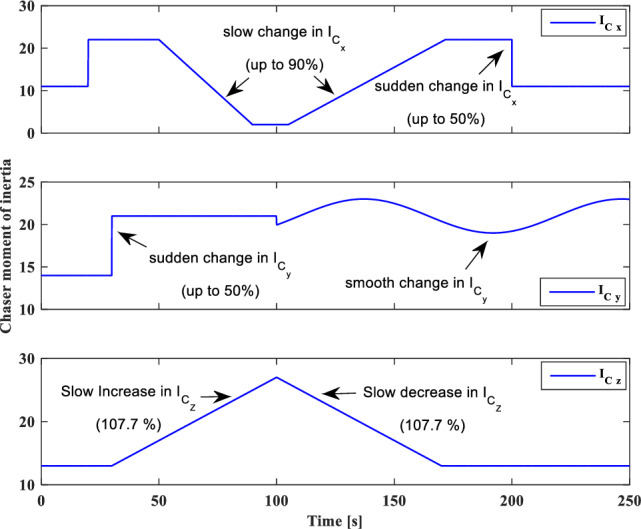
Chaser moment of inertia variation profile.

Note: obviously, the changes in mass and moment of inertia of a satellite (chaser) due to fuel consumption, the activation of a mechanism, etc. are much milder than what is considered in the above figures. In real-world conditions, the mass of the satellite decreases slowly due to fuel consumption, or a piece of satellite equipment may be cut off by a space debris collision, which leads to a sudden decrease in the mass. The moment of inertia may also show a periodic behavior due to fuel slushing (at the end of life). So, a sharp increase in the mass or moment of inertia is not a realistic phenomenon. We have done a strict simulation only to challenge the robustness and performance of the controller. The changes in chaser dynamic parameters (mass and moment of inertia) are considered similar in all three scenarios.

The variations in the chaser moment of inertia are illustrated in Fig. 10. For ${I}_{{C}_{x}}$, we have a sudden increase at $t=20\text{s}$ from 11 to 22 $\left[\text{kg}.{\text{m}}^{2}\right]$, and two slow changes during $40\text{s}$ with different slopes and a sudden increase at $t=200\text{s}$. ${I}_{{C}_{y}}$ experience a sudden change at $t=30\text{s}$ from 14 to 21 $\left[\text{kg}.{\text{m}}^{2}\right]$ before a smooth periodic change at $t=100\text{s}$ with domain from 19 to 23 $\left[\text{kg}.{\text{m}}^{2}\right]$. ${I}_{{C}_{z}}$ arises from 13 $\left[\text{kg}.{\text{m}}^{2}\right]$ to 27 $\left[\text{kg}.{\text{m}}^{2}\right]$ at $t=30\text{s}$ and then decreases from 27 to 13 $\left[\text{kg}.{\text{m}}^{2}\right]$ with the same slope.

The simulation results of the first scenario are discussed in the next.

**Figure 11:**
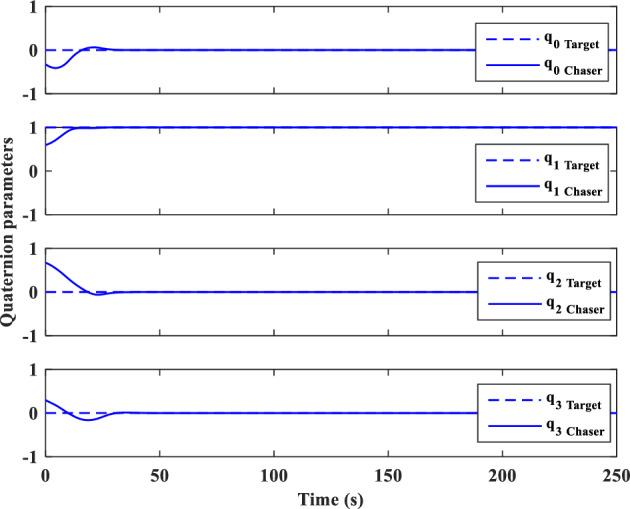
Chaser and target quaternion parameters (Scenario 1).

**Figure 12:**
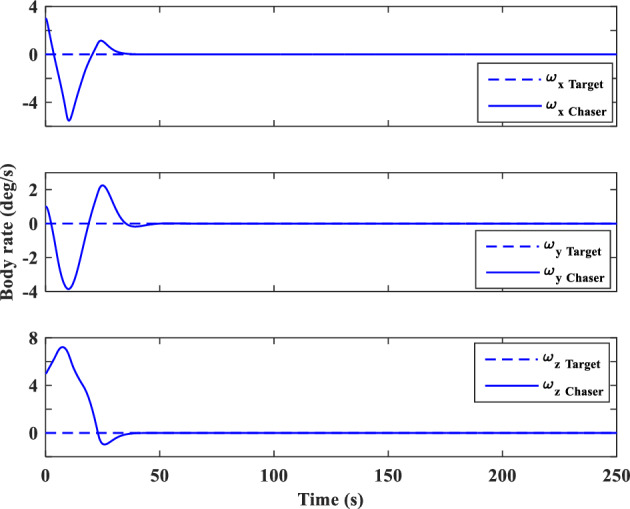
Chaser and target angular velocity (Scenario 1).

**Figure 13:**
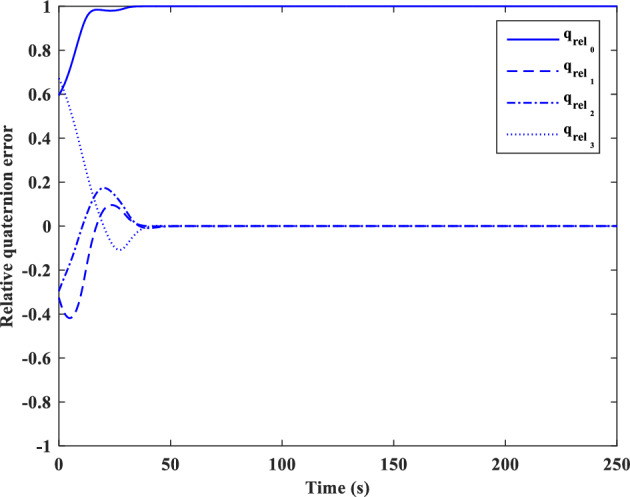
Relative quaternion parameters (Scenario 1).

**Figure 14:**
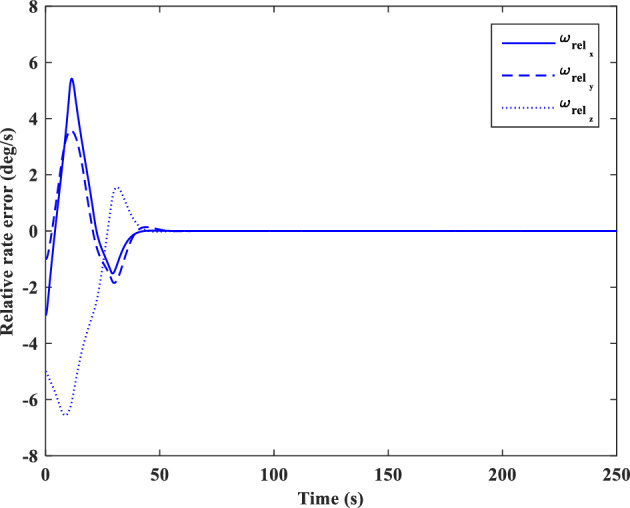
Relative angular velocity (Scenario 1).

Quaternion parameters and the angular velocity of the chaser and the target spacecraft are shown in Figs. 11 and 12, respectively. The relative quaternion and angular velocity of scenario 1 are depicted in Figs. 13 and 14, respectively. Clearly, the controller was successful in achieving tracking goals after $60s$.

**Figure 15:**
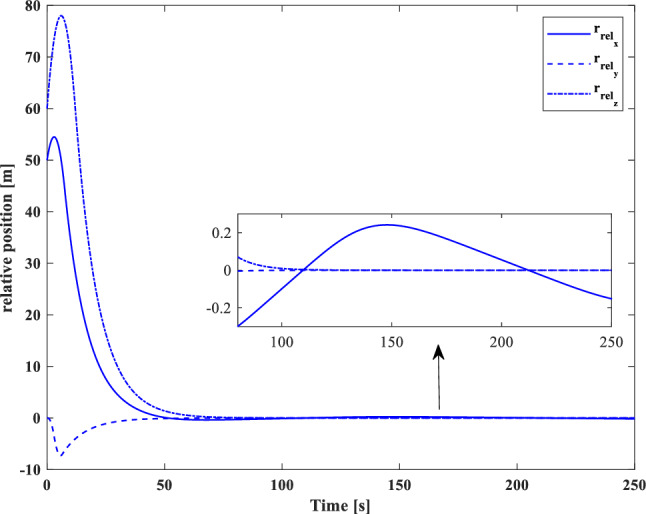
Relative position (Scenario 1).

**Figure 16:**
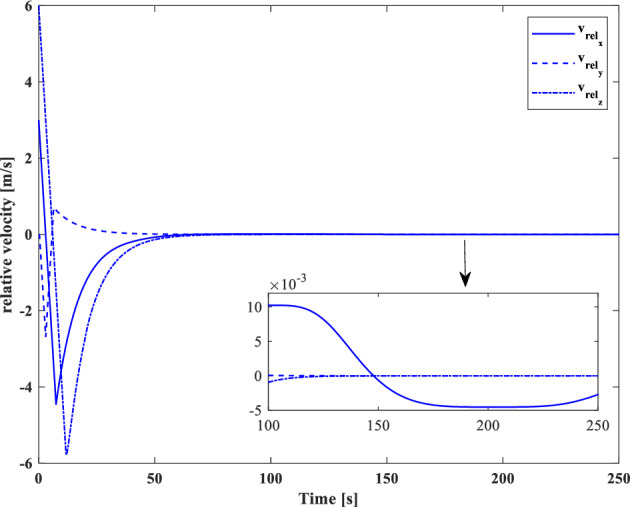
Relative velocity (Scenario 1).

Figures 15 and 16 show the relative position/distance and linear velocity of the chaser and target spacecraft. The relative distance converges to zero after about $100s$.

**Figure 17:**
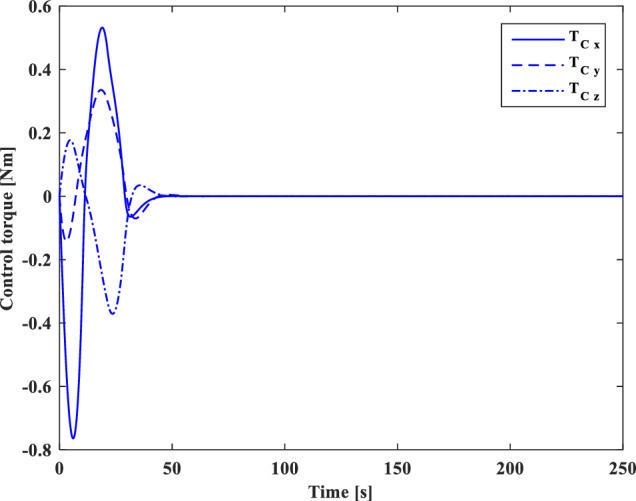
Attitude motion control effort (Scenario 1).

**Figure 18:**
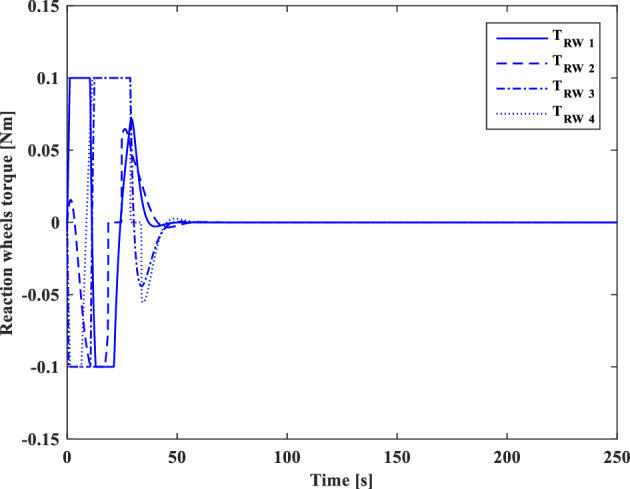
Reaction wheels torque (Scenario 1).

The control effort (calculated by the control algorithm) and the attitude actuators' output torque are depicted in Figs. 17 and 18, respectively.

**Figure 19:**
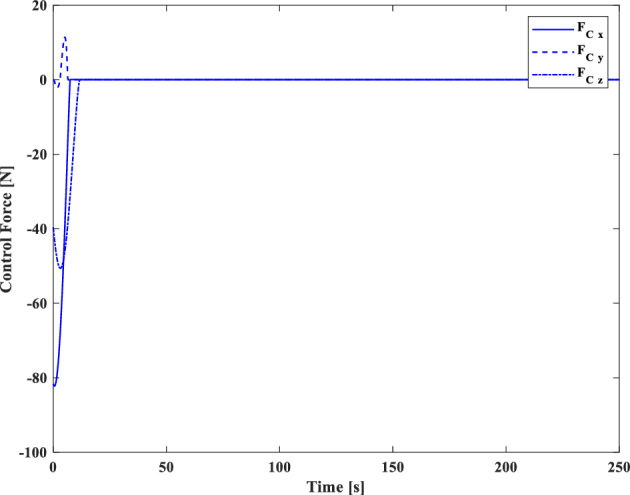
Translational motion control effort (Scenario 1).

**Figure 20:**
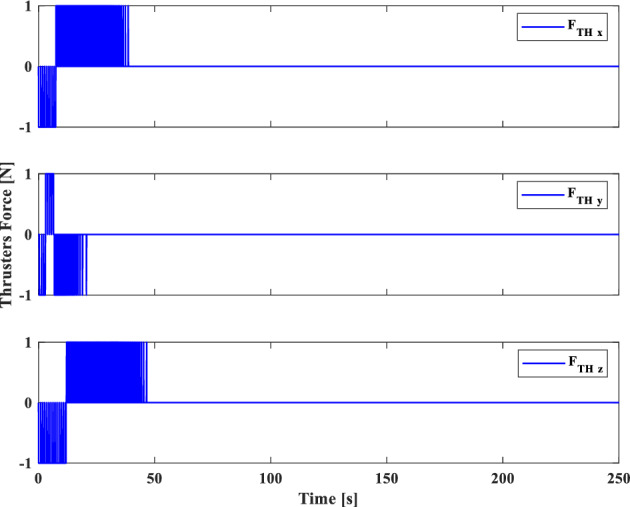
PWPF thruster’s force (Scenario 1).

Similarly, control effort and thruster’s force are depicted in Figs. 19 and 20.

**Figure 21:**
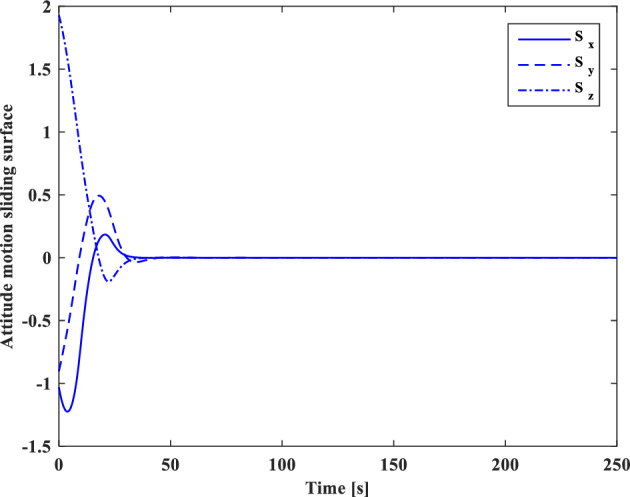
Attitude motion sliding surface (Scenario 1).

**Figure 22:**
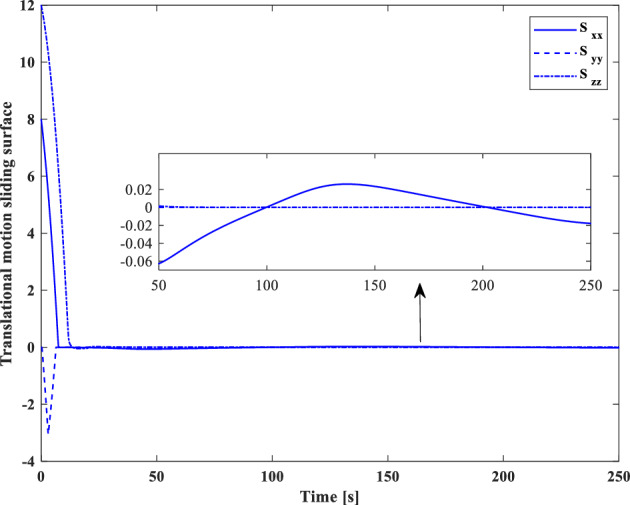
Translational motion sliding surface (Scenario 1).

Note: the external disturbances were negligible in scenario 1 due to the GEO high altitude and a simple slow-rate rest-to-rest maneuver. Indeed, the uncertainty of mass and moment of inertia were the only challenging parameters in this case.

The sliding surface of both attitude and translational motion are shown in Figs. 21 and 22, respectively. The sliding surface is known as an interpretation of the error, which has reached zero in all three axis as seen in the figures below.

### Scenario II

In the second scenario, it is assumed that the target satellite is out of control and rotates around itself with a constant angular velocity. The target’s initial attitude is $\left({\overrightarrow{q}}_{t}\left(0\right)={\left[-0.3289., 0.1927, 0.2366, 0.8937\right]}^{T}\right)$ and its angular velocity is (${\overrightarrow{\omega }}_{t}={\left[3, 5, -4\right]}^{T} [\text{deg}/\text{s}]$). The initial attitude and angular velocity of the chaser spacecraft is assumed to be $\left({\overrightarrow{q}}_{c}\left(0\right)={\left[0, 0, 0, 1\right]}^{T}\right)$ and ${\overrightarrow{\omega }}_{c}\left(0\right)={\left[0, 0, 0\right]}^{T} [\text{deg}/\text{s}]$, respectively. The relative distance is assumed to be about 80 m ($\left[x, y, z\right]=\left[54, 0, 60\right] [\text{m}]$) and relative velocity is $\left(\left[\dot{x}, \dot{y}, \dot{z}\right]=\left[-3, 0, 5\right] [\frac{{\text{m}}}{{\text{sec}}}]\right)$. The control objective is to drive the chaser spacecraft to the distance of $\left[5, 0, 0\right] [\text{m}]$ from the target in the LVLH frame, while the relative attitude is synchronized.

**Figure 23:**
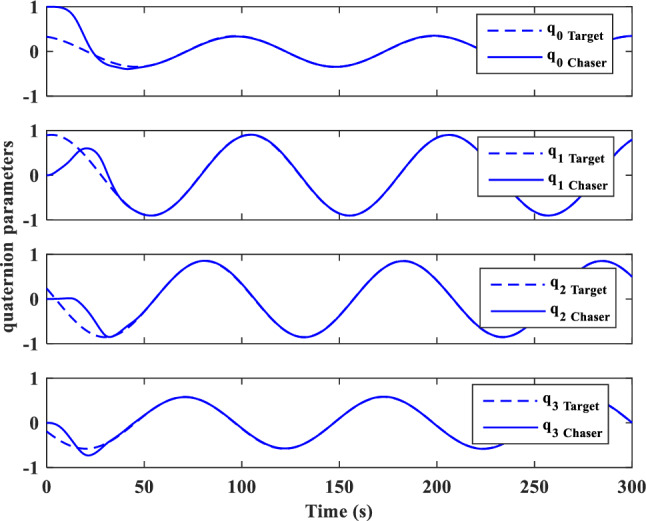
Chaser and target quaternion parameters (Scenario 2).

**Figure 24:**
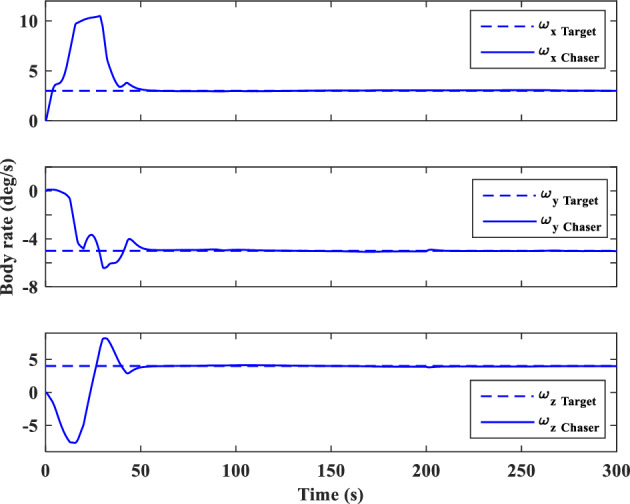
Chaser and target angular velocity (Scenario 2).

The quaternion parameters and angular velocity of the chaser and target spacecraft are shown in Figs. 23 and 24, respectively.

**Figure 25:**
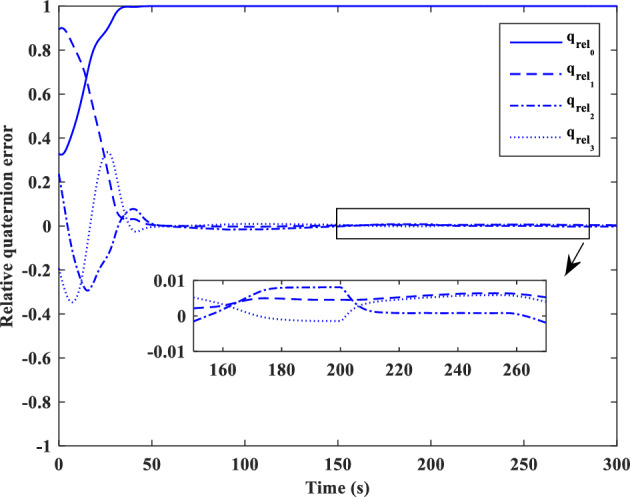
Relative quaternion parameters (Scenario 2).

**Figure 26:**
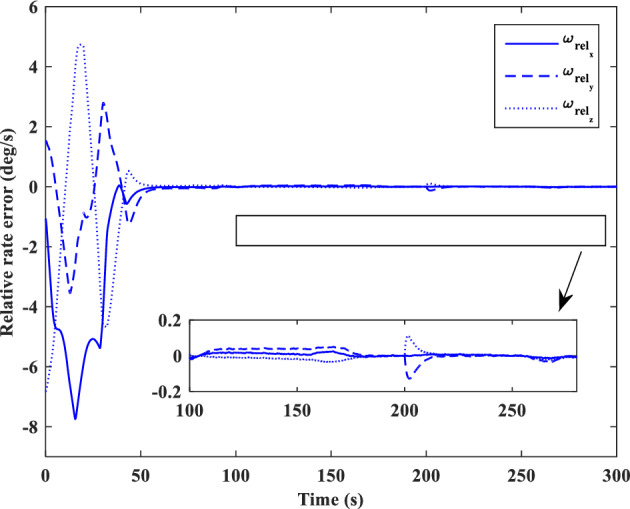
Relative angular velocity (Scenario 2).

The relative attitude and angular velocity of the two spacecraft are depicted in Figs. 25 and 26, respectively. As it is clear from these plots, attitude synchronization is achieved in a short time.

**Figure 27:**
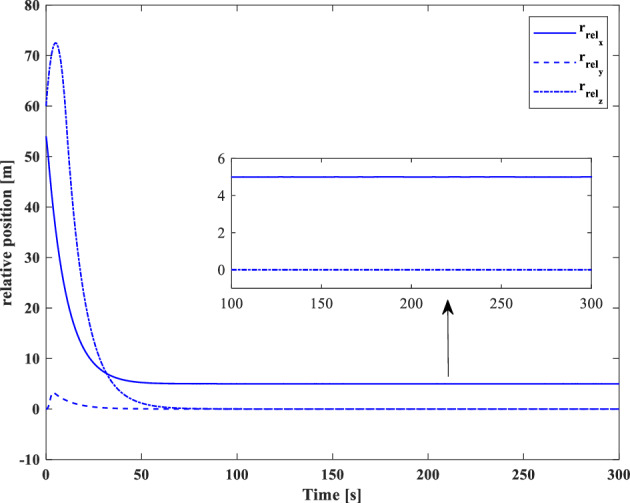
Relative position (Scenario 2).

**Figure 28:**
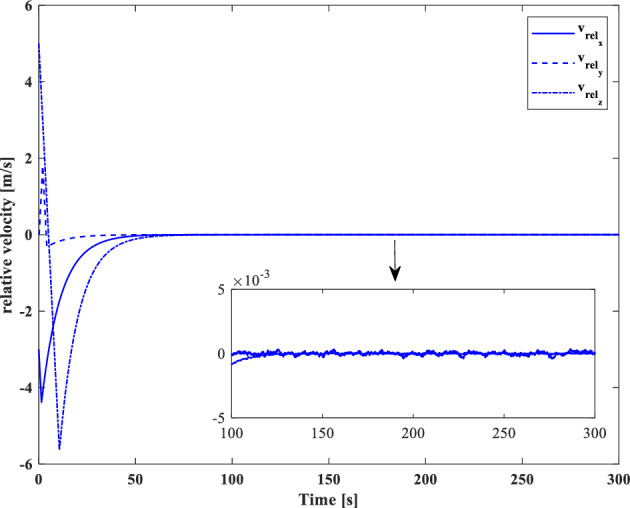
Relative velocity (Scenario 2).

Relative position and velocity of the spacecraft are shown in Figs. 27 and 28.

By putting plots 25 to 28 together, it can be concluded that the error of relative attitude and translational motion have reached zero simultaneously.

Control effort and the torque produced by wheels are shown in Figs. 21 and 22, respectively.

Control effort of translational motion and thrusters output are illustrated in Figs. 31 and 32.

As indicated by the sliding surface (Figs. 33 and 34), which provides an interpretation and expression of the error, the mission requirements have been satisfied.

The simulation results (Figs. 23, 24, 25, 26, 27, 28, 29, 30, 31, 32, 33, 34) prove that the controller is successful in meeting the requirements of the approximation with a target under constant rotational velocity.

**Figure 29:**
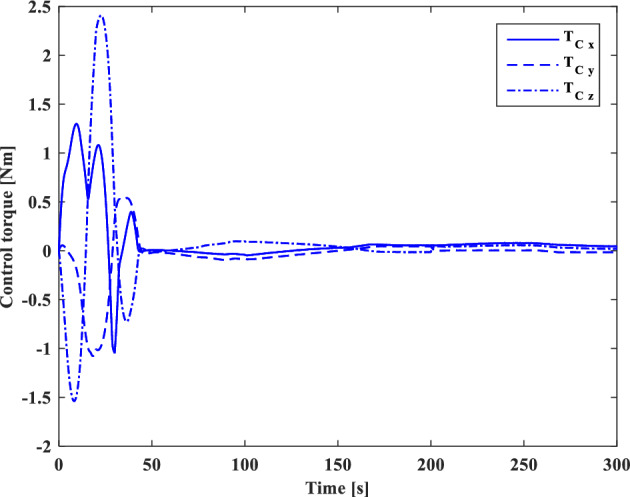
Attitude motion control effort (Scenario 2).

**Figure 30:**
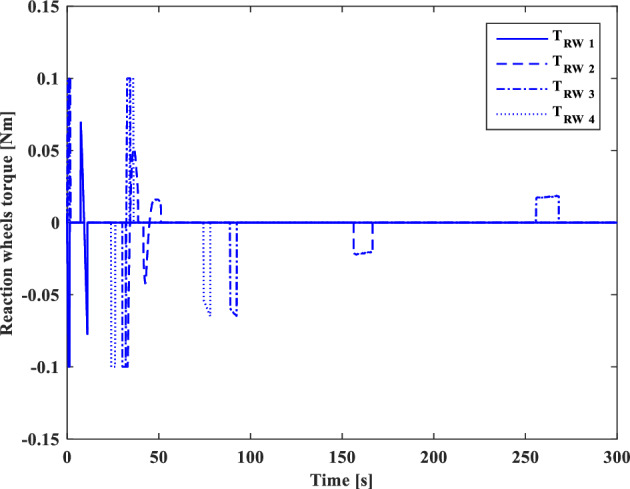
Reaction wheels torque (Scenario 2).

**Figure 31:**
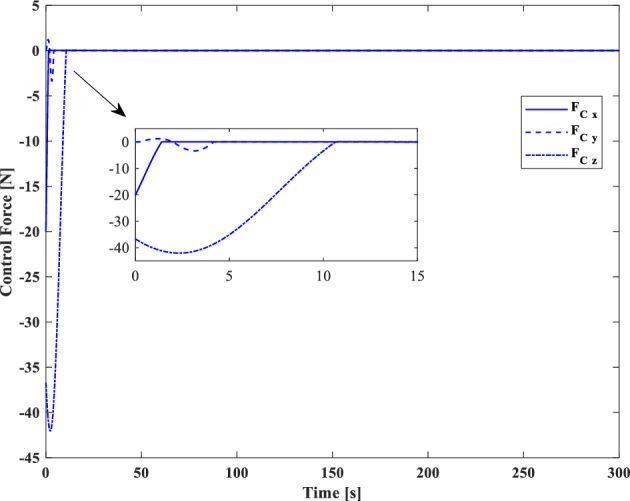
Translational motion control effort (Scenario 2).

**Figure 32:**
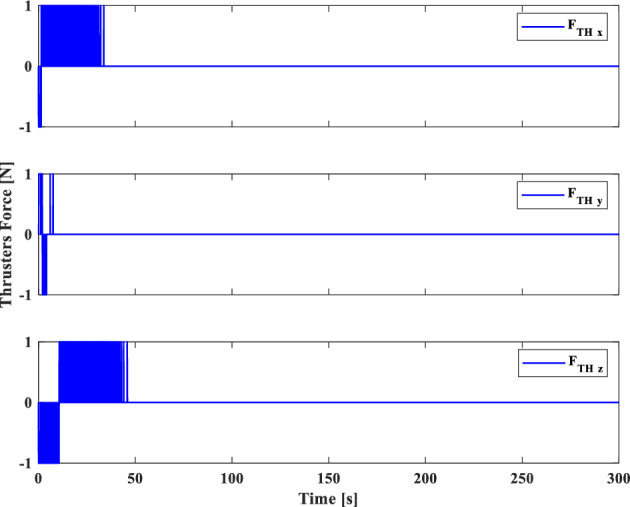
PWPF thruster’s force (Scenario 2).

**Figure 33:**
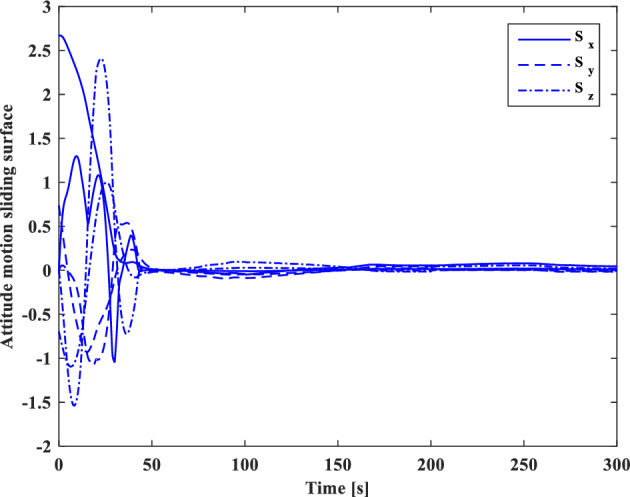
Attitude motion sliding surface (Scenario 2).

**Figure 34:**
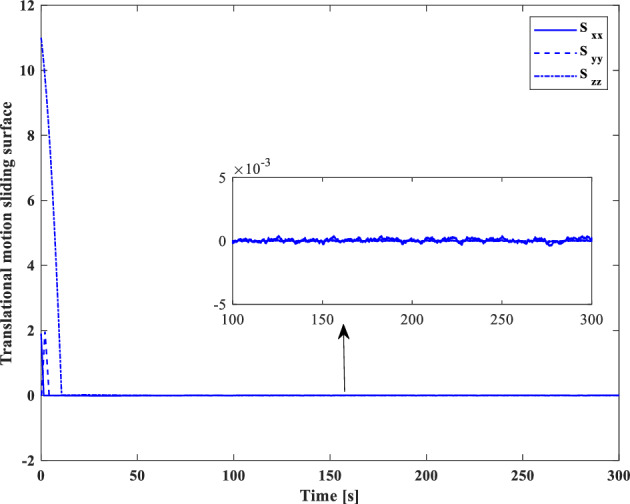
Translational motion sliding surface (Scenario 2).

Previous articles in the simulation stage dealt with scenarios similar to scenarios 1 (stable) and 2 (constant angular velocity) and tested their controller's performance under these two conditions. What is exciting and has not been investigated in previous studies is the accelerated rotational motion of the target presented in scenario 3.

### Scenario III

The third scenario is more challenging. In this case, the chaser spacecraft should approach an uncooperative 3-axis tumbling target with a high initial angular rate. In this scenario, the target satellite's angular velocity varies with time (accelerated motion), and this is the same movement that shows the power of the controller designed in this article. Both the chaser and the target are assumed to be in an LEO with 400 km perigee altitude. Orbit inclination is assumed to be $55 [deg]$. Eccentricity is set to 0.138, and the argument of perigee and right ascension of the ascending node (RAAN) are set to $270 [\text{deg}]$ and $0 [\text{deg}]$, respectively.

Initially, both the chaser and target spacecraft are located at the perigee with a relative position of ${\overrightarrow{r}}_{e}\left(0\right)={\left[60, 0, 25\right]}^{T} [\text{m}]$ and relative velocity of ${\overrightarrow{\dot{r}}}_{e}\left(0\right)=\left[3, -0.5, 5\right] [\frac{{\text{m}}}{{\text{sec}}}]$. Target’s initial angular velocity is ${\overrightarrow{\omega }}_{t}\left(0\right)=\left[\frac{\left(1-\wedge \right)\sigma }{cos\left(\gamma \right)}, 2\sigma \wedge ,1.2 \wedge \right] \left[\frac{{\text{rad}}}{{\text{sec}}}\right]$, and its angular acceleration is ${\overrightarrow{\dot{\omega }}}_{t}\left(0\right)=\left[0.23 {\omega }_{y}{\omega }_{z}, -29 {\omega }_{x}{\omega }_{z},0.063 {\omega }_{y}{\omega }_{x}\right] \left[\frac{{\text{rad}}}{{{\text{sec}}}^{2}}\right]$. The chaser's initial angular velocity is assumed to be ${\overrightarrow{\omega }}_{c}\left(0\right)=\left[0, 0, 0\right] \left[\frac{{\text{deg}}}{{\text{sec}}}\right]$. The initial attitude of the chaser and target spacecraft are preselected as ${\overrightarrow{q}}_{c}\left(0\right)={\left[-0.3257, 0.5957, 0, 6722, 0.2954\right]}^{T}$ and ${\overrightarrow{q}}_{t}\left(0\right)={\left[0.38831, 0.22356, -0.34408, 0.82512\right]}^{T}$ , respectively. where $\gamma =0.5$, $\wedge =1-\left({I}_{{t}_{22}}/{I}_{{t}_{11}}\right)=0.04556, and \sigma =0.1$.

Simulation results of the third scenario are discussed in the next.

**Figure 35:**
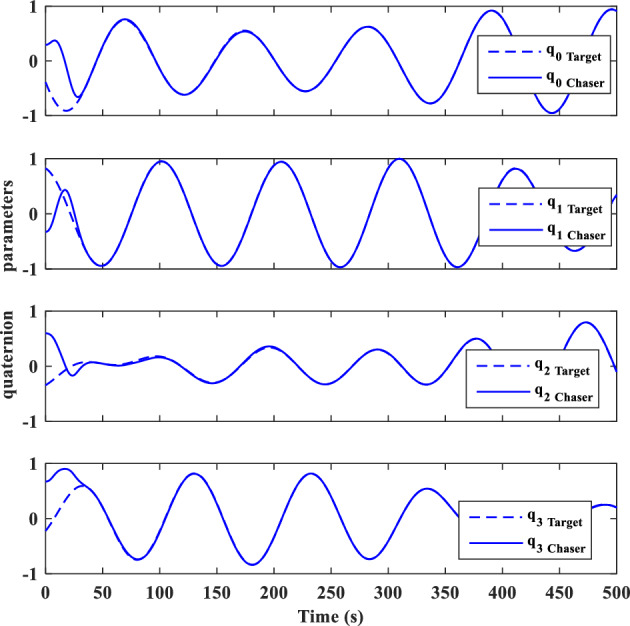
Chaser and target quaternion parameters (Scenario 3).

**Figure 36:**
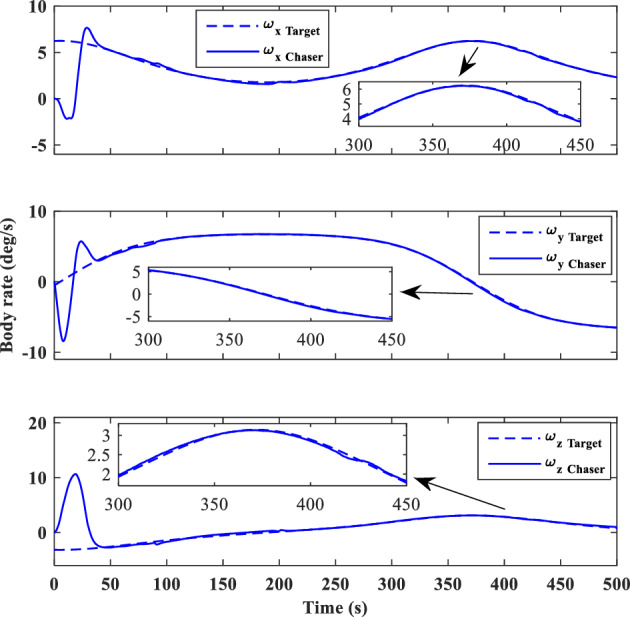
Chaser and target angular velocity (Scenario 3).

**Figure 37:**
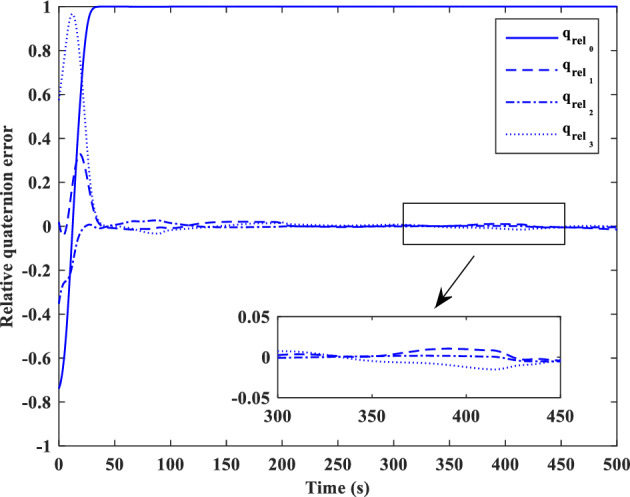
Relative quaternion parameters (Scenario 3).

**Figure 38:**
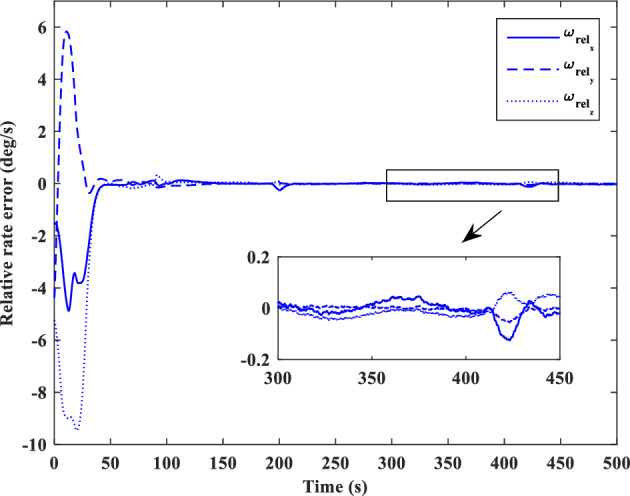
Relative angular velocity (Scenario 3).

Both chaser and target spacecraft quaternion parameters and angular velocities are shown in Figs. 35 and 36, respectively. These two plots show that the controller was able to synchronize the chaser’s orientation with the target. The attitude error and angular velocity error are also plotted in Figs. 37 and 38. The results show that tracking goals are achieved even under tough disturbance, uncertainty, actuators' delay and saturation, and accelerated motion.

**Figure 39:**
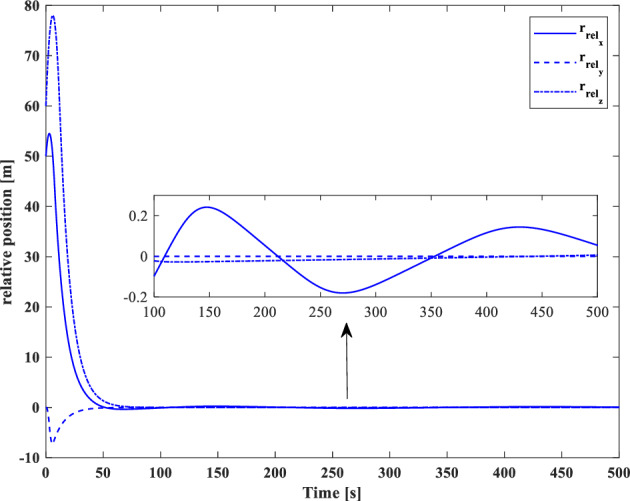
Relative position (Scenario 3).

**Figure 40:**
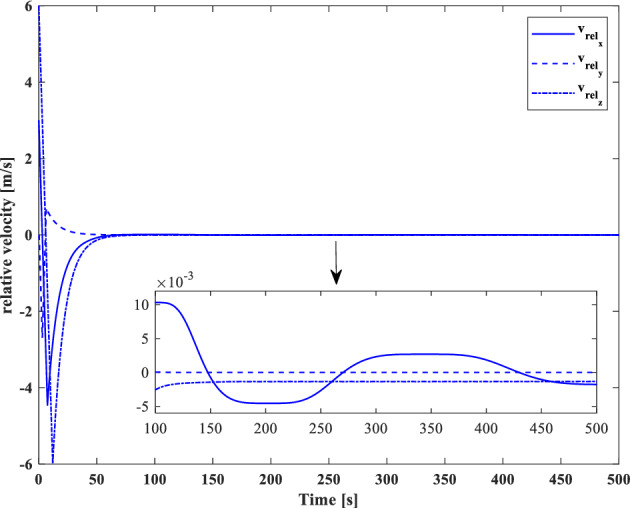
Relative velocity (Scenario 3).

The relative position and velocity of the two spacecraft are depicted in Figs. 39 and 40, respectively. According to these plots it can be claimed that the controller was successful in reducing relative distance along with tracking the target’s attitude.

**Figure 41:**
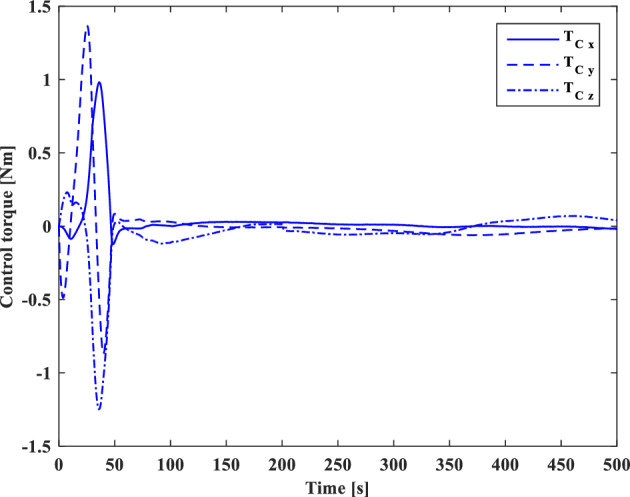
Attitude motion control effort (Scenario 3).

**Figure 42:**
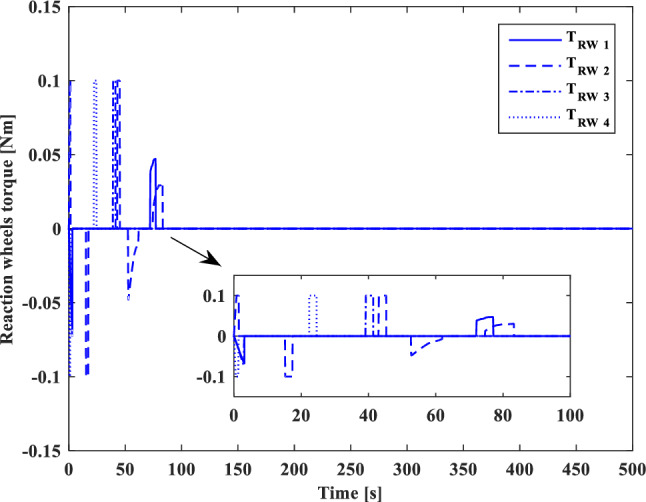
Reaction wheels torque (Scenario 3).

Control effort and reaction wheels torque are plotted in Figs. 41 and 42.

**Figure 43:**
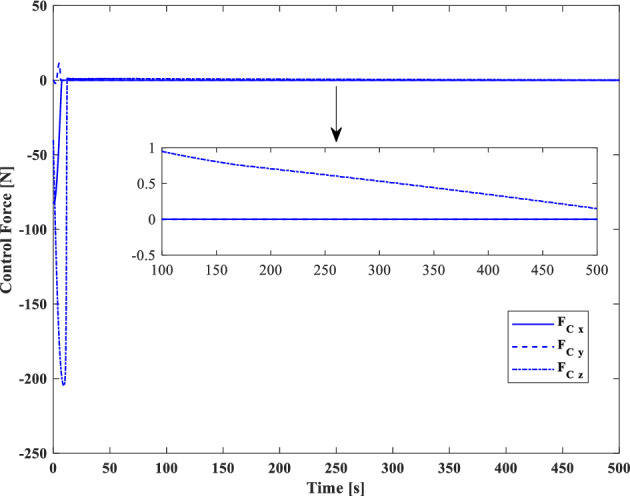
Translational motion control effort (Scenario 3).

**Figure 44:**
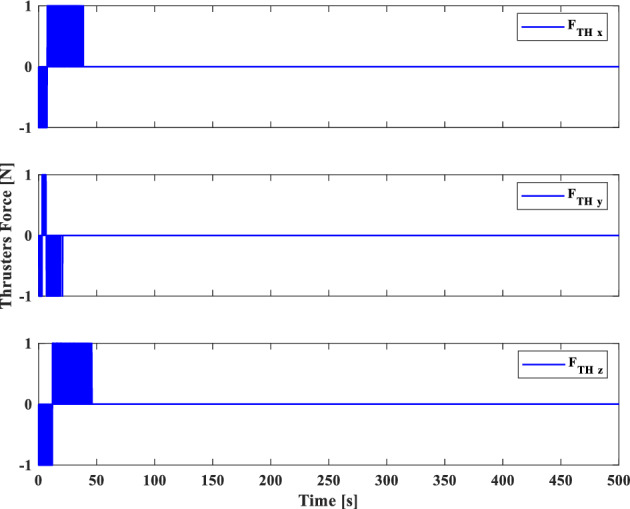
PWPF thruster’s force (Scenario 3).

Translational motion control effort and reaction thrusters force are depicted in Figs. 43 and 44.

**Figure 45:**
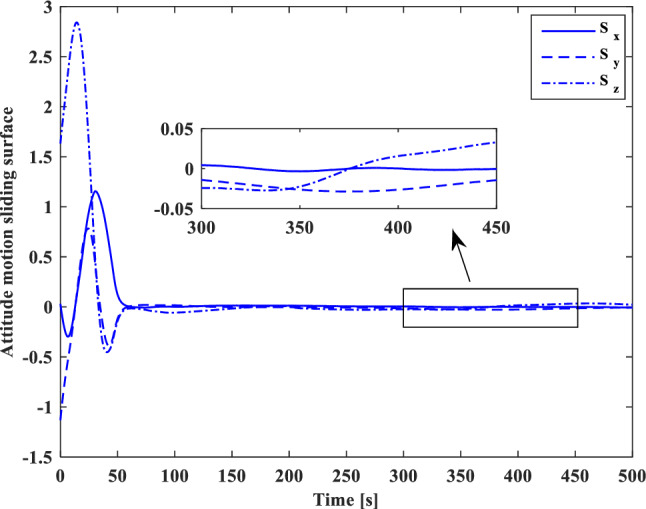
Attitude motion sliding surface (Scenario 3).

**Figure 46:**
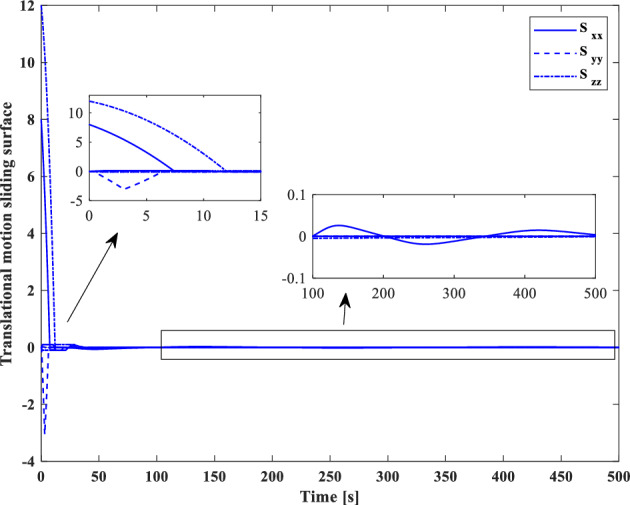
Translational motion sliding surface (Scenario 3).

The sliding surface of attitude and translational motion are shown in Figs. 45 and 46.

These three simulation scenarios demonstrate that the proposed control strategy is able to track the integrated position and attitude motion of the target spacecraft precisely both for (i) rest-to-rest and (ii) fast high-angle accelerated maneuver, under external and internal disturbances, parameter uncertainty, and actuator delay and saturation. In addition, a great advantage of the proposed controller is its implement-ability for OOS missions since it does not demand much computational load. The controller is also shown to be robust to extreme time-varying mass and moment of inertia uncertainties.

## Conclusion

This research specifically investigates the chaser’s approximation to a wide-body non-cooperative tumbling target. An important point about such targets is due to their huge size, they are susceptible to major disturbances and frequent incidents that cause their motion to accelerate. These dead bodies are typically equipped with various external appendages, including solar panels and antennas, which pose a significant challenge to their safe capture, particularly during accelerated motion. Thus, agility and the ability to perform accurate large-angle maneuvers are the primary requirements for capturing such targets. On the other hand, performing agile and large-angle maneuvers with high accuracy is really difficult due to the effects of dynamic nonlinearities and coupling, fuel slushing, actuator saturation, and parameter uncertainty that arise during such maneuvers.

To deal with the problem stated, an adaptive trajectory tracking control based on the sliding mode technique has been designed in this paper for space on-orbit servicing applications. The desired acceleration (target’s motion acceleration) can be regarded as an input to the control law, and this is a key advantage for capturing large debris that experience uncontrolled accelerated motion. In other words, the time-varying relative acceleration error can also be fed back to the controller. Parametric uncertainty (sharp, smooth, and periodic) and external disturbance are handled by the adaptation law and variable structure term, respectively. It is proven via the Lyapunov stability theorem that the tracking errors and adaptive parameters converge to zero asymptotically, under external disturbances. The proposed controller is easy to extend and implement in practice because it’s not time-consuming and is free from huge observers, filters, estimators, or iterative optimization loops. Also, there are no multiple control parameters for tuning, and the adaptation laws are not sensitive to specific initial conditions. There's no need to simplify the dynamic equations during the controller design phase because the controller algorithm is entirely compatible with the nonlinear equations of motion. From the modeling point of view, the reaction wheels’ term is considered in the relative attitude motion, and both reaction thrusters and reaction wheels' roles (delay, allocation, and saturation) are taken into account in the simulation phase. The relative kinematic equations are developed in such a way that ensures the servicing spacecraft’s docking port always points to the docking port of the tumbling target. Even the theoretical analysis and simulation examples prove that the proposed control scheme can satisfy the autonomous control requirements of close-range proximity missions under multiple strict conditions and scenarios.

## Data Availability

The authors confirm that all the data supporting the findings of this study are original and available within the article.

## Chaser data

$S\left(\Xi \right)$: Skew-symmetric matrix of vector $\Xi$
$\overrightarrow{B}$: Earth’s magnetic field [$\text{T}$]
$\mu$: Geocentric gravitational constant [${\text{km}}^{3}{\text{s}}^{-2}$]
${J}_{2}$: Second zonal harmonic
${\overrightarrow{\omega }}_{\oplus }$: Earth’s rotational velocity vector [$\text{rad}/\text{s}$]
${\mu }_{\otimes }$: Magnitude of the earth’s magnetic moment [${\text{Tm}}^{3}$]
${R}_{E}$: Radius of Earth [$\text{m}$]
$$\left[\phi , \theta , \psi \right]$$: Euler angles [$$\text{rad}$$]
$${m}_{C}$$: Chaser’s mass [$$\text{kg}$$]
${\overrightarrow{h}}_{tot}$: Total angular momentum of the chaser [$\text{Nms}$]
${I}_{c}$: Chaser’s moment of inertia [${\text{Nm}}^{2}$]
${\overrightarrow{r}}_{C}$: Chaser position vector [$\text{m}$]
${\overrightarrow{T}}_{c}$: Control torque acting on chaser [$\text{Nm}$]
${\overrightarrow{\omega }}_{c}$: Chaser’s angular velocity [$\text{rad}/\text{s}$]
${\overrightarrow{h}}_{c}$: Chaser’s angular momentum [$\text{Nms}$]
${\overrightarrow{q}}_{c}$: Chaser’s quaternion parameters [$\text{rad}$]
${n}_{c}$: Chaser’s orbital angular velocity [$\text{rad}/\text{s}$]
$a$: Semi-major axis [$\text{m}$]
${\overrightarrow{M}}_{tot}$: Total disturbance torque acting on chaser [$\text{Nm}$]
${\overrightarrow{M}}_{{r}_{c}}$: Random white nose
${\overrightarrow{M}}_{{GG}_{c}}$: Gravity Gradient (GG) torque acting on chaser $\text{Nm}$

## Target data

$${m}_{t}$$: Target’s mass [$$\text{kg}$$]
${I}_{t}$: Target’s moment of inertia [${\text{Nm}}^{2}$]
${\overrightarrow{r}}_{t}$: Target’s position vector [$\text{m}$]
${\overrightarrow{\omega }}_{t}$: Target’s angular velocity [$\text{rad}/\text{s}$]
${\overrightarrow{q}}_{T}$: Target’s quaternion parameters [$\text{rad}$]
${n}_{t}$: Target’s orbital angular velocity [$\text{rad}/\text{s}$]
${\overrightarrow{M}}_{{tot}_{t}}$: Moment of the magnetic dipole [${\text{Am}}^{2}$]
${\overrightarrow{M}}_{{MM}_{t}}$: Magnetic moment torque acting on target [$\text{Nm}$]
${\overrightarrow{M}}_{{GG}_{t}}$: Gravity Gradient (GG) torque acting on target [$\text{Nm}$]
${\overrightarrow{M}}_{{r}_{t}}$: Random white noise
${a}_{d}$: Relative disturbance acceleration [$\text{m}/{\text{s}}^{2}$]
${\overrightarrow{a}}_{gravity}$: Perturbing gravitational acceleration [$\text{m}/{\text{s}}^{2}$]
${\overrightarrow{a}}_{drag}$: Drag disturbance acceleration [$\text{m}/{\text{s}}^{2}$]
$$\rho$$: Atmosphere density [$$\text{kg}/{\text{m}}^{3}$$]
$${\rho }_{0}$$: Reference density [$$\text{kg}/{\text{m}}^{3}$$]
${h}_{ellp}$: Actual altitude of the spacecraft [$\text{m}$]
$${h}_{0}$$: Reference altitude [$$\text{m}$$]
$$H$$: Scale height $$\text{m}$$

## Disturbance data

$S$: Surface normal to the atmosphere flow [${\text{m}}^{2}$]
$${C}_{D}$$: Drag coefficient
${\overrightarrow{v}}_{D}$: Spacecraft velocity relative to the atmosphere velocity [$\text{m}/\text{s}$]
$${\overrightarrow{v}}_{atm}$$: Atmosphere velocity [$$\text{m}/\text{s}$$]
$\mathsf{\Theta }$: The magnetic latitude $\text{deg}$

## Relative data

${C}_{t}^{i}$: Rotation matrix from target to interface frame
${C}_{t}^{c}$: Rotation matrix from target to chaser body frame
${C}_{i}^{t}$: Rotation matrix from interface to target body frame
${C}_{c}^{LVLH}$: Rotation matrix from LVLH to chaser body frame [$\text{rad}/\text{s}$]
${\overrightarrow{\omega }}_{e}$: Error angular velocity
${\overrightarrow{\omega }}_{i}$: Interface frame angular velocity [$\text{rad}/\text{s}$]
$${\overrightarrow{q}}_{r}$$: Relative quaternion [$$\text{rad}$$]
$${\overrightarrow{q}}_{e}$$: Error quaternion [$$\text{rad}$$]
$${r}_{e}$$: Relative distance [$$\text{rad}$$]
$${M}_{e}$$: Mean anomaly
$$E$$: Eccentric anomaly [$$\text{rad}$$]
$$e$$: Eccentricity
$${\psi }_{true}$$: True anomaly $$\text{rad}$$

## Actuators data

${F}_{c}$: Control force acting on chaser [$\text{N}$]
${\text{T}}_{\text{s}}$: Thrusters time-constant [$\text{s}$]
$${\text{I}}_{\text{sp}}$$: Specific impulse [$$\text{s}$$]
$\left[{A}^{*}\right]$: Reaction wheels torque distribution matrix
${\overrightarrow{T}}_{{w}_{i}}$: Each reaction wheel torque [${\text{Nm}}^{2}$]
${\omega }_{{w}_{max}}$: Maximum angular velocity of the reaction wheel [$\text{rpm}$]
${T}_{{w}_{max}}$: Maximum torque of the reaction wheels [$\text{Nm}$]
${I}_{w}$: Reaction wheel’s moment of inertia [${\text{kgm}}^{2}$]
${\overrightarrow{h}}_{rw}$: Reaction wheel’s angular momentum $\text{Nms}$

## Controller data

${S}_{{aug}_{x}}, {S}_{{aug}_{y}}, {S}_{{aug}_{z}}$: Attitude motion controller augmented sliding surface
${s}_{x}, {s}_{y}, {s}_{z}$: Attitude motion controller sliding surface
${e}_{x}, {e}_{y}, {e}_{z}$: Relative attitude error [$\text{rad}$]
${V}_{x}, {V}_{y}, {V}_{z}$: Attitude motion controller Lyapunov function
${e}_{xx}, {e}_{yy}, {e}_{zz}$: Relative position error [$\text{m}$]
${V}_{xx}, {V}_{yy}, {V}_{zz}$: Translational motion controller Lyapunov function
${S}_{{aug}_{xx}}, {S}_{{aug}_{yy}}, {S}_{{aug}_{zz}}$: Translational motion controller augmented sliding surface
${s}_{xx}, {s}_{yy}, {s}_{zz}$: Translational motion controller sliding surface

## Desired data

${\ddot{x}}_{d}, {\ddot{y}}_{d}, {\ddot{z}}_{d}$: Desired relative translational acceleration [$\text{m}/{\text{s}}^{2}$]
${\dot{\omega }}_{{e}_{{x}_{d}}}, {\dot{\omega }}_{{e}_{{y}_{d}}}, {\dot{\omega }}_{{e}_{{z}_{d}}}$: Desired relative orientational acceleration $\text{rad}/{\text{s}}^{2}]$

## Subscripts

c: Refers to the chaser spacecraft
t: Refers to the target spacecraft
r: Refers to the relative quantity
e: Refers to the error quantity
